# Tuned inhibition in perceptual decision-making circuits can explain seemingly suboptimal confidence behavior

**DOI:** 10.1371/journal.pcbi.1008779

**Published:** 2021-03-29

**Authors:** Brian Maniscalco, Brian Odegaard, Piercesare Grimaldi, Seong Hah Cho, Michele A. Basso, Hakwan Lau, Megan A. K. Peters

**Affiliations:** 1 Department of Cognitive Sciences, University of California Irvine, Irvine, California, United States of America; 2 Department of Bioengineering, University of California Riverside, Riverside, California, United States of America; 3 Department of Psychology, University of Florida, Gainesville, Florida, United States of America; 4 Department of Psychology, University of California Los Angeles, Los Angeles, California, United States of America; 5 Fuster Laboratory of Cognitive Neuroscience, Department of Psychiatry and Biobehavioral Sciences, University of California Los Angeles, Los Angeles, California, United States of America; 6 Department of Psychology, University of Hong Kong, Pokfulam, Hong Kong SAR; 7 The Jane and Terry Semel Institute for Neuroscience and Human Behavior, University of California Los Angeles, Los Angeles, California, United States of America; 8 Brain Research Institute, University of California Los Angeles, Los Angeles, California, United States of America; 9 State Key Laboratory of Brain and Cognitive Sciences, University of Hong Kong, Pokfulam, Hong Kong, SAR; 10 Interdepartmental Graduate Program in Neuroscience, University of California Riverside, Riverside, California, United States of America; 11 Department of Psychology, University of California Riverside, Riverside, California, United States of America; Duke University, UNITED STATES

## Abstract

Current dominant views hold that perceptual confidence reflects the probability that a decision is correct. Although these views have enjoyed some empirical support, recent behavioral results indicate that confidence and the probability of being correct can be dissociated. An alternative hypothesis suggests that confidence instead reflects the magnitude of evidence in favor of a decision while being relatively insensitive to the evidence opposing the decision. We considered how this alternative hypothesis might be biologically instantiated by developing a simple neural network model incorporating a known property of sensory neurons: tuned inhibition. The key idea of the model is that the level of inhibition that each accumulator unit receives from units with the opposite tuning preference, i.e. its inhibition ‘tuning’, dictates its contribution to perceptual decisions versus confidence judgments, such that units with higher tuned inhibition (computing relative evidence for different perceptual interpretations) determine perceptual discrimination decisions, and units with lower tuned inhibition (computing absolute evidence) determine confidence. We demonstrate that this biologically plausible model can account for several counterintuitive findings reported in the literature where confidence and decision accuracy dissociate. By comparing model fits, we further demonstrate that a full complement of behavioral data across several previously published experimental results—including accuracy, reaction time, mean confidence, and metacognitive sensitivity—is best accounted for when confidence is computed from units without, rather than units with, tuned inhibition. Finally, we discuss predictions of our results and model for future neurobiological studies. These findings suggest that the brain has developed and implements this alternative, heuristic theory of perceptual confidence computation by relying on the diversity of neural resources available.

## Author summary

The dominant view of perceptual confidence proposes that confidence optimally reflects the probability that a decision is correct. But recent empirical evidence suggests that perceptual confidence exhibits a suboptimal ‘confirmation bias’, just as in human decision-making in general. We tested how this ‘bias’ might be neurally implemented by building a biologically plausible neural network model, and showed that the ‘bias’ emerges when each neuron’s degree of inhibition received from neurons with opposing tuning preferences dictates how it drives decisions versus confidence judgments. We additionally showed that alternative models lacking this architecture fail to capture known behavioral effects. These results challenge the dominant model, suggesting that the brain instead capitalizes on the diversity of available machinery (i.e., neuronal resources) to implement *heuristic*—not optimal—strategies to compute subjective confidence.

## 1. Introduction

A dominant idea in the study of perceptual decision-making is that confidence judgments optimally reflect the probability that a decision is correct [1–5]. Several models specifically stipulate that confidence is calculated via implementation of a diffusion framework: a decision is made when evidence for a decision reaches a certain threshold, and confidence reflects an optimal readout of the same information [3–9].

While this optimal ‘probability correct’ account of confidence has enjoyed significant empirical support, it seems difficult for it to account for cases where task performance and confidence dissociate [10–18]. Seemingly suboptimal behaviors have also been observed in post-decisional perceptual judgments other than confidence [19,20], leading these authors to hypothesize that these suboptimalities may stem from limitations on computational (i.e., neural) resources or a drive towards self-consistent behavior. One alternative theory of confidence, therefore, proposes that subjective confidence relies primarily on the magnitude of evidence supporting an observer’s decision, while ignoring or downplaying evidence supporting alternative, unchosen decisions [10,14,16,18,21,22]. In other words, to compute confidence the system uses a suboptimal heuristic that overly relies on decision-congruent evidence magnitude rather than optimal computations. Indeed, a recent study reported evidence for these decision-congruent evidence confidence computations using human intracranial electrocorticography [23].

However, to date no biologically plausible mechanism has been proposed that might explain these dissociations between confidence and performance, or the decision-congruent confidence computations on which they seem to depend. We therefore developed a simple dynamic evidence accumulation network model to test a new hypothesis of how these computations might be implemented. This model extends previous work to incorporate a known property of perceptual circuitry: *tuned normalization* [24–26], meaning each neuron is characterized by the specific degree to which it is normalized by surrounding or nearby network activity [27,28], and specifically by units with opposing tuning preferences. In the present work, we use the more general term *tuned inhibition* to refer to any neural dynamics in which neurons are preferentially inhibited by other neurons with opposing tuning preferences, regardless of whether such dynamics are divisive in nature (as normalization processes are typically characterized to be), since our primary hypothesis concerns the general phenomenon of tuned inhibition without a particular concern for whether such inhibition is divisive or not. We hypothesized that each neuron’s degree of tuned inhibition dictates how it differentially participates in discrimination decisions versus confidence judgments. Specifically, we reasoned that strongly inhibited ‘differencing’ neurons encode the balance of evidence for various perceptual interpretations (e.g. net accumulated evidence for leftwards or rightwards motion direction), and thus are reasonable candidates for making discrimination judgments. By contrast, less inhibited evidence accumulation neurons encode total overall evidence in favor of one perceptual interpretation (e.g. leftward motion) while ignoring evidence for alternative interpretations (e.g. rightward motion), and thus are reasonable candidates for implementing decision-congruent confidence computations. Therefore, the simple design principle that more inhibited differencing neurons drive decisions and less inhibited accumulator neurons drive confidence may be sufficient to account for some of the most counterintuitive empirical findings on confidence in perceptual decision-making.

We tested key predictions of a *Differential Tuned Inhibition* model instantiating this hypothesis using computational modeling, with exploratory supplemental results from single neuron physiology. The computational model simulations show strong support for our hypothesis: the model reproduces multiple empirical findings when confidence is computed primarily from less inhibited ‘absolute evidence’ units, but not when computed primarily from more inhibited ‘differencing’ units. With this approach, we extend previous conceptual insights to include dynamical evidence accumulation and reaction time effects, while also unifying multiple empirical datasets across multiple paradigms with a single coherent approach. We also show that meta-d’, a measure of metacognitive sensitivity [29], provides a crucial target for model comparisons of perceptual confidence. Our results suggest that tuned inhibition may play a crucial role in how the brain differentially computes perceptual decisions and subjective confidence–thus revealing an important psychological function of this neuronal property.

## 2. Methods

### 2.1. Ethics statement

In S4 Text, we describe exploratory behavioral and electrophysiology results from the superior colliculus for one male rhesus monkey. Details of the surgical procedures used to implant electrodes are also provided in S4 Text. All experimental protocols were approved by the UCLA Chancellor’s Animal Research Committee (IACUC, protocol number 2012–043) and complied with and generally exceeded standards set by the Public Health Service policy on the humane care and use of laboratory animals.

### 2.2. The *Differential Tuned Inhibition* model

The model presented here is a dynamic evidence accumulation network with tuned inhibition, where units with different inhibition tuning differentially contribute to perceptual decisions and confidence.

To investigate how decision-congruent evidence might be biologically implemented, we began by considering known properties of perceptual decision-making circuitry. It is well known that normalization is a canonical neural computation throughout the cortex [27,28,30–34]. Further, it was recently reported that neurons in primary sensory areas exhibit *tuned normalization* (here called *tuned inhibition* for generality), i.e. that each neuron possesses a unique, consistent degree of received inhibition: some neurons are very sensitive to activity of other units in the network (especially those which have different tuning preferences), while others operate more independently [24–26]. Previous implementations of evidence accumulation models for perceptual decisions have typically considered how a single level of normalization or inhibition—in a single set of units that drive both perceptual decisions and confidence—can account for behavioral data [3–8,21,35]. However, we now know that a range of inhibition tuning exists, at least in sensory cortices. We hypothesized that these neuron-by-neuron variations in inhibition may reflect not noise or measurement error, but meaningful properties of the perceptual decision-making circuitry [24–26]. We refer to this model as the *Differential Tuned Inhibition* model, or the *Tuned Inhibition* model for short.

But *how* might tuned inhibition be utilized in a behaving organism? To answer this, we should consider the tasks an organism must successfully execute in an ecologically valid environment. The ability to discriminate among multiple possible stimulus identities is certainly important, and for this type of task an observer ought to rely on a system that is able to average out noise, i.e. is less susceptible to random fluctuations in signal. Thus, for these discrimination-type tasks, a strong degree of inhibition would be desirable, as it has been shown that neurons with stronger tuned inhibition do exhibit weaker pairwise correlations [25,26]. But it is equally important that an organism also be able to detect a stimulus in the first place, regardless of its identity. For these detection-type tasks, such strong inhibition would actually be undesirable, as minute evidence amounts may be informative; therefore, weakly inhibited neurons ought to play a stronger role in detection-type tasks. As both of these task types are critical for an organism’s survival, it seems unlikely that a system would only be optimized for one or the other, which could in theory explain the presence of tuned inhibition.

In light of this discussion, and of the empirically observed tuned inhibition in cortical areas, a biologically plausible model of sensory evidence accumulation ought to implement more than one level of inhibition and consider how such variations in inhibition tuning may affect a neuron’s role in the circuitry. Further, such stratification of tuned inhibition could provide a neural mechanism to explain findings that confidence judgments rely on the magnitude of decision-congruent evidence [10,14,17,18,21–23]. Specifically, the output of less inhibited ‘detection’ neurons could be used to index decision-congruent evidence and therefore be used for confidence rating. This suggests that inhibition tuning provides a biologically plausible mechanism to keep track of decision-congruent evidence independently of evidence favoring other possible choices by relying on the less inhibited portion of the circuitry, while allowing the system to still capitalize on the beneficial consequences of inhibition by opposingly-tuned neurons by relying on the more inhibited portion when discriminating among possible stimulus identities. We therefore hypothesized that inhibition tuning might specifically dictate a neuron’s contribution to discrimination versus confidence judgments in decision-making circuitry.

**Fig 1:**
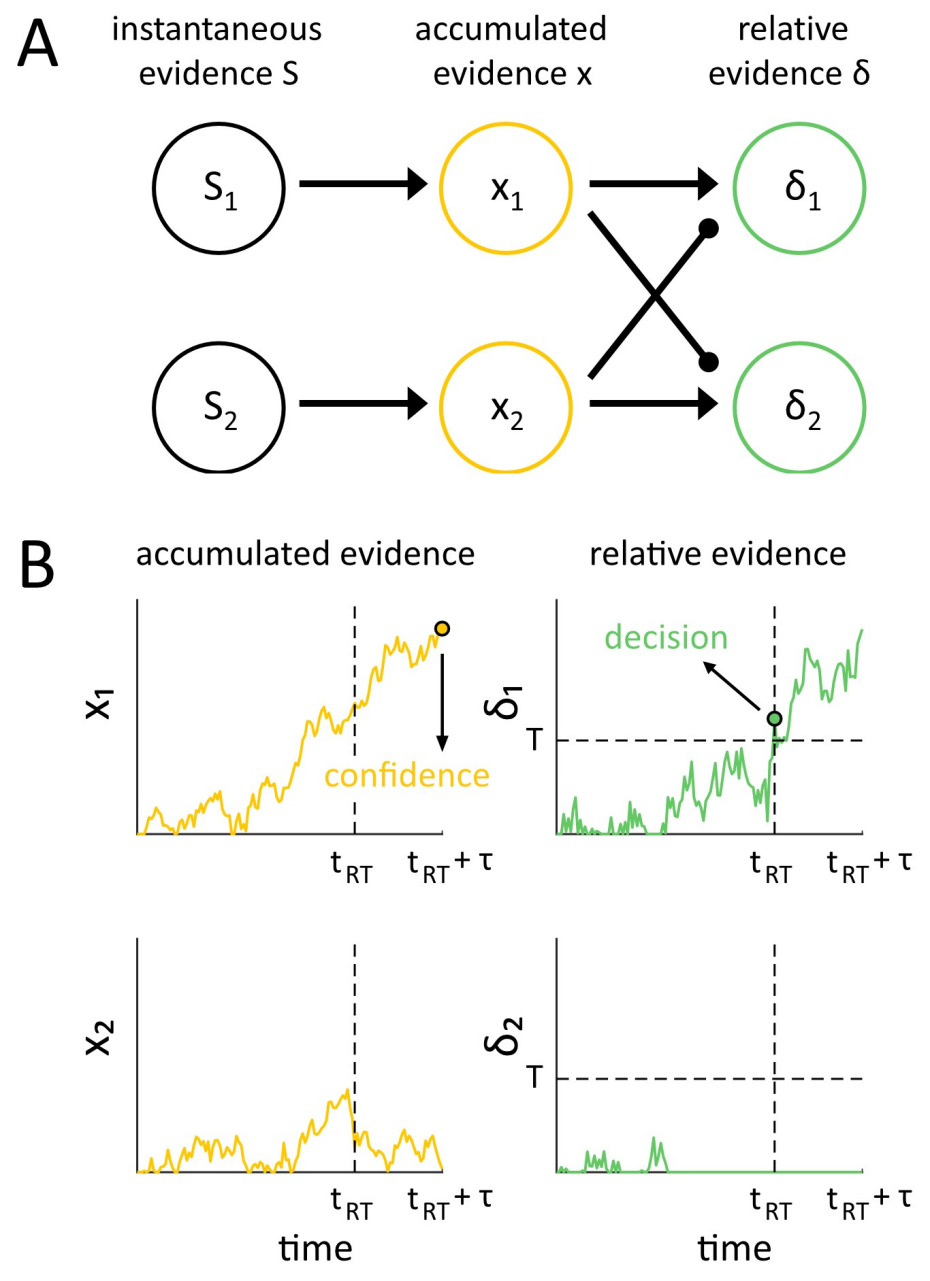
The Differential Tuned Inhibition model diagram and sample activity traces. (A) In the 2-stage model, instantaneous evidence from a source *S*_*i*_ is accumulated by independent accumulators *x*_*i*_ tuned to each stimulus type *i*. Differencing units *δ*_*i*_ then compute the difference in accumulated evidence for each stimulus alternative, implemented through feed-forward excitation from units with the same preferences and inhibition from accumulator units with opposing preferences. (B) A decision is made by the model when the activity of one of the differencing units hits a bound, i.e. when enough relative evidence for one stimulus over another has been accumulated. Confidence in the main Differential Tuned Inhibition model is then read out after delay *τ* from the independent accumulator x_D_ corresponding to the decision D that was made. In an alternative model, we also tested how this framework might perform if confidence were read out from the differencing unit *δ*_*D*_; see Methods for details.

We examined this hypothesis by incorporating tuned inhibition [24,25] into a dynamic evidence accumulation network (Fig 1). Intuitively, this network’s architecture can be summarized as follows. Accumulator units tuned to varying stimulus alternatives accumulate momentary stimulus evidence. Downstream ‘differencing’ units receive excitatory and inhibitory input from accumulator units having opposing tuning preferences, effectively performing a subtraction to yield the balance of evidence favoring one stimulus alternative over the other. A discrimination decision is made when a differencing unit with a given tuning preference reaches a threshold level of evidence.

Following the perceptual decision, additional evidence accumulation occurs in order to form a confidence judgment [9]. Confidence is then evaluated by comparing accumulator unit activity for the chosen stimulus alternative to a set of decision thresholds for rating confidence. The duration τ of this post-decision evidence accumulation controls metacognitive sensitivity, i.e. the efficacy with which confidence ratings discriminate correct from incorrect responses, as measured e.g. by the signal detection theory measure meta-d’ [29]. In general, longer post-decision accumulation periods yield higher metacognitive sensitivity (consistent with empirical patterns reported in [36]). When τ = 0 and confidence is determined at the same instant as the perceptual decision, there is very little variability in perceptual evidence values (since, by definition, the perceptual decision is made when perceptual evidence in the differencing units arrives at a fixed threshold value), which has the consequence that meta-d’ ≈ 0 (see e.g. S1C and S1D Fig), meaning confidence is at chance levels of discriminating correct from incorrect responses. Thus, incorporating a post-decision evidence accumulation stage with τ > 0 in this kind of evidence accumulation model is important to capture empirical patterns of metacognitive sensitivity.

For simplicity, we assume that evidence accumulation rates remain constant in the post-decision stage. This assumption need not imply that the physical stimulus remains available to perception for the duration of the decision process, as evidence accumulation could depend in part on internal processes that continue even after stimulus offset. Consistent with this idea, prior work has demonstrated that for briefly presented and masked stimuli, diffusion models assuming constant drift rate fit the data better than ones assuming drift rates that vary with stimulus duration and mask onset, suggesting that evidence accumulation rates can persist over time even after stimulus offset [37].

Crucially, the roles of these unit types in the perceptual decision making process depends on their level of inhibition tuning, and this weighting differs for discrimination decisions and confidence ratings. More inhibited ‘differencing’ units determine discrimination decisions, since they effectively encode the accumulated balance of evidence for one stimulus alternative versus the others by virtue of their receipt of inhibition from units with opposing tuning preferences. By contrast, less inhibited accumulator units determine perceptual confidence, since they effectively encode a “pure” representation of the raw magnitude of independent evidence supporting each decision alternative, regardless of evidence supporting other possible decisions. Details of model implementation and all simulations follow in the next sections.

### 2.3. Model specifics

The Differential Tuned Inhibition model consists of two main stages of processing: (1) an initial evidence accumulation stage, in which independent sensory evidence for different perceptual interpretations is independently integrated over time, and (2) a subsequent evidence comparison stage, in which the independently-accumulated evidence for perceptual alternatives in the previous stage is directly compared by a subtractive (differencing) process which could be biologically implemented through feed-forward inhibition (Fig 1).

To capture perceptual dynamics in experiments in which an observer must use noisy sensory evidence to decide between two perceptual alternatives (e.g. deciding if a grating is tilted left or right, if dot motion is moving leftwards or rightwards, etc.), we model two independent accumulator units (which can be thought of as corresponding to two independent pools of unnormalized accumulator neurons) corresponding to each perceptual alternative, where each evolves according to the equation:

$$x_{i}(t)=max({x_{i}(t-1)+S}_{i}+\varepsilon_{i}(t),0)$$ (1)

In this equation, i denotes stimulus alternative, S_i_ denotes instantaneous evidence for stimulus i (assumed to be constant over time), and ε_i_(t) is a noise term such that ε_i_(t) ~ N(0, σ). The max operation rectifies the accumulator unit to ensure non-negative values.

At the second stage, the balance of evidence supporting stimulus i relative to stimulus j is computed as the instantaneous difference between the evidence accumulated thus far for i and j:

$$\delta_{i}(t)=max(x_{i}(t)-x_{j}(t)+\xi_{i}(t),0)$$ (2)

where ξ_i_(t) is a noise term such that ξ_i_(t) ~ N(0, σ). (For simplicity, we assume that noise terms at each level of processing, ε_i_(t) and ξ_i_(t), are drawn from the same distribution.) Once again, the results are rectified to ensure non-negative values. Evidence accumulation proceeds until one of the strongly inhibited ‘differencing’ units δ_i_ achieves some threshold value T, at which point the observer decides upon perceptual interpretation i as their decision D for this trial:

$$D=\left\{ \begin{array}{c} i\;if\;\delta_{i}(t)>T\\ j\;if\;\delta_{j}(t)>T \end{array}\right.$$ (3)

Reaction time t_RT_ is considered to correspond to the value of t at which δ_D_ first surpasses T.

After the initial perceptual decision is made, evidence accumulation in the uninhibited units continues for a fixed number of time steps τ, following previous convention [9]. Once τ time steps have passed, confidence is read out as the value of evidence in the uninhibited accumulator unit corresponding to the perceptual decision, i.e.

$$C_{x}=x_{D}(t_{RT}+\tau )$$ (4)

Thus, whereas the perceptual decision D depends on the balance of evidence between stimulus alternatives δ_D_, confidence C_x_ depends on the total amount of “uninhibited” or “absolute” accumulated evidence in favor of the perceptual decision, x_D_. This divergence in the computation of perceptual decision and confidence embodies the decision-congruent evidence effect discovered in empirical studies and captures the central phenomenon of interest for this manuscript.

To directly test the necessity of this model architecture for capturing the behavioral effects reported in the literature, we also implement an alternative control model consistent with dominant theory that decisions and confidence judgments are computed from the same units, i.e.

$$C_{\delta }=\delta_{D}(t_{RT}+\tau )$$ (5)

This alternative model is logically consistent with canonical models for computing confidence from the accumulated balance of evidence (e.g., [3–5]).

It is important at this point to clarify a point of potential misunderstanding. Note that in both Eq 4 and Eq 5, confidence is read out from a decision unit whose tuning preference matches the perceptual decision (denoted by the D subscripts in both equations). Thus, on one possible reading of the term “decision congruent evidence,” one might conclude that confidence depends on decision congruent evidence in both Eqs 4 and 5, since in both cases, confidence is read out from a unit whose stimulus tuning preference matches the perceptual decision. However, when we use the term “decision congruent evidence” in this manuscript, consistent with prior usage in the literature, we mean to refer to evidence that supports the perceptual decision one has chosen, independent of evidence for alternative choices. In this sense of the term, only Eq 4 qualifies as computing confidence from decision-congruent evidence, since only in this case is confidence insensitive to evidence for the alternative, unchosen perceptual interpretation(s).

For both the main Tuned Inhibition C_x_ model and the alternative C_δ_ model, we compare model outputs to empirical confidence data by converting raw confidence C values to an ordinal rating scale value R by comparing the respective Cs to a series of confidence threshold values U_r_, as follows:

$$R=1+\sum_{r=1}^{N_{r-1}}\left(C>U_{r}\right)$$ (6)

where N_r_ is the number of rating scale options available (e.g. N_r_ = 4 on a 4-point confidence scale), and (C > U_r_) is a logical comparison evaluating to 1 if the inequality is true and 0 otherwise. Thus, R is a simple count of how many of the confidence thresholds U_r_ are surpassed by C, with the constant 1 added to set 1 as the minimum confidence rating value.

### 2.4. Simulations

We tested the model by assessing its ability to capture empirical dissociations between perceptual task performance and confidence in three representative data sets: a dissociation between d’ and meta-d’ (the trial-by-trial correspondence between accuracy and confidence [29]) observed in Maniscalco, Peters, & Lau [14], and dissociations between d’ and mean confidence observed in Experiments 1A and 2B of Koizumi, Maniscalco, & Lau [10]. Details of the experimental designs and simulation results are discussed below.

For each data set, we fit the Differential Tuned Inhibition model (with confidence computed from absolute evidence accumulators) and the alternative model (with confidence computed from the difference between accumulators) to the data and compared the ability of the models to capture the relevant dissociations. In connection with Eqs 4 and 5, we call these the C_x_ model and C_δ_ model for short, respectively. We expected that the C_x_ model would outperform the C_δ_ model in its ability to capture the dissociations, thus lending further support to the hypothesis that confidence depends primarily on decision-congruent evidence.

We followed a similar approach in fitting the models to these diverse data sets, as outlined below, though certain details of model fitting were particular to each data set. Full details for model fitting are provided in S1 Text.

#### 2.4.1. Selecting values for σ and T

First, we arbitrarily set accumulation noise σ = 0.1. The choice of this parameter value is arbitrary since, if no parameter values are fixed, identical simulation results can be obtained by a simple scaling of the model parameters. We then set decision threshold T = 1 to ensure that, even in the absence of stimulus drive S (i.e. S_1_ = S_2_ = 0), accumulation of noise alone could reach T within a reasonable number of time steps, while still ensuring that at least several time steps must pass for this to occur (in 10 repetitions of simulations with 10,000 trials each, average median RT = 80.6 and average minimum RT = 7.3). These choices for σ and T formed a fixed reference against which other parameters of the model could be optimized. We found that similar simulation results occurred when using different values for T, which can be readily verified using the simulation code available online.

#### 2.4.2. Fitting S to d’

Next, for each data point in the data set, we found what value of stimulus drive S would be required to yield the desired value of d’. We did this by performing simulations at 10 evenly spaced values of S and fitting a quadratic polynomial to the resulting d’ vs S curve, which provided an excellent fit across a range of d’ values from ~ 0–3 (S1A and S1B Fig). Using the fitted polynomial equation, we could solve for what value of S yielded the value of d’ to be fitted.

#### 2.4.3. Fitting U_r_ to confidence probability distributions and τ to meta-d’

The parameter τ determines how many time steps pass after the initial perceptual decision is made, during which evidence continues to accumulate before a confidence judgment is formed. Larger values of τ correspond to more evidence accumulation prior to confidence rating and therefore higher values of meta-d’ (i.e., confidence ratings that carry more information about task accuracy). Thus, empirically observed values of meta-d’ can serve as a guide for appropriate values of τ.

In each data set simulation, we fit τ to the meta-d’ value of only one representative data point, and used this value of τ for all subsequent simulations of that data set. This approach ensured that τ was held constant across all conditions. Importantly, as a consequence, the fitting procedure guaranteed a close fit to meta-d’ in only one data point, and simulated meta-d’ values at all other data points were unconstrained by further parameter fitting and instead arose as a consequence of the value of τ fitted to the representative meta-d’ value.

To accomplish the fit, we performed simulations at 10 evenly spaced values of τ and fitted a quadratic polynomial to the resulting meta-d’ vs τ curve, which provided an excellent fit across a range of meta-d’ values from ~ 0 –d’ (S1C and S1D Fig). Using the fitted polynomial equation, we could solve for what value of τ yielded the value of meta-d’ to be fitted for the single fitted data point.

In order to compute meta-d’, continuous confidence values (C_x_ or C_δ_, depending on the model being used) first had to be converted to a 4-point rating scale (corresponding to the 4-point confidence rating scale used in all three data sets to be fitted), which in turn required specifying the values of the confidence thresholds U_r_. For each simulation, we set U_r_ such that the probability distribution of simulated confidence ratings across all trials of all conditions exactly matched the corresponding empirical probability distribution. More formally, we computed U_r_ as

$$U_{r}=quantile(C,\sum_{i=1}^{r}P_{data}(conf=i))$$ (7)

where C corresponds to C_x_ or C_δ_, depending on the model being used, quantile(C, p) returns the quantile of the distribution C corresponding to the cumulative probability p, and P_data_(conf = i) is the empirical probability distribution of confidence ratings.

As noted above, full details of the model fitting procedures are provided in S1 Text.

## 3. Results

### 3.1. Simulating data from Maniscalco, Peters, & Lau 2016 [14]

**Fig 2:**
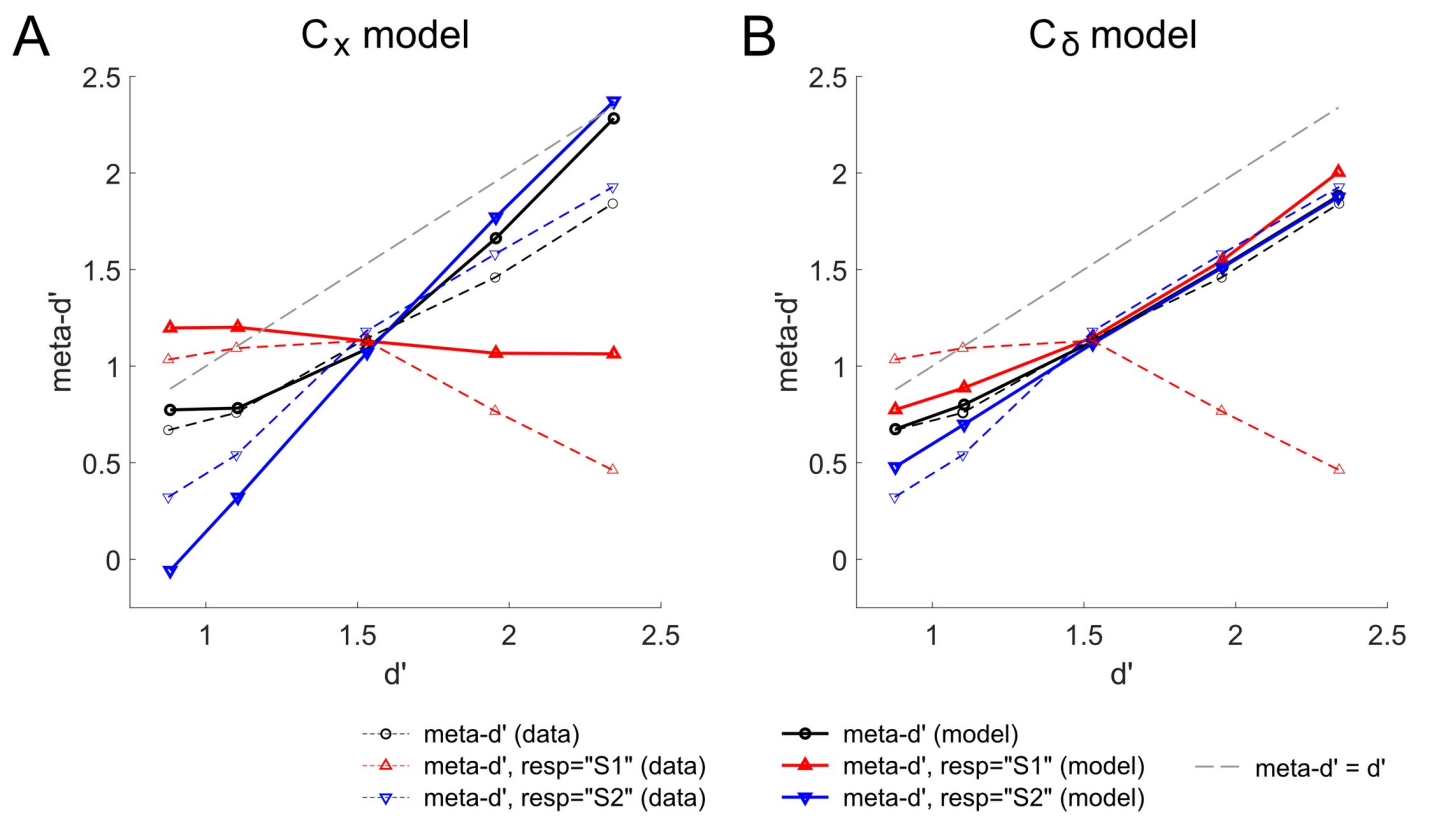
Modeling results for the effects reported by Maniscalco and colleagues [14]. The fitted main C_x_ model (A) reproduces the X-shaped crossover in response-conditional meta-d’ that was observed in the empirical data: as d’ increases, response-conditional meta-d’ for “S2” responses increases whereas meta-d’ for “S1” responses decreases. In contrast, such an X-shaped crossover in response-conditional meta-d’ is not even qualitatively present for the alternative C_δ_ model (B).

Maniscalco, Peters, & Lau [14] used signal detection theory modeling to predict that, if human observers do indeed use decision-congruent evidence to judge confidence, then under certain conditions it should be possible to observe a counterintuitive dissociation whereby metacognitive sensitivity *decreases* even as perceptual task performance *increases*. They conducted an experiment to test the prediction and verified that human observers did indeed exhibit this surprising dissociation (Fig 4 of [14]; results reproduced in Fig 2), providing strong support for the decision-congruent evidence model of confidence.

The experiment used a simple 2IFC task in which circular noise patches were presented to the left and right of fixation on every trial, with a grating embedded in the left or right noise patch. Subjects had to report which side the grating was on and then rate confidence on a 4-point scale. Crucially, the contrast of gratings presented on one side of the screen was held constant (call it C_S1_), while the contrast of gratings presented on the other side could take on one of 5 possible values C_S2_. C_S2_ spanned a range of values below and above C_S1_, with the intermediate value of C_S2_ equal to C_S1_. Briefly, the logic behind the predicted dissociation is as follows: If subjects rate confidence according to decision-congruent evidence, then it follows from a two-dimensional signal detection theory model that incorrect “S1” responses should yield higher mean confidence as C_S2_ increases. Since C_S1_ is constant, correct “S1” responses have a fixed level of mean confidence. Thus, the net effect of increasing C_S2_ is to increase task performance (d’) while simultaneously increasing confidence in incorrect “S1” responses but not correct “S1” responses, leading to a decrease in metacognitive sensitivity (meta-d’) for “S1” responses. This predicted pattern in confidence for incorrect “S1” responses is indeed what was observed (S3 Fig of [14]; results reproduced in S2A Fig). For further discussion, see S2 Text.

To model the data, we structured the model simulations to mirror the experimental design feature whereby one stimulus (“A,” corresponding e.g. to gratings presented on the left side of fixation) had constant stimulus strength S_A_ across all simulated trials, whereas the other stimulus (“B,” corresponding e.g. to gratings presented on the right side) could take on one of five possible values S_B,i_, with the intermediate value S_B,3_ defined to be equal to S_A_. The parameter controlling meta-d’ (τ) was fit to overall meta-d’ at the intermediate level of task performance where stimulus strength for A and B was equal and the empirical response-conditional meta-d’ curves intersected (Fig 2A). Full details of model fitting are provided in S1 Text.

In good agreement with the empirical data, simulated response-conditional meta-d’ curves for the Differential Tuned Inhibition model exhibit an X-shaped cross-over effect, such that meta-d’ for different response types either increases or decreases as d’ increases. The range of meta-d’ values exhibited by the model is both plausible and consistent with meta-d’ found in other empirical studies as well. Notably, since τ was fit only to overall meta-d’ at the single intermediate level of task performance, the meta-d’ curves at all other data points were not constrained by an explicit fitting procedure but rather arise naturally as a consequence of the single set of fitted parameters.

Importantly, as described in Methods (Sections 2.3 and 2.4), we also tested the importance of computing confidence from uninhibited evidence accumulation to the data fit by repeating the above-described simulation procedures, but this time computing simulated confidence from C_δ_ rather than C_x_, and using the fitted parameter τ_δ_ rather than τ. As expected, this alternative version of the model using C_δ_ was not able to capture, even qualitatively, the X-shaped cross-over dissociation in response-conditional meta-d’ observed in the empirical data from Maniscalco and colleagues [14] (Fig 2B).

Further simulation results for this experiment are presented in S2 Text. There we compare empirical response-conditional mean confidence for correct and incorrect responses to the values produced in the C_x_ and C_δ_ model simulations (S2 Fig), and show topographical plots of distributions of C_x_ and C_δ_ to provide further intuition for why the former, and not the latter, can capture the response-conditional meta-d’ dissociation (S3 Fig). We also point out conceptual and computational similarities between the C_x_ model and the two-dimensional signal detection theory (2D-SDT) model used by Maniscalco et al. [14] as a theoretical frame of reference for their experimental design and empirical findings, arguing that these similarities are precisely what allow the C_x_ model to capture the dissociation. Finally, in S3 Text and S4–S7 Figs, we demonstrate that a leaky competing accumulator (LCA) implementation [35] of the tuned inhibition model cannot capture the data as well as the model implementation explored in the main manuscript, and argue that the LCA model performs worse in part because it does not map as cleanly onto the 2D-SDT model structure as does the main model.

### 3.2 Simulating data from Koizumi, Maniscalco, & Lau 2015 [10] Experiment 1A

Koizumi, Maniscalco, & Lau [10] experimentally controlled stimulus properties to yield conditions where task performance (d’) was similar, yet mean confidence differed. In their Experiment 1A, subjects performed a grating tilt discrimination task. However, stimuli were actually composed of two superimposed gratings tilting in opposite directions, one with higher contrast (“Positive Evidence” or “PE” for short; the correct decision) and one with lower contrast (“Negative Evidence” or “NE”; the incorrect decision). Subjects had to indicate whether the higher contrast grating was tilting left or right and then rate confidence on a 4-point scale.

The key experimental manipulation was the introduction of High PE and Low PE conditions, in which the contrast of the NE gratings was set to 0.7*(PE grating contrast) and 0.35*(PE grating contrast), respectively, and PE grating contrast was controlled by thresholding procedures to achieve a criterion level of task performance (d’). Due to the distractor NE stimulus being stronger in the High PE condition, a higher level of PE was required to achieve a given level of d’, with the net effect that d’ was similar in Low PE and High PE conditions, whereas PE (and NE) were higher in the High PE condition than in the Low PE condition. Additionally, there were two levels of task difficulty at each level of PE, thus giving rise to four experimental conditions in all: “High PE, Easy,” High PE, Difficult,” “Low PE, Easy,” and Low PE, Difficult.” Koizumi et al found that the High PE and Low PE conditions yielded similar task performance (d’), but mean confidence was higher for High PE stimuli (Fig 1D of [10]).

Similarly to Maniscalco et al 2016 [14], the dissociation effect in Experiment 1A of Koizumi et al 2015 [10] is thought to arise from observers using decision-congruent evidence to assess confidence. Thus, we similarly expected that the dissociation between d’ and confidence would be captured well by simulations using C_x_ (corresponding to computing confidence from absolute accumulated decision-congruent evidence), but not by simulations using the alternative C_δ_ (corresponding to computing confidence from the ‘differencing’ units, i.e. the balance of evidence).

**Fig 3:**
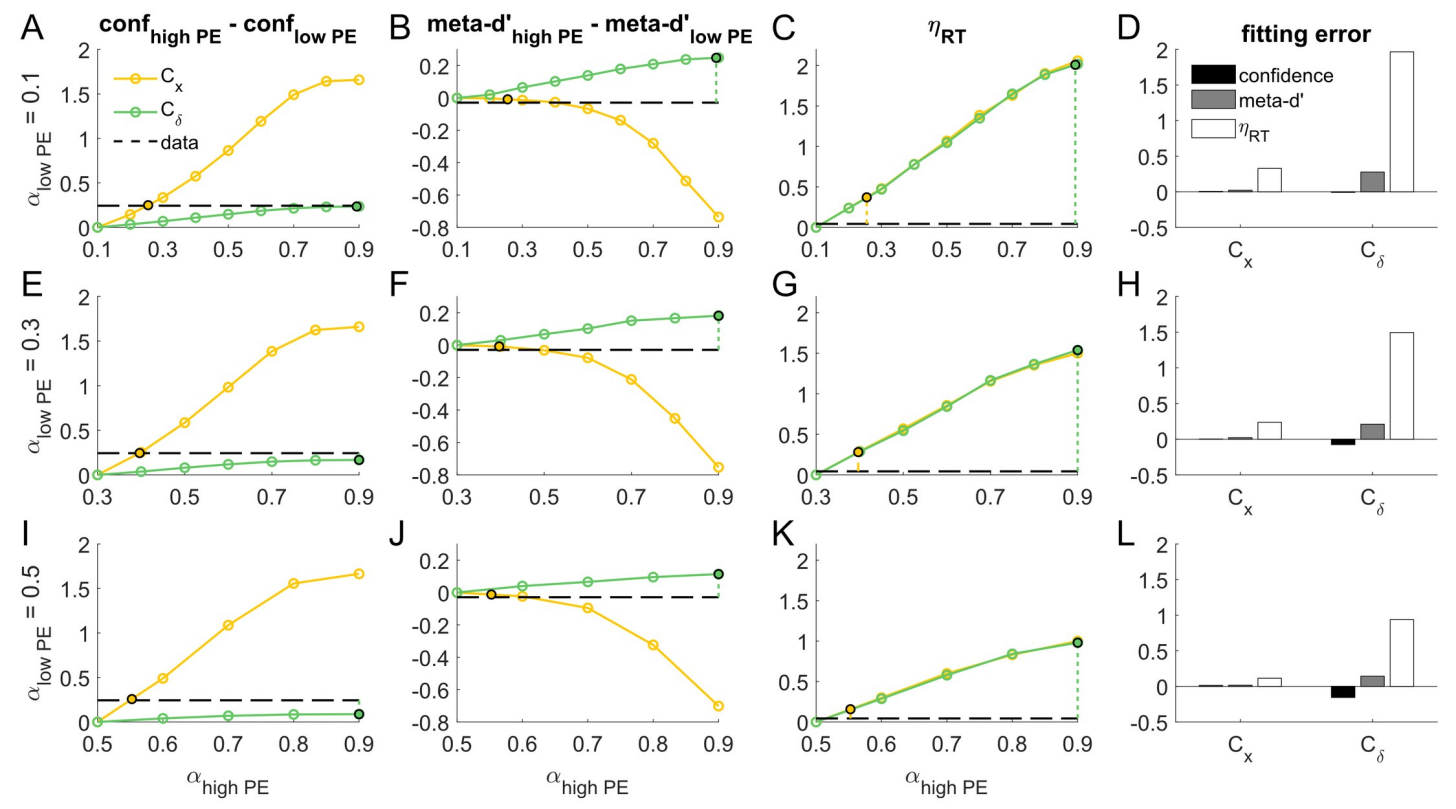
Comprehensive simulations showing fitting procedures for the main C_x_ model and alternative C_δ_ model for Koizumi et al.’s [10] Experiment 1A. Rows show simulation results for different settings of α_low PE_, which determines the relative strength of positive evidence (PE) and negative evidence (NE) in simulations for the Low PE condition of Koizumi et al., such that S_NE_ / S_PE_ = α_low PE_. Columns show performance-matched confidence differences (i.e. differences in confidence when d’ is exactly matched at the mean d’ value of the low PE condition), performance-matched meta-d’ differences, relative RT effect sizes (η_RT_), and model fitting error due to PE manipulations for Koizumi et al.’s Experiment 1A. For each of the C_x_ and C_δ_ models and each PE/NE balance in the Low PE condition (α_low PE_), we determined the level of NE in the High PE condition (α_high PE_) that best matched the observed magnitude of performance-matched confidence differences in the empirical data (dashed horizontal lines, column 1); these are marked with black circles. We then “read out” the predicted meta-d’ differences and relative RT effect sizes (columns 2 and 3, respectively) for each of these best-fitting PE/NE levels to get a comprehensive picture of the behavior of the system for both the C_x_ and C_δ_ models. Fitting error was computed for each model simply as the difference between the predicted value and the empirical value for confidence differences, meta-d’ differences, and relative RT effect sizes (column 4). Across all PE/NE balance levels tested, the C_x_ model produced comprehensively good fit to the empirical data from Koizumi et al.’s Experiment 1A; in contrast, the alternative C_δ_ model must be pushed to extremely high levels of α_high PE_ to capture the empirically observed confidence differences due to PE manipulations, leading to substantial errors in the corresponding fits for meta-d’ and RT.

To attain a comprehensive understanding of model behavior across all levels of low and high PE, we exhaustively mapped the behavior of the C_x_ and C_δ_ models across different levels of PE and NE, as follows. Since all stimuli in Koizumi et al. [10] Experiment 1A featured non-zero levels of positive and negative evidence, we modeled each condition using two stimulus drive parameters S_PE_ and S_NE_. In all simulations, we set a constraint such that S_NE_ = α S_PE_ for 0 < α < 1, and further constrained α to be lower in the Low PE condition, i.e. α_low PE_ < α_high PE_. We selected α_low PE_ = 0.1, 0.3, and 0.5 as three representative values of α to explore for the Low PE condition. For each level of α_low PE, i_, we explored a corresponding set of values of α_high PE, ij_ where the α_high PE, ij_ values were set as equally spaced values between α_low PE, i_ + 0.1 and 0.9, incrementing by steps of 0.1. For each pairing of α_low PE, i_ and α_high PE, ij_, we fit the model parameters to the average d’ and meta-d’ values in the Low PE, Easy and Low PE, Difficult conditions and observed how this influenced the difference in mean confidence between the simulated High PE and Low PE conditions (Fig 3A, 3E and 3I).

Simulations results revealed that the main C_x_ model can capture a wider range of differences in confidence due to manipulation of PE level, which allows it to capture the observed magnitude of the (High PE confidence–Low PE confidence) effects across all analyzed choices of α_low PE, i_ using relatively small increments in the corresponding α_high PE, ij_ (for α_low PE_ = 0.1, 0.3, and 0.5, the effect of PE level on confidence is captured by setting α_high PE_ = 0.2548, 0.3966, and 0.5525, respectively; Fig 3A, 3E and 3I). By contrast, the alternative C_δ_ model predicts only a very small influence of PE level on mean confidence; at α_low PE_ = 0.1 it can account for the confidence effect only by positing a massively stronger PE level at α_high PE_ = 0.8928 (Fig 3A), and when α_low PE_ > 0.1, no value of α_high PE_ ≤ 0.9 can account for the magnitude of the effect (Fig 3E–3I).

**Fig 4:**
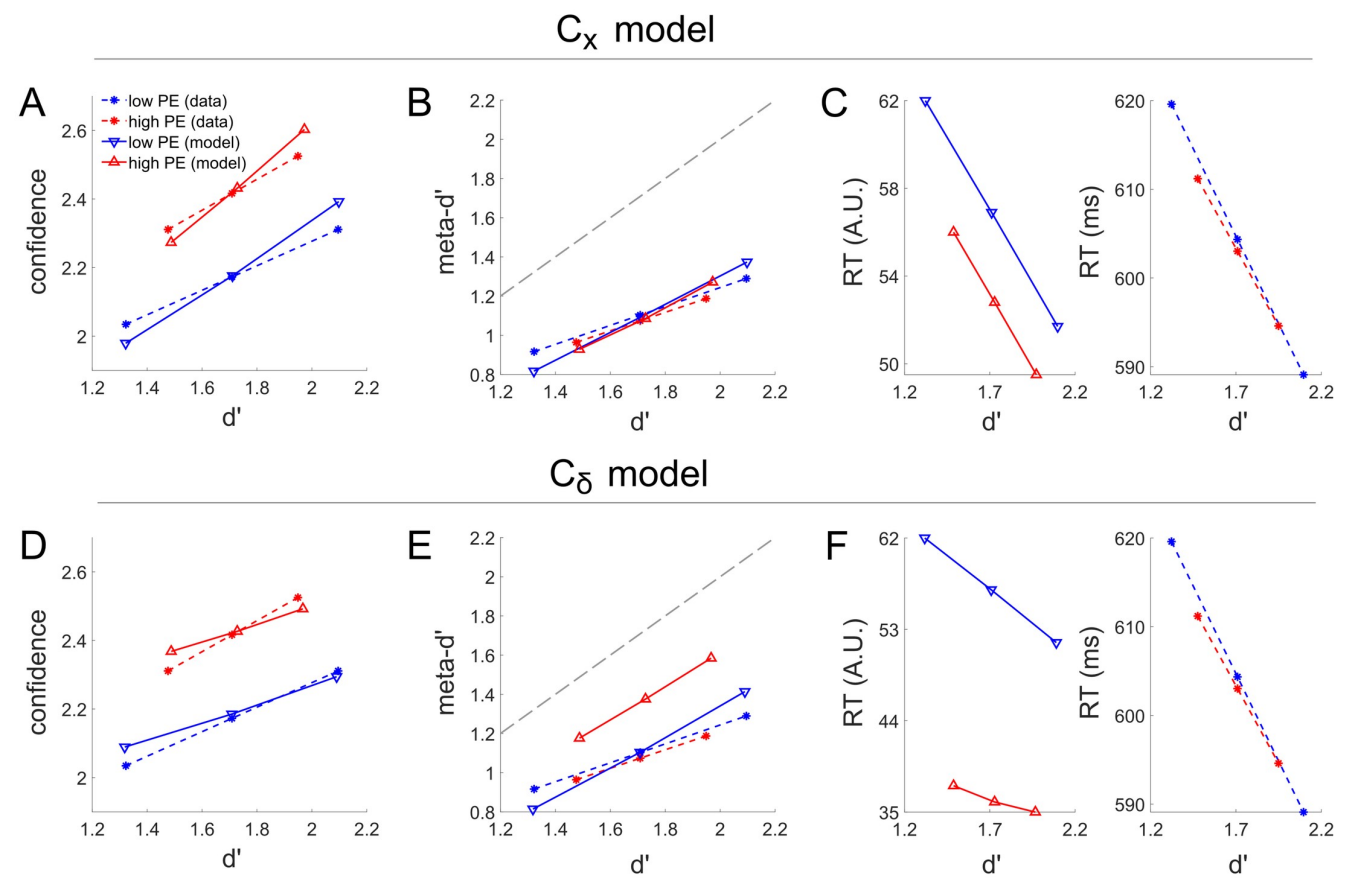
Modeling results for the effects reported by Koizumi and colleagues [10] in their Experiment 1A with α_low PE_ = 0.1 (corresponding to the first row of Fig 3) and parameter values chosen to best capture the effect of PE level on mean confidence (corresponding to the data points shown in black circles in Fig 3). The fitted main C_x_ model reproduces the differential confidence for matched d’ performance for high versus low PE stimuli (A), as well as the overlapping distributions of meta-d’ as a function of d’ (B). The main C_x_ model predicts a small different in reaction time as a function of PE (C) not seen in the empirical data, but the magnitude of the RT difference across PE levels is small relative to the magnitude of the RT difference across difficulty levels (higher d’ leads to much faster RT). In contrast, while the alternative C_δ_ model can reproduce the differential confidence as a function of PE level given matched d’ (D), it fails to capture the overlapping relationship between d’ and meta-d’ (E) and predicts overly large RT differences as a function of PE relative to the impact of d’ on RT (F). Thus, the main C_x_ model overall produces much closer fit to the data. Note that in the empirical data, there were only two levels of task difficulty; these correspond to the first and last data point in each linear curve in this plot. The second data point in each curve corresponds to the mean d’ across difficulty levels in the Low PE condition, which was used for model fitting purposes (see Methods for details).

To provide a more comprehensive characterization of the model fits to the empirical data, we also compared empirical and simulated data for the differences in reaction time and meta-d’ induced by the High PE and Low PE conditions. RT and meta-d’ data were not reported in Koizumi et al [10]; here we re-analyzed their data set and report the RT and meta-d’ data for the first time (Fig 4). In the empirical data, meta-d’ increased and RT decreased with increasing d’, but neither was appreciably modulated by PE level.

To assess the effect of PE level on simulated RT, we computed a relative RT effect size η_RT_, defined as

$$\eta_{RT}=({RT}_{high\;PE,med}-{RT}_{low\;PE,med})/({RT}_{low\;PE,easy}-{RT}_{low\;PE,difficult})$$ (8)

The subscripts “high PE, med” and “low PE, med” denote that these RT values were taken from simulations of the High PE and Low PE conditions, using S_low PE_ and S_high PE_ values chosen to match mean d’ across difficulty levels in the Low PE condition. The subscripts “low PE, easy” and “low PE, difficult” denote that these RT values were taken from the simulations of the Low PE condition, using S_low PE_ values chosen to match d’ in the Low PE, Easy and Low PE, Difficult conditions. Thus, η_RT_ measures simulated change in RT at an intermediate d’ value due to High vs Low PE (the numerator), relative to simulated change in RT in the Low PE condition due to Easy vs Difficult stimulus settings (the denominator).

Simulation results for (High PE meta-d’–Low PE meta-d’) and η_RT_ are shown in Fig 3B, 3F and 3J and Fig 3C, 3G and 3K, respectively. In general, the C_x_ and C_δ_ models make differing predictions about how PE level influences meta-d’, whereas they make the same prediction about how PE level influences RT (since perceptual decision making is driven by the same mechanism in both models). Of central interest for our purposes, however, is how these models predict meta-d’ and RT to behave at the specific values of α_low PE_ and α_high PE_ that yield differences in mean confidence matching those observed in the data. These points are marked by filled circles and vertical dashed lines in Fig 3. For purposes of comparison to simulated data, we used linear fits to interpolate the empirical meta-d’ and RT values at d’ = 1.71 (corresponding to the average d’ across the Low PE, Easy and Low PE, Difficult conditions) and computed the difference in the interpolated values for the High PE and Low PE conditions, yielding empirical values of High PE meta-d’–Low PE meta-d’ = -0.029 and η_RT_ = 0.044 (horizontal dashed lines in Fig 3).

At levels of α_low PE_ and α_high PE_ that yield a perfect fit to the effect of PE level on confidence, the main C_x_ model also exhibits an accurate fit to the effect of PE level on meta-d’, correctly predicting that PE level has a negligible effect on meta-d’ (PE effect on meta-d’ = -0.029 in the data; -0.008, -0.008, and -0.012 in the model for α_low PE_ = 0.1, 0.3, and 0.5 respectively). The C_x_ model incorrectly predicts that RT should be faster under High PE than Low PE, but the predicted magnitude of this effect is modest compared to the speed-up in RT due to Easy vs Difficult stimulus settings in the Low PE condition (η_RT_ = 0.044 in the data; 0.372, 0.281, 0.158 in the model for α_low PE_ = 0.1, 0.3, and 0.5 respectively). In the empirical data (RT_low PE, easy_—RT_low PE, difficult_) ≈ 30 ms, which can be used as a reference point for what the empirical and modeled η_RT_ effects translate to in terms of (RThigh PE, med—RT_low PE, med_). In the empirical data, η_RT_ = 0.044 corresponds to an effect of PE level on RT of approximately 0.044*30 ms = 1.3 ms, whereas the equivalent RT effects in the model would be 11.2, 8.4, and 4.7 ms for α_low PE_ = 0.1, 0.3, and 0.5 respectively. Thus, the magnitude of the incorrect prediction by the C_x_ model for the effect of PE level on RT ranges from about 3.5–10 ms. While it is notable that the model seemingly makes a qualitatively incorrect prediction here, the magnitude of the effect is small enough that it does not pose a prohibitive failure. It is also possible that the apparent lack of an RT effect in the data is a false negative, which could plausibly occur if the true effect size were 10 ms or less.

By contrast, at levels of α_low PE_ and α_high PE_ that yield a reasonably close fit to the effect of PE level on confidence, the alternative C_δ_ model fares considerably worse in its predictions for meta-d’ and RT. It incorrectly predicts that meta-d’ should be higher in the High PE condition (PE effect on meta-d’ = -0.029 in the data; 0.248, 0.181, and 0.114 in the model for α_low PE_ = 0.1, 0.3, and 0.5 respectively), and vastly overestimates the effect of PE level on RT (η_RT_ = 0.044 in the data; 2.010, 1.539, 0.980 in the model for α_low PE_ = 0.1, 0.3, and 0.5 respectively). By the same logic described above, these model predictions correspond to predicted RT effects due to PE level in the C_δ_ model of 60.3, 46.2, and 29.4 ms for α_low PE_ = 0.1, 0.3, and 0.5 respectively. This means the magnitude of the incorrect prediction by the C_δ_ model for the effect of PE level on RT ranges from 28 to 59 ms—errors 6–10 times larger than those of the C_x_ model. Such large effect sizes are also statistically incompatible with observing an effect close to zero in a reasonably powered sample (Koizumi et al’s data set consisted of 480 trials). Side-by-side comparisons of the fitting errors for the C_x_ and C_δ_ models at the values of α_high PE_ yielding the best fit the effect of PE level on mean confidence are shown in Fig 3D, 3H and 3L.

To further illustrate model behavior, in Fig 4 we show fits to the mean confidence, meta-d’, and RT data for the full PE level (High / Low) x task difficulty (Difficult / Easy) design, separately for the C_x_ and C_δ_ models. Displayed fits are derived from simulations using α_low PE_ = 0.1 (the only level of α_low PE_ for which the alternative C_δ_ model could capture the effect of PE level on confidence within the explored range of α_high PE_ levels) and α_high PE_ = 0.2548 (C_x_ model) or α_high PE_ = 0.8928 (C_δ_ model), with these α_high PE_ values chosen so as to best fit the effect of PE level on mean confidence (corresponding to black circles in Fig 3). Echoing the more general results of Fig 3, the results of Fig 4 demonstrate how although both models can achieve good fits to the effect of PE level on performance-matched confidence, the C_x_ model is considerably more accurate in its corresponding predictions for meta-d’ and RT. As a reminder, these differences between the C_x_ and C_δ_ models regarding the effect of PE level on meta-d’ and RT stem from the differences between these models in fitted stimulus drive S_high PE_ and S_high NE_ (controlled by α_high PE_ via the equation S_high NE_ = α_high PE_ * S_high PE_) needed to account for the main d’ and confidence effects.

In summary, these simulations demonstrate that across a comprehensive range of PE and NE levels, the C_x_ model provides the best overall account of the data, achieving a fit to the effect of PE level on confidence that also yields an accurate fit to the effect of PE level on meta-d’, and a reasonable fit to the effect of PE level on RT. Furthermore, these fits are accomplished with reasonably small differences in PE levels (i.e. with α_high PE_ reasonably close to α_low PE_). By contrast, although the C_δ_ model can also achieve a close fit to the effect of PE level on confidence, its corresponding fits to the effect of PE level on meta-d’ and especially RT are poor, and furthermore this fit can only be achieved by positing an implausibly large difference in PE level.

### 3.3 Simulating data from Koizumi, Maniscalco, & Lau 2015 [10] Experiment 2B

In their Experiment 2B, Koizumi et al [10] used an alternative method to that used in Experiment 1A to achieve performance-matched differences in confidence. Stimuli consisted of random dot kinematograms with a fraction of the dots moving coherently to the left or right on each trial. Subjects had to indicate motion direction and then rate confidence on a 4-point scale. “Positive Evidence” (PE) was defined as the number of coherently moving dots, with this quantity depending on both the fraction of coherently moving dots and dot density; the remaining, randomly moving dots constituted sensory noise.

It is important to note a disanalogy between the structure of Experiments 1A and 2B of Koizumi et al. In Experiment 1A, stimuli consisted of the superposition of Positive Evidence (a high contrast grating tilting left or right), Negative Evidence (a lower contrast grating tilted in the opposite direction), and noise (randomly chosen fluctuations for pixel intensities). The PE component provided perceptual evidence for the correct perceptual choice, the NE component provided perceptual evidence for the incorrect perceptual choice, and noise systematically favored neither choice. By contrast, in Experiment 2B, stimuli consisted only of PE (coherent leftward or rightward motion) and noise (random motion). The analogue of NE in this task would be weaker coherent motion in the direction opposite to the PE motion, but no such opposite coherent motion was present in these stimuli. (To preempt a possible confusion, we note that Koizumi et al. used the term “NE” to refer to the randomly moving dots in Experiment 2B; here we have decided to abandon this usage in favor of reserving the term “NE” specifically for systematic perceptual evidence for the incorrect perceptual choice.)

The key experimental manipulation in Experiment 2B was the introduction of High PE and Low PE conditions; the fraction of coherently moving dots was equal across conditions, but dot density was twice as high in the High PE condition, thus yielding a higher absolute number of coherently moving dots. Additionally, there were two levels of task difficulty at each level of PE, thus giving rise to four experimental conditions in all: “High PE, Easy,” High PE, Difficult,” “Low PE, Easy,” and Low PE, Difficult.” Koizumi et al [10] found that the High PE and Low PE conditions yielded similar task performance (d’), but mean confidence was higher for High PE stimuli (Fig 4A of [10]).

As with the previous data sets, we predicted that the dissociation between d’ and confidence in this experiment would be better captured by the main C_x_ model than the alternative C_δ_ model, and tested this prediction by comparing model fits to the data. However, unlike the experimental results discussed above (Koizumi et al.’s [10] Experiment 1A), in this case the effect of performance-matched confidence was not driven by PE / NE manipulations; coherent motion always occurred in one direction only (PE), without any simultaneous coherent motion in the opposite direction (NE). Thus, it was necessary to use an alternative modeling strategy to the one used to model Koizumi et al.’s [10] Experiment 1A.

In the High PE condition of Experiment 2B, d’ was similar to the Low PE condition due to having the same fraction of coherently moving dots, whereas confidence was higher due to having a higher absolute number of coherently moving dots. Thus, we reasoned that these data could be well modeled by supposing that the High PE condition has both stronger stimulus drive (more coherently moving dots) and stronger noise (more randomly moving dots); the increase in both stimulus strength and noise allows for signal-to-noise ratio (and hence d’) to be similar, even as the same increases in signal and noise lead to higher absolute values of sensory evidence (and hence higher confidence). Indeed, previous work has demonstrated that increases in signal strength and noise can yield performance-matched increases in confidence under the assumption of fixed decision criteria [38], an assumption that is supported empirically in cases where the experimental conditions modeled with lower and higher variance in sensory evidence are randomly interleaved across trials [17]. We implemented this idea in the modeling by positing that the parameter controlling standard deviation of instantaneous sensory evidence, σ, would take on a larger value in the High PE condition.

To fit the C_x_ and C_δ_ models to the data of Koizumi et al.’s [10] Experiment 2B, we adopted a similar model fitting approach as described above for their Experiment 1A, as detailed below.

**Fig 5:**
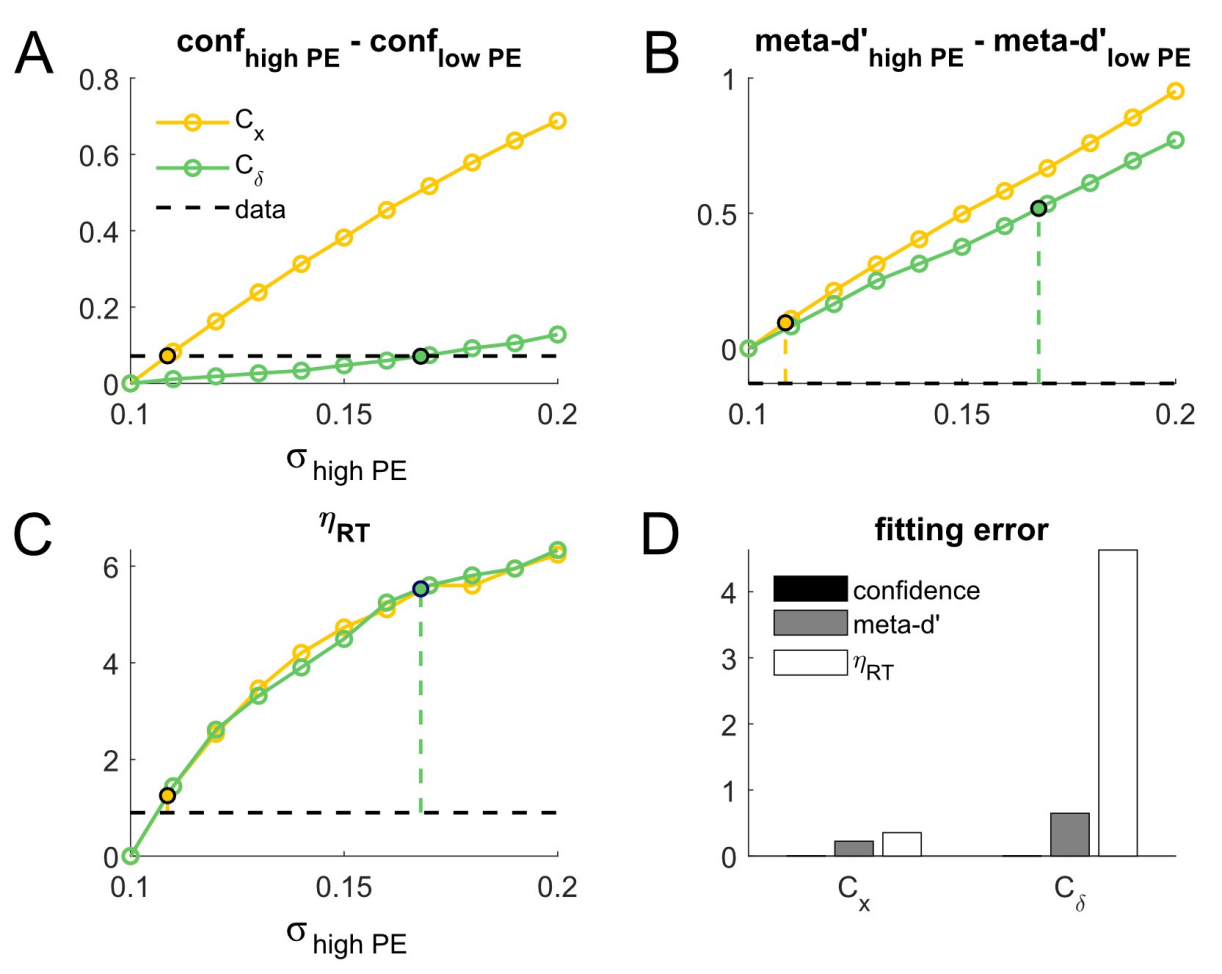
Comprehensive simulations showing fitting procedures for the main C_x_ model and alternative C_δ_ model for Koizumi et al.’s [10] Experiment 2B. As in the analysis of Koizumi et al.’s Experiment 1A in Fig 3, we examined performance-matched confidence differences, performance-matched meta-d’ differences, and relative RT effect sizes as a function of PE level. (A) For each of the C_x_ and C_δ_ models, we determined the level of sensory evidence noise in the High PE (σ_high PE_) condition that best matched the observed magnitude of confidence differences in the empirical data (dashed horizontal line), marked with black circles. We then “read out” the predicted meta-d’ differences (B) and relative RT effect sizes (C). (D) We also computed fitting error for each model as the difference between the simulated and empirical confidence differences, meta-d’ differences, and relative RT effects (D). As with Experiment 1A, the C_x_ model produced a good fit to the empirical data across the entire spectrum of measures considered, whereas the alternative C_δ_ model produced substantial errors for predicted meta-d’ and RT when made to match the empirically-observed confidence differences.

To attain a comprehensive understanding of model behavior across different levels of sensory evidence variability, we fixed σ_low PE_ = 0.1 in the Low PE condition (the same value for σ used in all simulations described above) and explored model behavior when using values of σ_high PE_ = 0.11, 0.12, …, 0.2 in the High PE condition (i.e. ranging from 10% to 100% increases in σ). For each pairing of σ_low PE_ and σ_high PE_, we fit the model parameters to the average d’ and meta-d’ values in the Low PE, Easy and Low PE, Difficult conditions and observed how this influenced the difference in mean confidence between the simulated High PE and Low PE conditions (Fig 5A).

Simulations results revealed that the main C_x_ model can capture a wider range of differences in confidence due to manipulation of σ, which allows it to capture the observed magnitude of the (High PE confidence–Low PE confidence) effects using a relatively small increment in the corresponding σ_high PE_ (for σ_low PE_ = 0.1, the effect of PE level on confidence is captured by setting σ_high PE_ = 0.1087; Fig 5A). By contrast, the alternative C_δ_ model predicts only a comparatively small influence of σ on mean confidence, requiring a value of σ_high PE_ = 0.1679 to account for the confidence (Fig 5A). Thus, to achieve the same effect on performance-matched confidence, the C_x_ model posits only a ~9% increase in sensory noise, whereas the C_δ_ model requires a much more substantial ~68% increase in noise.

**Fig 6:**
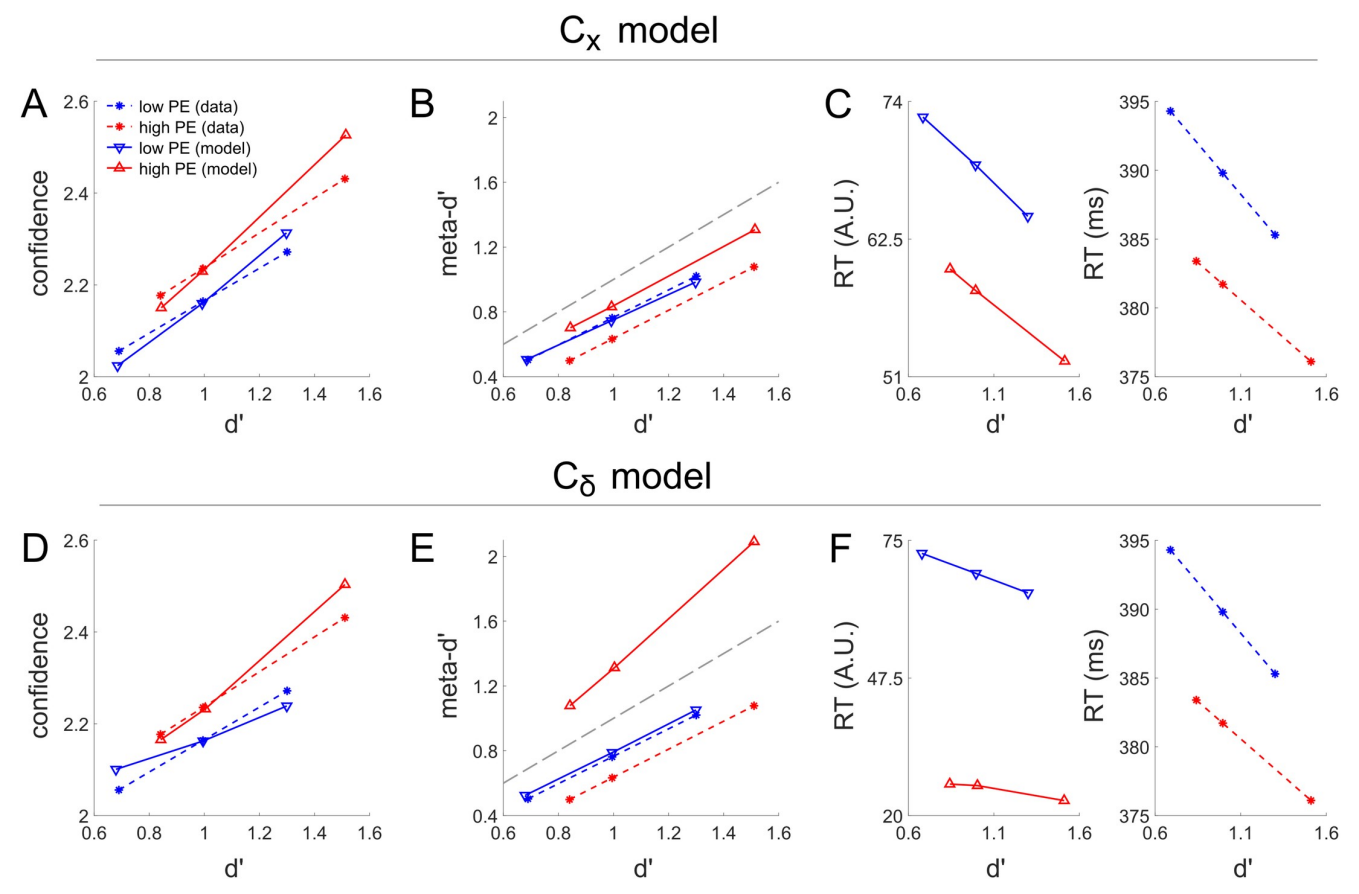
Modeling results for the effects reported by Koizumi and colleagues [10] in their Experiment 2B with parameter values chosen to best capture the effect of PE level on mean confidence (corresponding to the data points shown in black circles in Fig 5). As for their Experiment 1A, the fitted main C_x_ model reproduces the differential confidence for matched d’ performance for high versus low PE stimuli (A) and the relatively overlapping distributions of meta-d’ as a function of d’ (B), although somewhat less well than for experiment 1. However, in contrast to Experiment 1A the main C_x_ model now correctly predicts the relationship between RT differences a s function of d’ and PE (C) much better than for Experiment 1A. In contrast, while the alternative C_δ_ model can reproduce the differential confidence as a function of density level given matched d’ (D), it again badly fails to capture the overlapping relationship between d’ and meta-d’ (E)–this time predicting an implausibly high meta-d’ value. The alternative C_δ_ model also again predicts overly large RT differences as a function of density level, while predicting almost zero impact of d’ on RT (F). Thus, as with Experiment 1A, the main C_x_ model overall produces much closer fit to the data. Note that in the empirical data, there were only two levels of task difficulty; these correspond to the first and last data point in each linear curve in this plot. The second data point in each curve corresponds to the mean d’ across difficulty levels in the Low PE condition, which was used for model fitting purposes (see Methods for details).

To provide a more comprehensive characterization of the model fits to the empirical data, we also compared empirical and simulated data for the differences in reaction time and meta-d’ induced by the High PE and Low PE conditions. RT and meta-d’ data were not reported in Koizumi et al. [10]; here we re-analyzed their data set and report the RT and meta-d’ data for the first time (Fig 6). In the empirical data, meta-d’ increased and RT decreased with increasing d’. Additionally, PE level appeared to have a modest effect on both meta-d’ and RT, with the high PE condition having slightly lower meta-d’ and faster RT.

Simulation results for (High PE meta-d’–Low PE meta-d’) and η_RT_ are shown in Fig 5B and 5C, respectively. As before, the point of main interest is how these models predict meta-d’ and RT to behave at the specific values of σ_high PE_ that yield differences in mean confidence matching those observed in the data. These points are marked by filled circles and vertical dashed lines in Fig 5. For purposes of comparison to simulated data, we used linear fits to interpolate the empirical meta-d’ and RT values at d’ = 0.99 (corresponding to the average d’ across the Low PE, Easy and Low PE, Difficult conditions) and computed the difference in the interpolated values for the High PE and Low PE conditions, yielding empirical values of High PE meta-d’–Low PE meta-d’ = -0.129 and η_RT_ = 0.898 (horizontal dashed lines in Fig 5).

At the level of σ_high PE_ that yields a perfect fit to the effect of PE level on confidence (black circles in Fig 5A), the main C_x_ model incorrectly predicts that meta-d’ should be higher under High PE, although the magnitude of this incorrect prediction is modest (PE effect on meta-d’ = -0.129 in the data; 0.095 in the model). Conversely, the C_x_ model correctly predicts that RT should be faster under High PE than Low PE, and the predicted magnitude of this effect is comparable to the empirically observed effect (η_RT_ = 0.898 in the data; 1.252 in the model).

In the empirical data (RT_low PE, easy_—RT_low PE, difficult_) ≈ 10 ms, which can be used as a reference point for what the empirical and modeled η_RT_ effects translate to in terms of (RT_high PE, med_—RT_low PE, med_). In the empirical data, η_RT_ = 0.898 corresponds to an effect of PE on RT of approximately 0.898*10 ms = 8.98 ms, whereas the equivalent RT effect in the model would be 12.52 ms. Thus, the error in the the C_x_ model’s prediction for the effect of PE level on RT amounts to about 3.5 ms.

By contrast, at the level of σ_high PE_ that yield a perfect fit to the effect of PE level on confidence, the alternative C_δ_ model fares considerably worse in its predictions for both meta-d’ and RT. Like the C_x_ model, the C_δ_ model incorrectly predicts that meta-d’ should be higher in the High PE condition, but the magnitude of this incorrect prediction is 5 times as large as in the C_x_ model (PE effect on meta-d’ = -0.129 in the data; 0.518 in the model); indeed, the predicted meta-d’ in the High PE condition is considerably higher than d’, which violates a theoretically expected, and commonly empirically observed, constraint such that meta-d’ ≤ d’ [29]. Similarly, the C_δ_ model vastly overestimates the effect of PE level on RT (η_RT_ = 0.898 in the data; 5.530 in the model). By the same logic described above, this η_RT_ value corresponds to a predicted effect of PE level on RT of 55.3 ms. Thus, the magnitude of the incorrect prediction by the C_δ_ model for the effect of PE level on RT is about 45 ms—an error over 10 times larger than that of the C_x_ model (3.5 ms). Side-by-side comparisons of the fitting errors for the C_x_ and C_δ_ models at the values of σ_high PE_ yielding the best fit the effect of PE level on mean confidence are shown in Fig 5D.

To further illustrate model behavior, in Fig 6 we show fits to the mean confidence, meta-d’, and RT data for the full PE level (High / Low) x task difficulty (Difficult / Easy) design, separately for the C_x_ and C_δ_ models. Displayed fits are derived from simulations using σ_low PE_ = 0.1 and σ_high PE_ = 0.1087 (C_x_ model) or σ_high PE_ = 0.1679 (C_δ_ model), with these σ_high PE_ values chosen so as to best fit the effect of PE level on mean confidence (corresponding to black circles in Fig 5). Echoing the more general results of Fig 5, the results of Fig 6 demonstrate how although both models can achieve good fits to the effect of PE level on performance-matched confidence, the main C_x_ model is considerably more accurate in its corresponding predictions for meta-d’ and RT. Here, as above, differences in meta-d’ and RT between the C_x_ and C_δ_ models result from differences in fitted stimulus drive S_PE_ for each model, which was needed to account for the main d’ and confidence effects.

In summary, these simulations demonstrate that across a comprehensive range of σ levels, the main C_x_ model provides the best overall account of the data, achieving a fit to the effect of PE level on confidence that also yields a reasonably close fit to the effect of PE level on meta-d’ (albeit one that goes in the wrong direction), as well as a close fit to the effect of PE level on RT. Furthermore, these fits are accomplished with a small (~9%), and therefore plausible, increase in σ. By contrast, although the alternative C_δ_ model can also achieve a close fit to the effect of PE level on confidence, its corresponding fits to the effect of PE level on meta-d’ and RT are both very poor, and its prediction for meta-d’ in the High PE condition is implausibly high. Furthermore, the fit to the confidence effect can only be achieved by positing a relatively large increase (~68%) in σ.

## 4. Discussion

How the brain calculates subjective decision confidence is still a topic of active debate [1,2,39–43]. Although dominant models suggest that confidence reflects an optimal readout of the probability that a decision is correct [1–9], it appears challenging for such models to account for counterintuitive behaviors in which confidence and accuracy dissociate [10,11,13,14,16,17,44]. An alternative hypothesis suggesting that confidence reflects a heuristic reliance on decision-congruent evidence [10,14,16,18,21] captures many of these behaviors, and is supported by human intracranial electrophysiology [23].

Here, we considered how decision-congruent computations of perceptual confidence might be biologically implemented based on known properties of perceptual circuitry. We hypothesized that tuned inhibition (a generalization of the concept of tuned normalization) [24–26] differentially influences a neuron’s role in perceptual decision-making and confidence, such that more inhibited units (corresponding to the net evidence for a perceptual choice) drive decisions and less inhibited units (corresponding to decision-congruent evidence) drive confidence. We developed the Differential Tuned Inhibition model to test this hypothesis. Our results show that such a network can explain counterintuitive behaviors reported in the literature [4,10,12–14,16–18,44,45]. We further demonstrate that the model’s special property of weighting less inhibited units more heavily in computing confidence is the key to capturing empirical findings, since control simulations demonstrate that the model fails to reproduce these findings when instead *more* inhibited units drive confidence. This provides preliminary but converging evidence that decision-congruent confidence computations may be implemented via tuned inhibition.

It might be argued that some over-simplified optimal diffusion-type models [6,7,9] should not be expected to account for counterintuitive behaviors due to their simplicity. A recent modification of these optimal diffusion-type models suggests that the optimal perceptual confidence readout must also depend on the time it took for evidence to accumulate [3–5]. Although it has been suggested that neurons in lateral intraparietal cortex (LIP) may encode elapsed time [46–48], these neurons’ activity has not yet been causally or directly linked to subjective confidence (see also [49]). This suggests that how this time-dependent diffusion framework might be biologically implemented is nontrivial, inspiring the work presented here.

We employed a two-stage evidence accumulation model [9] in which, after the initial perceptual decision is triggered by accumulated evidence surpassing the decision threshold, accumulation continues for τ additional time steps. As τ increases, confidence ratings become more diagnostic of task accuracy, and thus meta-d’ increases (S1C and S1D Fig). This model structure thus has the considerable benefit of naturally lending itself to fitting meta-d’, which (in our experience) sometimes proves a difficult task for dynamic evidence accumulation models.

We adopted a simple but powerful approach for model fitting and model comparison. After fixing sensory noise σ and decision threshold T to *a priori* values, stimulus drive S could be chosen to exactly match all empirical d’ values, and (given the fitted value of τ), confidence thresholds U_r_ could be chosen to exactly match overall empirical distributions of confidence ratings (i.e. P(conf = i) where 1 ≤ i ≤ 4 for a 4-point rating scale). Against this backdrop (which already perfectly captures much of the data), our general strategy was to fit τ to the meta-d’ value at a single data point and, using this fitted value of τ in all other conditions, observe what patterns emerged in the confidence data across all other data points as a consequence of the structure of the simulation (i.e., the differing stimulus strengths for the “B” stimulus in the simulations of Maniscalco et al [14]; the differing levels of positive and negative evidence in the simulations of Koizumi et al [10] Experiment 1A; the differing levels of sensory evidence noise in the simulations of Koizumi et al [10] Experiment 2B). Our key emphasis was thus not to derive exact fits for every data point, but rather to assess qualitative patterns in the fits that emerged from a simple modeling approach and compare how the fits differed for the main Differential Tuned Inhibition model (i.e. the C_x_ model) and the alternative C_δ_ model.

The counterintuitive empirical findings of Maniscalco et al [14], which show that in some conditions metacognitive sensitivity can decrease in spite of increasing task performance, provide a strong test for models of perceptual confidence, since such models usually naturally predict that metacognitive sensitivity and task performance positively correlate (and indeed, such positive correlations are widely observed in empirical data). We found that simulations of this data set unambiguously favored the main C_x_ model over the alternative C_δ_ model, as only the former was capable of reproducing the distinctive X-shaped dissociation in response-conditional meta-d’ curves, whereby meta-d’ for “S1” responses decreases and meta-d’ for “S2” responses increases with increases in d’ (Fig 2).

In the same paper as modeled here, Maniscalco and colleagues [14] also examined the effects of feedback on metacognitive sensitivity (meta-d’). They found that feedback on task accuracy and confidence judgments led to the near-disappearance of the X-shaped crossover effect in response-conditional meta-d’, instead producing results resembling those of the C_δ_ model presented here (our Fig 2B, their Fig 7). These observations suggest that decision-congruent evidence biases in perceptual confidence can change fluidly depending on training or other factors, suggesting promising avenues for future studies testing the Differential Tuned Inhibition model with paradigms designed to manipulate response-conditional meta-d’. We note that our model formulation does not explicitly specify whether the confidence readout mechanism may be innate or learned through the lifetime, and therefore is not committed to viewing the readout as inflexible or hardwired versus the possibility of accommodating changes due to learning or other factors. Future research could investigate an expanded version of the model in which the confidence readout mechanism, and its potential change as a function of learning, is explicitly modeled, as well as exploring whether it may rest on innate versus learned mechanisms.

At first glance, the simulation results for Koizumi et al [10] Experiments 1A and 2B were more equivocal, since both the C_x_ and C_δ_ models could capture the dissociations in these data whereby some conditions exhibit different levels of mean confidence in spite of having similar task performance (Figs 4A, 4D, 6A and 6D). However, the alternative C_δ_ model required implausible stimulus manipulations many times stronger than those required by the main C_x_ model to capture the performance-matched confidence effects. Furthermore, the parameter settings that allowed the C_δ_ model to achieve this fit also entailed predicted effects of reaction time and meta-d’ that were strongly incorrect, with error magnitudes many times as large as the corresponding predictions for the C_x_ model (Figs 3–6), including an implausibly large predicted value for meta-d’ such that meta-d’ > d’ (Fig 6E). By contrast, the C_x_ model fits to meta-d’ and RT, while not perfect, were all reasonably close to the empirically observed data—a finding made more impressive by the fact that the model structure and parameter values were not chosen in any way so as to provide reasonable fits to meta-d’ and RT, but rather these fits naturally “fell out” of the simulation structure intended to match salient features of experimental design and parameter values intended to fit the d’ and mean confidence data. Thus, on balance, the results of the Koizumi et al [10] simulations strongly favored the main C_x_ model.

Thus, we found that by considering the models’ ability to capture a comprehensive set of behavioral data—including d’, RT, mean confidence, and meta-d’—we were better able to distinguish the effectiveness of competing models in capturing those data. We especially wish to highlight the utility and power of metacognitive sensitivity (as measured here by meta-d’ [29]) as a target for model fitting. In addition to capturing task performance, reaction time, or mean confidence within an aggregate of trials, any successful model of perceptual confidence should also be able to account for metacognitive sensitivity—i.e., the trial-by-trial correspondence between confidence and accuracy. As we have shown here, assessing fit to metacognitive sensitivity data can be an incisive tool for model evaluation and comparison—yet the vast majority of extant studies on dynamic decision making models of perceptual confidence do not consider patterns of metacognitive sensitivity in the data or how the model can (or cannot) account for them. The simulations of the Koizumi et al [10] experiments in the present study are instructive in that the competing models appeared equal in their ability to account for performance-matched confidence, but yet could be distinguished by their relative ability to account for meta-d’.

Notably, although we only performed model simulations for three sets of experimental results, the simulation findings apply more broadly to any experiments using similar manipulations. Several other studies have used manipulations of positive and negative evidence to achieve performance-matched confidence of the sort employed by Koizumi et al [10] Experiment 1A [15,16,45]. The simulations of Koizumi et al [10] Experiment 2B are perhaps even more broadly informative, insofar as they may shed light on any experimental design that can be modeled as influencing task performance and confidence by means of altering the variability of sensory evidence—including studies employing stimulus manipulations [5] but also manipulations of attention [13] and direct intervention on neural activity [11,12]. A recent model with similar flavor to ours proposed a competing accumulator framework in which both confidence and decision were read out from the same units, but these units were only partially inhibited [21]; this model captured effects wherein confidence appeared to rely more on decision-congruent evidence, but decisions on a balance of evidence between decision-congruent and decision-incongruent evidence. However, in that paper the authors did not explore whether such a model could capture meta-d’—and as we have shown here the summary behaviors of d’ and confidence can be explained by an alternative model in which the same units drive decisions and confidence but, critically, meta-d’ could not be captured by such a model. Future work should explore the degree to which strength of feed-forward inhibition might produce more nuanced behaviors, as our goal with the current model was to provide a proof of concept that tuned inhibition in sensory circuits can provide a biologically plausible mechanism for implementing decision-congruent confidence computations.

Our results suggest a potential adaptive consequence for the presence of tuned inhibition [24–26] within a behaving organism: the presence of both more and less inhibited neurons within a perceptual decision-making circuit may allow an organism to better solve both fine-grained discrimination and detection tasks. When making fine-grained discrimination or identification judgments about an object or stimulus (“What is that thing?”), a useful strategy would be to rely on a system that is not as sensitive to random fluctuations, i.e. a more strongly inhibited system. But when making detection decisions (“Is there something out there?”), such strong inhibition would be highly undesirable, so a useful strategy would be to rely on less inhibited parts of the network. Both of these tasks are important for an organism to execute, and so it seems beneficial that a system might retain elements that can preferentially contribute to each task rather than implementing only one.

The question then becomes why the system would recruit the ‘detection’ portions of its circuitry to compute confidence, specifically relying on the magnitude of decision-congruent evidence. One reason may simply be heuristic, that the detectability and identifiability of a stimulus are often correlated in the real world; although in laboratory conditions these can certainly be dissociated, in real-world conditions they often go hand in hand. Indeed, it has been noted that the width of the posterior distribution in a probabilistic population code [42,50] covaries with the overall firing rate of a population [51]; less inhibited ‘detection’ neurons would more readily affect a population’s overall firing rate, suggesting a potential neural substrate for this heuristic. Perhaps due to this strong statistical coincidence in naturalistic environments, the system did not need to evolve away from such a heuristic, which also conveniently minimizes the need to retain information about unchosen stimulus identity possibilities once a perceptual inference has been made [23] and therefore might be computationally efficient. Indeed, such over-reliance on decision-congruent evidence—i.e., a “confirmation bias” [52]—has also been observed in other post-decisional (non-metacognitive) perceptual judgments [19,20], value judgments [53–58], and metamemory [59,60], suggesting it may be a domain-general strategy that serves also to reduce cognitive dissonance and improve self-consistency.

Using the absolute strength of decision-congruent evidence to judge confidence could also indicate that a confidence judgment attempts to infer the possible *cause* of the signals that led to the perceptual inference as externally- or internally-generated [61–63]: Are these signals strong enough to indicate an external stimulus, or are they likely to simply reflect internal noise fluctuations? A mechanism that keeps track of the absolute amount of evidence, regardless of the balance, would be critical to successfully solving such a causal inference problem by allowing the system to differentiate between strong versus weak signals even when the signals themselves are equally ambiguous (i.e., equally favor one versus another possible stimulus identity). And finally, that ‘detection’ circuitry might contribute to metacognitive judgments is also supported by reports of neurons coding for the detectability (or lack thereof) of a stimulus in prefrontal cortex [64], an area known to be involved in metacognitive computations (including judgments of ‘visibility, i.e. awareness) in perception and memory [39,65–75].

Because the above results are suggestive in nature, confirmation that tuned inhibition is utilized in perceptual decisions versus confidence judgments as hypothesized here will critically depend on experiments designed to reveal the biological mechanism in awake, behaving animals. As an initial exploratory test, however, we capitalized on an existing dataset consisting of electrophysiological recordings in Rhesus macaque superior colliculus, a subcortical area involved in perceptual decision-making [76–79] and containing the type of evidence accumulation neurons typically assumed to be involved in perceptual decision-making [3,6,7,9,35,40,80] (S4 Text); previously, tuned inhibition (tuned normalization) has only been reported in areas not involved in evidence accumulation [24–26]. Using catch trials from those data, we see preliminary support for one of our model’s critical predictions: that evidence accumulation neurons in perceptual decision-making areas ought also to exhibit tuned inhibition. Additional detail is provided in S4 Text and S8 Fig. Future work should extend these preliminary analyses and design specific experiments to test and arbitrate among our model and competing models of perceptual confidence [3–9].

Here we demonstrated that inhibition tuning provides a biologically plausible mechanism for implementing confidence computations that demonstrate an over-reliance on decision-congruent information. Our findings lead to testable hypotheses about the role of tuned inhibition in a neuron’s contribution to a decision versus a confidence judgment: activity of more inhibited units should reflect an observer’s objective decisions more than confidence judgments, while the opposite should be true for less inhibited neurons. Future electrophysiological studies should further explore the extent to which this hypothesis can be verified in the neurobiology of perceptual decision-making circuitry. It has also been reported that tuned inhibition is spatially ‘clumped’, i.e. that nearby neurons have more similar inhibition profiles than neurons separated by longer distances [25]. The present findings thus pave the way for noninvasive neuroscience techniques, such as spatially coarser functional MRI in humans, to clarify the role of inhibition tuning in perceptual and cognitive decisions and metacognitive evaluations of these choices.

## Supporting information

S1 Text Full methods for model fitting. (DOCX)

S2 Text Additional simulation results and comparison of the C_x_ model to the 2D-SDT model of Maniscalco, Peters, & Lau 2016. (DOCX)

S3 Text Testing a Leaky Competing Accumulator implementation of the model on the data of Maniscalco, Peters, & Lau 2016. (DOCX)

S4 Text Evidence for tuned inhibition in macaque superior colliculus. (DOCX)

S1 Fig Parameter fitting for the simulations of Maniscalco, Peters, & Lau 2016. (TIF)

S2 Fig Data and simulations for mean confidence as a function of S2 stimulus strength, perceptual decision, and accuracy. (TIF)

S3 Fig Simulated distributions of evidence in confidence units at the time of confidence rating as a function of stimulus and accuracy. (TIF)

S4 Fig LCA model structure. (TIF)

S5 Fig Simulation results for the model shown in S4A Fig. (TIF)

S6 Fig Simulated distributions of evidence in confidence units at the time of confidence rating as a function of stimulus and accuracy for the LCA model shown in S4A Fig. (TIF)

S7 Fig Simulation results for the model shown in S4B Fig. (TIF)

S8 Fig Evidence for tuned inhibition in macaque superior colliculus (SC). (TIF)
