# Supplementary material for: Tuned inhibition in perceptual decision-making circuits can explain seemingly suboptimal confidence behavior
Source: PLoS Comput Biol. 2021 Mar 29;17(3):e1008779. doi: 10.1371/journal.pcbi.1008779 (PMC8032199; doi:10.1371/journal.pcbi.1008779)
Supplement: S1 Text — (DOCX) [file pcbi.1008779.s001.docx]

Tuned inhibition in perceptual decision-making circuits can explain seemingly suboptimal confidence behavior

Authors: Brian Maniscalco, Brian Odegaard, Piercesare Grimaldi, Seong Hah Cho, Michele A. Basso, Hakwan Lau, & Megan A. K. Peters

**S1 Text: Full methods for model fitting**

**S1.1. Simulations for Maniscalco, Peters, & Lau 2016** [1]

S1.1.1 Selecting values for σ and T

First, we arbitrarily set accumulation noise σ = 0.1. The choice of this parameter value is arbitrary since, if no parameter values are fixed, identical simulation results can be obtained by a simple scaling of the model parameters. We then set decision threshold T = 1 to ensure that, even in the absence of stimulus drive S (i.e. S_1_ = S_2_ = 0), accumulation of noise alone could reach T within a reasonable number of time steps, while still ensuring that at least several time steps must pass for this to occur (in 10 repetitions of simulations with 10,000 trials each, average median RT = 80.6 and average minimum RT = 7.3). These choices for σ and T formed a fixed reference against which other parameters of the model could be optimized. We found that similar simulation results occurred when using different values for T, which can be readily verified using the simulation code available online (https://github.com/CNClaboratory/Tuned_Inhibition_PLOS_Comp_Bio_2021).

S1.1.2. Fitting S to d’

Next, we determined which values of stimulus drive S yielded simulated d’ values matching the empirical d’ values across the five experimental conditions of Maniscalco et al. [1] (their Figure 4B). To create the dissociation effect, the authors used an unusual design in which noisy gratings presented on one side of the screen (left or right) always had the same contrast C_A_, whereas gratings presented on the other side of the screen could take on one of five possible contrasts C_B,i_, such that C_B,i_ = α_i_C_A_ for 1 ≤ i ≤ 5, where α_i_ < α_i+1_ and α_3_ = 1. Thus, contrasts for C_B_ spanned a range of values below and above C_A_, with the intermediate value of C_B_ equal to C_A_.

We first found the parameter value for S that would match d’ for the intermediate condition i = 3, in which α_3_ = 1 and C_A_ = C_B_; call this parameter S_A_. We conducted preliminary simulations in order to determine approximate values for S for which the model produced threshold values of d’ (in the range of approximately 0 - 3). On this basis, we defined a search region for S consisting of 10 values linearly spaced between 0.001 and 0.025. For each of these S_test_ values, we conducted 10 repetitions of simulations consisting of 10,000 trials. In each simulation, for half of the trials, S_1_ = S_test_ and S_2_ = 0, and for the other half, S_1_ = 0 and S_2_ = S_test_. Since confidence behavior was not of interest at this stage of the fitting, we set τ = 0. We used simulation results to compute d’ at each value of S_test_, separately for each repetition. We averaged these d’ values across repetitions and then fit a quadratic polynomial to the d’ vs S_test_, which produced a near-perfect fit (S1A Fig). We used the fitted polynomial equation to solve for the value of S_A_ that yielded the target d’ value.

The above procedure allowed us to model d’ in the special case of Maniscalco et al.’s [1] experiment where i = 3 and C_A_ = C_B,i_ using the parameter S_A_. Next, we searched for the parameter values S_B_,i that would allow us to fit d’ in the other four experimental conditions, where C_A_ ≠ C_B,i_ for i ≠ 3. We conducted preliminary simulations in order to determine approximate values of S_B_ for which the model produced values of d’ close to the target d’ values. On this basis, we defined a search region for S_B_ consisting of 10 values linearly spaced between 0 and 2.5*S_A_. For each of these S_test_ values, we conducted 10 repetitions of simulations consisting of 10,000 trials. In each simulation, for half of the trials, S_1_ = S_A_ and S_2_ = 0, and for the other half, S_1_ = 0 and S_2_ = S_test_. Since confidence behavior was not of interest at this stage of the fitting, we set τ = 0. We used simulation results to compute d’ at each value of S_test_, separately for each repetition. We averaged these d’ values across repetitions and then fit a quadratic polynomial to the d’ vs S_test_ data, which produced a near-perfect fit (S1B Fig). We used the fitted polynomial equation to solve for the values of S_B,i_ that yielded the target d’_i_ values for i = 1, 2, 4, and 5.

S1.1.3. Fitting U_r_ to confidence probability distributions and τ to meta-d’

We next searched for what value of τ would yield meta-d’ values matching the empirical meta-d’ observed in the intermediate condition of Maniscalco et al. [1], where i = 3 and C_A_ = C_B_. It was natural to select this single condition for fitting meta-d’, since this is the experimental condition where the two response-conditional meta-d’ curves intersected due to the stimulus symmetry whereby C_A_ = C_B_ (see Fig 2 for a reproduction of the data).

We conducted preliminary simulations in order to determine an approximate range for τ for which the model produced threshold values of meta-d’ (in the range of approximately 0.2 - 1.6, i.e. values roughly in the region 0 < meta-d’ < d’). On this basis, we defined a search region for τ consisting of 10 values linearly spaced between 10 and 100. For each of these τ_test_ values, we conducted 10 repetitions of simulations consisting of 10,000 trials. In each simulation, for half of the trials, S_1_ = S_A_ and S_2_ = 0, and for the other half, S_1_ = 0 and S_2_ = S_A_.

The simulated data yielded a distribution of continuous confidence values C_x_. For each simulation repetition, we selected values for the confidence thresholds U_r_ such that they yielded confidence rating probabilities identical to the empirical probability distribution of confidence across all experimental conditions, averaged across subjects. More formally, we computed U_r_ as

$U_{r} = quantile(C_{x}, \sum_{i=1}^{r} P_{data}(conf=i))$ (S1.1)

where quantile(a, p) returns the quantile of the distribution a corresponding to the cumulative probability p and P_data_(conf = i) is the empirical probability distribution of confidence ratings.

Using the confidence thresholds U_r_, we converted the continuous C_x_ values for each trial to discrete confidence rating probabilities on a 4-point scale and then used these ratings, in conjunction with information about stimulus and perceptual decision on each trial, to compute meta-d’ at each value of τ_test_, separately for each repetition. We averaged these meta-d’ values across repetitions and then fit a quadratic polynomial to the meta-d’ vs τ_test_ data, which produced a near-perfect fit (S1C Fig), and used the fitted polynomial equation to solve for the value of τ that yielded the target meta-d’ value. Because the model only works with non-negative integer values of τ, we rounded the fitted τ value to the nearest integer.

To provide a direct test of the necessity of computing confidence from the unnormalized absolute evidence accumulator units x_i_ rather than the differencing units δ_i_, we repeated the above procedure, this time computing confidence values from C_δ_ rather than C_x_. Call the parameter fitted in this way τ_δ_. S1D Fig shows the curve fitting results for meta-d’ vs τ_δ_ for this alternative model.

S1.1.4. Using parameter fits to perform the simulations of Fig 2

With parameter values derived from the procedures described above, we performed a full simulation of the Maniscalco et al. [1] data set. The simulation consisted of 50 repetitions of sets of 10,000 trials, with results averaged across repetitions. Mirroring the design of Maniscalco et al. [1], for half of the simulated trials, S_1_ = S_A_ and S_2_ = 0. For the other half, S_1_ = 0 and S_2_ = S_B,i_ for 1 ≤ i ≤ 5, such that the five possible values of i were equally frequent. By definition, S_B,3_ = S_A_. We used the value of τ fitted to meta-d’ in the intermediate stimulus strength condition i = 3 (Section S1.1.3) for all trials; thus, meta-d’ in all other conditions (i ≠ 3) was not constrained by a fitting procedure, but was allowed to emerge naturally from the behavior of the model fitted exclusively to the intermediate condition (i = 3) where the two response-conditional meta-d’ curves intersect.

The simulated data yielded a distribution of continuous confidence values C_x_. Similar to the procedure described in Section S1.1.3, we selected values for the confidence thresholds U_r_ such that they yielded confidence rating probabilities across the entire simulated data set that were identical to the empirical probability distribution of confidence across all experimental conditions, averaged across subjects.

**S1.2. Simulations for Koizumi, Maniscalco, & Lau 2015 [2], Experiment 1A**

S1.2.1. Selecting values for σ and T

Following the reasoning described above, we set accumulation noise σ = 0.1 and decision threshold T = 1.

S1.2.2. Fitting S_PE_ to d’

For each level of α in [0.1, 0.2, …, 0.9], we set S_NE_ = α S_PE_ and conducted preliminary simulations in order to determine approximate values for S_PE_ for which the model produced values of d’ in the range of approximately 0 - 2.5. On this basis, we defined a search region for S_PE_ consisting of 10 linearly spaced values. For each of these S_PE_ values, we conducted 10 repetitions of simulations consisting of 10,000 trials. In each simulation, for half of the trials, S_1_ = S_PE_ and S_2_ = S_NE_, and for the other half, S_1_ = S_NE_ and S_2_ = S_PE_. Since confidence behavior was not of interest at this stage of the fitting, we set τ = 0. We used simulation results to compute d’ at each value of S_PE_, separately for each repetition. We averaged these d’ values across repetitions and then fit a quadratic polynomial to the d’ vs S_PE_ data, which produced a near-perfect fit (similar to S1A and S1B Fig). We used the fitted polynomial equation to solve for values of S_PE_ that would yield a desired target value of d’ for a given value of α.

S1.2.3. Fitting U_r_ to confidence probability distributions and τ to meta-d’

For each of the three levels of α chosen to model the low PE condition (i.e. α = [0.1, 0.3, 0.5]), we next searched for what value of τ would yield meta-d’ values matching the mean meta-d’ across difficulty levels of the Low PE condition of Koizumi et al. [2] (i.e. the average of the meta-d’ values in the “Low PE, Easy” condition and the “Low PE, Difficult” condition; mean meta-d’_low PE_ = 1.103), given the value of S_PE_ that matched the mean d’ in the Low PE condition (mean d’_low PE_ = 1.709). This value of τ would then be used in all other simulations for that level of α. In particular, behavior of confidence in the simulated High PE conditions would depend entirely upon the fit of τ to meta-d’ in the Low PE conditions.

For each level of Low PE α, we conducted preliminary simulations in order to determine approximate values for τ for which the model produced values of meta-d’ in the range of approximately 0.2 - 1.8. On this basis, we defined a search region for τ consisting of 10 values linearly spaced between 10 and 100. For each of these τ_test_ values, we conducted 10 repetitions of simulations consisting of 10,000 trials. In each simulation, for half of the trials, S_1_ = S_PE_ and S_2_ = S_NE_, and for the other half, S_1_ = S_NE_ and S_2_ = S_PE_.

The simulated data yielded a distribution of continuous confidence values C_x_. Similar to the procedure described in Section S1.1.3, above, we selected values for the confidence thresholds U_r_ such that they yielded confidence rating probabilities across the entire simulated data set that were identical to the empirical probability distribution of confidence across all experimental conditions, averaged across subjects.

Using the confidence thresholds U_r_, we converted the continuous C_x_ values for each trial to discrete confidence rating probabilities on a 4-point scale and then used these ratings, in conjunction with information about stimulus and perceptual decision on each trial, to compute meta-d’ at each value of τ_test_, separately for each repetition. We averaged these meta-d’ values across repetitions and fit a quadratic polynomial to the meta-d’ vs τ_test_ data, which produced a near-perfect fit (similar to S1C and S1D Fig), and used the fitted polynomial equation to solve for the value of τ that yielded the target meta-d’ value. Because the model only works with non-negative integer values of τ, we rounded the fitted τ value to the nearest integer.

As before, to provide a direct test of the necessity of computing confidence from absolute evidence accumulator units x_i_ rather than from differencing units δ_i_, we repeated the above procedure, this time computing confidence values from C_δ_ rather than C_x_. Call the parameter fitted in this way τ_δ_ as before.

S1.2.4. Using parameter fits to perform the simulations of Fig 3

With parameter values derived from the procedures described above, we performed full simulations for each possible pairing of α_low PE, i_ and α_high PE, ij_. For each simulation, we used the values of S_low PE_ and S_high PE_ that yielded d’ values matching the mean d’ across difficulty levels for the Low PE condition in Koizumi et al. [2] Experiment 1A; in this way, d’ was precisely matched across the Low PE and High PE condition for all simulations in Fig 3. Each simulation consisted of 10,000 trials. For half of the trials, S_1_ = S_PE_ and S_2_ = S_NE_. For the other half, S_1_ = S_PE_ and S_2_ = S_NE_. We used the value of τ fitted to mean meta-d’ in the Low PE condition (Section S1.2.3) for all trials; thus, confidence and meta-d’ in the High PE conditions were not constrained by a fitting procedure, but were allowed to emerge naturally from the behavior of the model fitted exclusively to mean meta-d’ for the Low PE condition.

The simulated data yielded a distribution of continuous confidence values C_x_. Similar to the procedure described in Section S1.2.3, we selected values for the confidence thresholds U_r_ such that they yielded confidence rating probabilities across the entire simulated data set that were identical to the empirical probability distribution of confidence across all experimental conditions, averaged across subjects.

To provide a direct test of the main model, we repeated the above simulation procedures, but this time computing simulated confidence from C_δ_ rather than C_x_, and using the fitted parameter τ_δ_ rather than τ.

S1.2.5. Using parameter fits to perform the simulations of Fig 4

For simplicity, the simulations of Fig 3 probe model behavior at a single, intermediate level of d’, even though the experimental data actually probed task performance at two levels of task difficulty. To investigate example model fits across difficulty levels, we computed what value of α_high PE_ was needed to fit the (High PE confidence – Low PE confidence) data at α_low PE_ = 0.1, separately for the C_x_ and C_δ_ models. Given α_low PE_ = 0.1 and the corresponding fitted α_high PE_ value, we then fit S_PE_ to d’ at each level of PE (High / Low) and difficulty (Difficult / Easy) following procedures similar to those described above in Section S1.2.2. To fit the confidence data, we used the same procedure for determining values of τ and U_r_ as described in Section S1.2.3.

To fit α_high PE_, we fit a polynomial curve to the simulated (High PE confidence – Low PE confidence) vs α_high PE_ data from the simulation in the α_low PE_ = 0.1 condition (Fig 3A), separately for the main C_x_ and alternative C_δ_ models. We used this fit to compute the value of α_high PE_ for each model that yielded a confidence effect equal to that observed in the empirical data. (For the main C_x_ model, only the first three data points were used in the polynomial fit in order to best match the local behavior of the curve around the values of α_high PE_ yielding confidence effect magnitudes similar to those in the empirical data.) This procedure yielded fitted values of α_high PE_ = 0.25483 for the main C_x_ model and α_high PE_ = 0.8928 for the alternative C_δ_ model. We then compared the simulated and empirical data for the effect of PE level and difficulty level on confidence vs d’ (Fig 4A), meta-d’ vs d’ (Fig 4B), and RT vs d’ (Fig 4C) for the main C_x_ model with α_high PE_ = 0.25483. Similar analyses are presented in Fig 4D, 4E, and 4F for the alternative C_δ_ model with α_high PE_ = 0.8928.

**S1.3. Simulations for Koizumi, Maniscalco, & Lau 2015 [2], Experiment 2B**

S1.3.1. Selecting values for σ and T

Following the reasoning described above, we set accumulation noise σ_low PE_ = 0.1 and decision threshold T = 1 in the Low PE condition. In the High PE condition, we probed model behavior under σ_high PE_ = 0.11, 0.12, …, 0.2 while keeping T = 1.

S1.3.2. Fitting S to d’

For each level of σ, we conducted preliminary simulations in order to determine approximate values for S for which the model produced values of d’ in the range of approximately 0 - 2.5. On this basis, we defined a search region for S consisting of 10 linearly spaced values. For each of these S_test_ values, we conducted 10 repetitions of simulations consisting of 10,000 trials. In each simulation, for half of the trials, S_1_ = S_test_ and S_2_ = 0, and for the other half, S_1_ = 0 and S2 = S_test_. Since confidence behavior was not of interest at this stage of the fitting, we set τ = 0. We used simulation results to compute d’ at each value of S_test_, separately for each repetition. We averaged these d’ values across repetitions and then fit a quadratic polynomial to the d’ vs S_test_ data, which produced a near-perfetc fit (similar to S1A and S1B Fig). We used the fitted polynomial equation to solve for values of S_test_ that would yield a desired target value of d’ for a given value of σ.

S1.3.3. Fitting U_r_ to confidence probability distributions and τ to meta-d’

We next searched for what value of τ would yield meta-d’ values matching the mean meta-d’ in the Low PE condition of Koizumi et al. [2] Experiment 2B (i.e. the average of the meta-d’ values in the “Low PE, Easy” condition and the “Low PE, Difficult” condition; mean meta-d’_low PE_ = 0.762), given the value of S that matched the mean d’ in the Low PE condition (mean d’_low PE_ = 0.995). This value of τ would then be used in all other simulations for the High PE condition. In particular, behavior of confidence in the simulated High PE condition would depend entirely from the fit of τ to meta-d’ in the Low PE condition.

We conducted preliminary simulations in order to determine approximate values for τ for which the model produced values of meta-d’ in the range of approximately 0.2 - 1 (again, consistent with previous literature). On this basis, we defined a search region for τ consisting of 10 values linearly spaced between 10 and 100. For each of these τ_test_ values, we conducted 10 repetitions of simulations consisting of 10,000 trials. In each simulation, for half of the trials, S_1_ = S and S_2_ = 0, and for the other half, S_1_ = 0 and S_2_ = S.

The simulated data yielded a distribution of continuous confidence values C_x_. Similar to the procedure described in Section S1.1.3, above, we selected values for the confidence thresholds U_r_ such that they yielded confidence rating probabilities across the entire simulated data set that were identical to the empirical probability distribution of confidence across the Low PE condition, averaged across subjects.

Using the confidence thresholds U_r_, we converted the continuous C_x_ values for each trial to discrete confidence rating probabilities on a 4-point scale and then used these ratings, in conjunction with information about stimulus and perceptual decision on each trial, to compute meta-d’ at each value of τ_test_, separately for each repetition. We averaged these meta-d’ values across repetitions and fit a quadratic polynomial to the meta-d’ vs τ_test_ data, which produced a near-perfect fit (similar to S1C and S1D Fig), and used the fitted polynomial equation to solve for the value of τ that yielded the target meta-d’ value. Because the model only works with non-negative integer values of τ, we rounded the fitted τ value to the nearest integer.

To provide a direct test of the necessity of computing confidence from absolute evidence units x_i_ rather than differencing units δ_i_, we repeated the above procedure, this time computing confidence values from C_δ_ rather than C_x_. Call the parameter fitted in this way τ_δ_.

S1.3.4. Using parameter fits to perform the simulations of Fig 5

With parameter values derived from the procedures described above, we performed full simulations for each possible pairing of σ_high PE_ > 0.1 with σ_low PE_ = 0.1. For each simulation, we used the value of S that yielded a d’ value matching the mean d’ across difficulty levels for the Low PE condition in Koizumi et al. [2] Experiment 2B; in this way, d’ was precisely matched across the Low PE and High PE condition for all simulations in Fig 5. Each simulation consisted of 10 repetitions of sets of 10,000 trials, with results averaged across repetitions. For half of the trials, S_1_ = S and S_2_ = 0. For the other half, S_1_ = 0 and S_2_ = S. We used the value of τ fitted to mean meta-d’ in the Low PE condition (Section S1.3.3) for all trials; thus, confidence and meta-d’ in the High PE conditions were not constrained by a fitting procedure, but were allowed to emerge naturally from the behavior of the model fitted exclusively to mean meta-d’ for the Low PE condition.

The simulated data yielded a distribution of continuous confidence values C_x_. Similar to the procedure described in Section S1.3.3, we selected values for the confidence thresholds U_r_ such that they yielded confidence rating probabilities across the entire simulated data set that were identical to the empirical probability distribution of confidence across all experimental conditions, averaged across subjects.

Again, to provide a direct test of the necessity of computing confidence from the absolute evidence accumulator units, we repeated the above simulation procedures, but this time computing simulated confidence from C_δ_ rather than C_x_, and using the fitted parameter τ_δ_ rather than τ.

S1.3.5. Using parameter fits to perform the simulations of Fig 6

For simplicity, the simulations of Fig 4 probe model behavior at a single, intermediate level of d’, even though the experimental data actually probed task performance at two levels of task difficulty. To investigate example model fits across difficulty levels, we computed what value of σ_high PE_ was needed to fit the (High PE confidence – Low PE confidence) data at σ_low PE_ = 0.1, separately for the C_x_ and C_δ_ models. Given σ_low PE_ = 0.1 and the corresponding fitted σ_high PE_ value, we then fit S_PE_ to d’ at each level of PE (High / Low) and difficulty (Difficult / Easy) following procedures similar to those described above in Section S1.3.2. To fit the confidence data, we used the same procedure for determining values of τ and U_r_ as described in Section S1.3.3.

To fit σ_high PE_, we fit a polynomial curve to the simulated (High PE confidence – Low PE confidence) vs σ_high PE_ data (Fig 5A), separately for the main C_x_ and alternative C_δ_ models. We used this fit to compute the value of σ_high PE_ for each model that yielded a confidence effect equal to that observed in the empirical data. This procedure yielded fitted values of σ_high PE_ = 0.1087 for the main C_x_ model and σ_high PE_ = 0.1679 for the alternative C_δ_ model. We then compared the simulated and empirical data for the effect of PE level and difficulty level on confidence vs d’ (Fig 6A), meta-d’ vs d’ (Fig 6B), and RT vs d’ (Fig 6C) for the main C_x_ model with σ_high PE_ = 0.1087. Similar analyses are presented in Fig 6D, 6E, and 6F for the alternative C_δ_ model with σ_high PE_ = 0.1679.

**
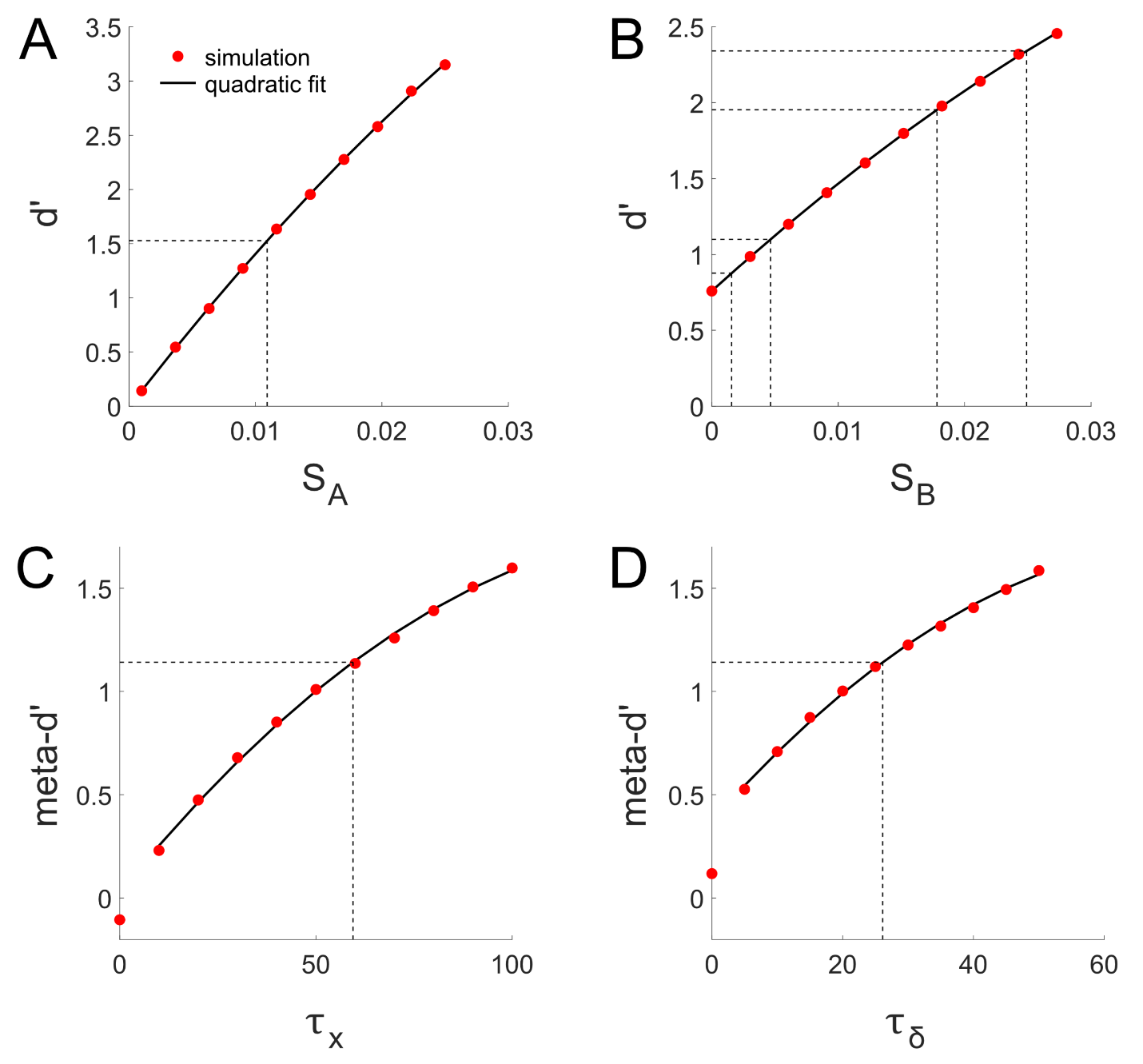
**

**S1 Fig. Parameter fitting for the simulations of Maniscalco, Peters, & Lau 2016 [1].** To fit model parameters to empirical d’ and meta-d’ values, we conducted repeated simulations to estimate the relationship between d’ and S (stimulus drive), as well as meta-d’ and τ (duration of post-decision evidence accumulation). Simulated data are shown with red dots. We then fit quadratic equations to these data (solid black lines), and used the fitted quadratic equations to compute target values of d’ and meta-d’ (dashed lines). Similar fitting procedures were used for the simulations of Koizumi, Maniscalco, & Lau 2015 [2] Experiments 1A and 2B. In panels C and D, we also plot simulated data points for τ = 0 to demonstrate near-chance levels of meta-d’ when there is no post-decision evidence accumulation; these data points were not used for parameter fitting purposes.

**References**

1. Maniscalco B, Peters MAK, Lau H. Heuristic use of perceptual evidence leads to dissociation between performance and metacognitive sensitivity. Atten Percept Psychophys. 2016. doi:10.3758/s13414-016-1059-x

2. Koizumi A, Maniscalco B, Lau H. Does perceptual confidence facilitate cognitive control? Atten Percept Psychophys. 2015. doi:10.3758/s13414-015-0843-3
