# Supplementary material for: Tuned inhibition in perceptual decision-making circuits can explain seemingly suboptimal confidence behavior
Source: PLoS Comput Biol. 2021 Mar 29;17(3):e1008779. doi: 10.1371/journal.pcbi.1008779 (PMC8032199; doi:10.1371/journal.pcbi.1008779)
Supplement: S2 Text — (DOCX) [file pcbi.1008779.s002.docx]

Tuned inhibition in perceptual decision-making circuits can explain seemingly suboptimal confidence behavior

Authors: Brian Maniscalco, Brian Odegaard, Piercesare Grimaldi, Seong Hah Cho, Michele A. Basso, Hakwan Lau, & Megan A. K. Peters

**S2 Text: Additional simulation results and comparison of the C_x_ model to the 2D-SDT model of Maniscalco, Peters, & Lau 2016**

In the main manuscript, we report simulation results whereby the main C_x_ model can capture the dissociation between response-conditional meta-d’ and d’ reported by Maniscalco, Peters, & Lau 2016 [1], whereas the alternative C_δ_ model cannot (Fig 2). Here we present additional simulation results to further investigate why the C_x_ model was successful in fitting the data but the C_δ_ model wasn’t.

As discussed in the main manuscript and explained at greater length in Maniscalco, Peters, & Lau 2016, the dissociation arises due to stimulus strength for stimulus alternative S1 (e.g. contrast for gratings presented on the left side of the screen) being held constant, whereas the other stimulus alternative S2 (e.g. contrast for gratings presented on the right side of the screen) could take on one of five possible values. As S2 stimulus strength increases, so does the overall discriminability between S1 and S2, and so d’ increases. However, if subjects rate confidence using decision congruent evidence, this leads to a counterintuitive situation in which increasing stimulus strength for S2 leads to increasing mean confidence for incorrect “S1” responses even as mean confidence for correct “S1” responses remains constant (see Maniscalco et al. 2016, especially their Figure 3 and related text, for further discussion). Since confidence for correct “S1” responses remains constant and confidence for incorrect “S2” responses increases with increasing S2 stimulus strength, confidence becomes less diagnostic of the accuracy of “S1” responses, and so meta-d’ for “S1” responses decreases.

Thus, the differing abilities of the C_x_ and C_δ_ models to capture the meta-d’ dissociation (Fig 2) comes down to their differing abilities to capture patterns in confidence for incorrect “S1” responses. In S2 Fig we show data and model simulations for mean confidence as a function of S2 stimulus strength, perceptual decision, and accuracy (data are reproduced from Maniscalco et al. 2016 Figure S3; see also their Figure S1). The C_x_ model captures the increase in confidence for incorrect “S1” responses with increasing S2 stimulus strength seen in the data (S2A Fig), whereas the C_δ_ model does not (S2B Fig).

For further insight, using the same simulated data for the Maniscalco et al. 2016 experiment used to create Fig 2, we investigated how confidence evidence was distributed at the time of confidence rating in the C_x_ and C_δ_ models (i.e. x_1_(t_RT_ + τ) and x_2_(t_RT_ + τ) for the C_x_ model, and δ _1_(t_RT_ + τ) and δ_2_(t_RT_ + τ) for the C_δ_ model). Results of this analysis are shown in S3 Fig. The logic of this analysis is that it uses a similar representation to the two-dimensional signal detection theory (2D-SDT) model Maniscalco et al. 2016 used to provide theoretical context for their findings (see their Figures 2 and 3), and therefore could shed further light on why the C_x_ and C_δ_ models differ in their ability to capture the meta-d’ dissociation (Fig 2).

As expected, at the time of confidence rating (t_RT_ + τ) on trials where an S2 stimulus is shown, evidence in the units with a tuning preference for S2 stimuli (x_2_ and δ_2_) increases with increasing S2 stimulus strength (top rows of S3A and S3B Fig, blue contours). Similarly, on trials where an S2 stimulus is shown *and* the subject correctly identifies the stimulus as S2, evidence in the units with a tuning preference for S2 stimuli (x_2_ and δ_2_) increases with increasing S2 stimulus strength (bottom rows of S3A and S3B Fig, green contours). This pattern corresponds to the increase in confidence for correct “S2” responses with increasing S2 stimulus strength that is accurately predicted by both models (S2A and S2B Fig, bottom rows).

Crucially, on trials where an S2 stimulus is shown *and* the subject *in*correctly identifies the stimulus as S1, evidence in the units with a tuning preference for S1 stimuli (x_1_ and δ_1_, i.e. the units used to compute confidence for “S1” responses, in accordance with Eqs. 4 and 5) show diverging patterns as S2 stimulus strength increases (bottom rows of S3A and S3B Fig, red contours).

The activation of x_1_ increases for incorrect “S1” responses as S2 stimulus strength increases (S3A Fig, red contours). Since confidence for “S1” responses is read out from x_1_ in the C_x_ model, this entails that confidence for incorrect “S1” responses increases with increasing S2 stimulus strength (S2A Fig, top row), thus leading to decreasing meta-d’ for “S1” responses (Fig 2A). This pattern is directly analogous to how mean evidence for incorrect “S1” responses increases according to the 2D-SDT model of Maniscalco et al., as illustrated in their Figure 3.

By contrast, the activation of δ _1_ *decreases* for incorrect “S1” responses as S2 stimulus strength increases (S3B Fig, red contours). Since confidence for “S1” responses is read out from δ_1_ in the C_δ_ model, this entails that confidence for incorrect “S1” responses decreases with increasing S2 stimulus strength (S2B Fig, top row), thus leading to increasing meta-d’ for “S1” responses (Fig 2B).

To the extent that the meta-d’ dissociation reported in Maniscalco et al. 2016 is driven by the logic encapsulated in their 2D-SDT model (Maniscalco et al. Figures 2 and 3), it follows that the main C_x_ model considered in the current manuscript is able to capture the meta-d’ dissociation reported in Maniscalco et al. 2016 precisely because the C_x_ model exhibits conceptual and computational similarities to the 2D-SDT model, thereby allowing it to make similar predictions about how task performance should be influenced in the unusual experimental design where S1 stimulus strength is held constant and S2 stimulus strength is varied. In particular, the absolute evidence accumulators x_1_ and x_2_ used to compute confidence in the C_x_ model play similar roles to the two evidence axes of the 2D-SDT model, e_S1_ and e_S2_, and the relative evidence units δ_1_ and δ_2_ used to compute perceptional decision in the C_x_ model play similar roles to the balance of evidence (e_S1_ – e_S2_) in the 2D-SDT model.

**References**

1. Maniscalco B, Peters MAK, Lau H. Heuristic use of perceptual evidence leads to dissociation between performance and metacognitive sensitivity. Atten Percept Psychophys. 2016. doi:10.3758/s13414-016-1059-x


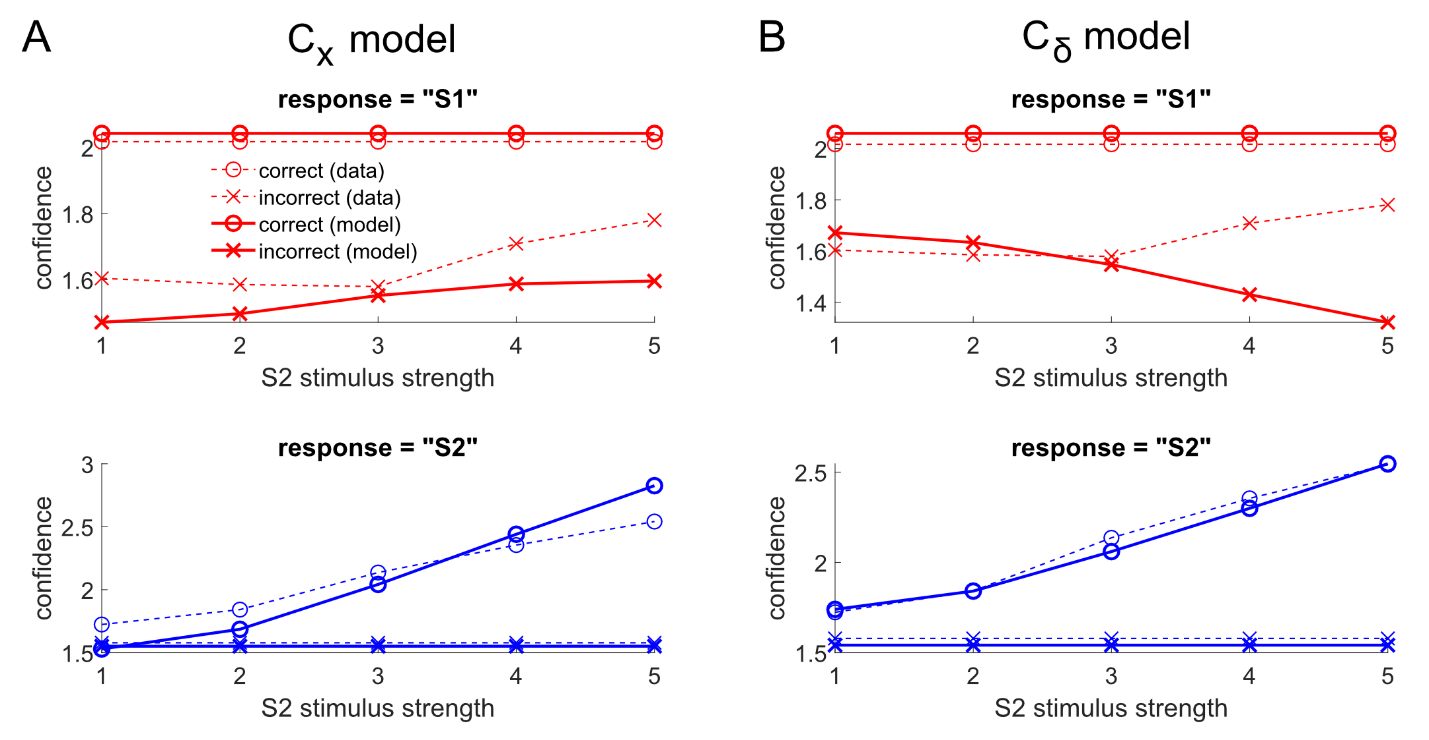


**S2 Fig. Data and simulations for mean confidence as a function of S2 stimulus strength, perceptual decision, and accuracy.** The meta-d’ dissociation reported in Maniscalco, Peters, & Lau 2016 [1] (reproduced in Fig 2) is driven by an increase in mean confidence for incorrect “S1” responses as S2 stimulus strength increases, causing meta-d’ for “S1” responses to decrease even as d’ increases. Thus, the key difference in the main C_x_ and alternative C_δ_ models is their ability to capture this pattern. The C_x_ model is able to predict increasing confidence for incorrect “S1” responses, in agreement with the empirical data (panel a, top row) whereas the C_δ_ model is not (panel b, top row). Compare this figure to Figures S1 and S3 of Maniscalco et al.

**
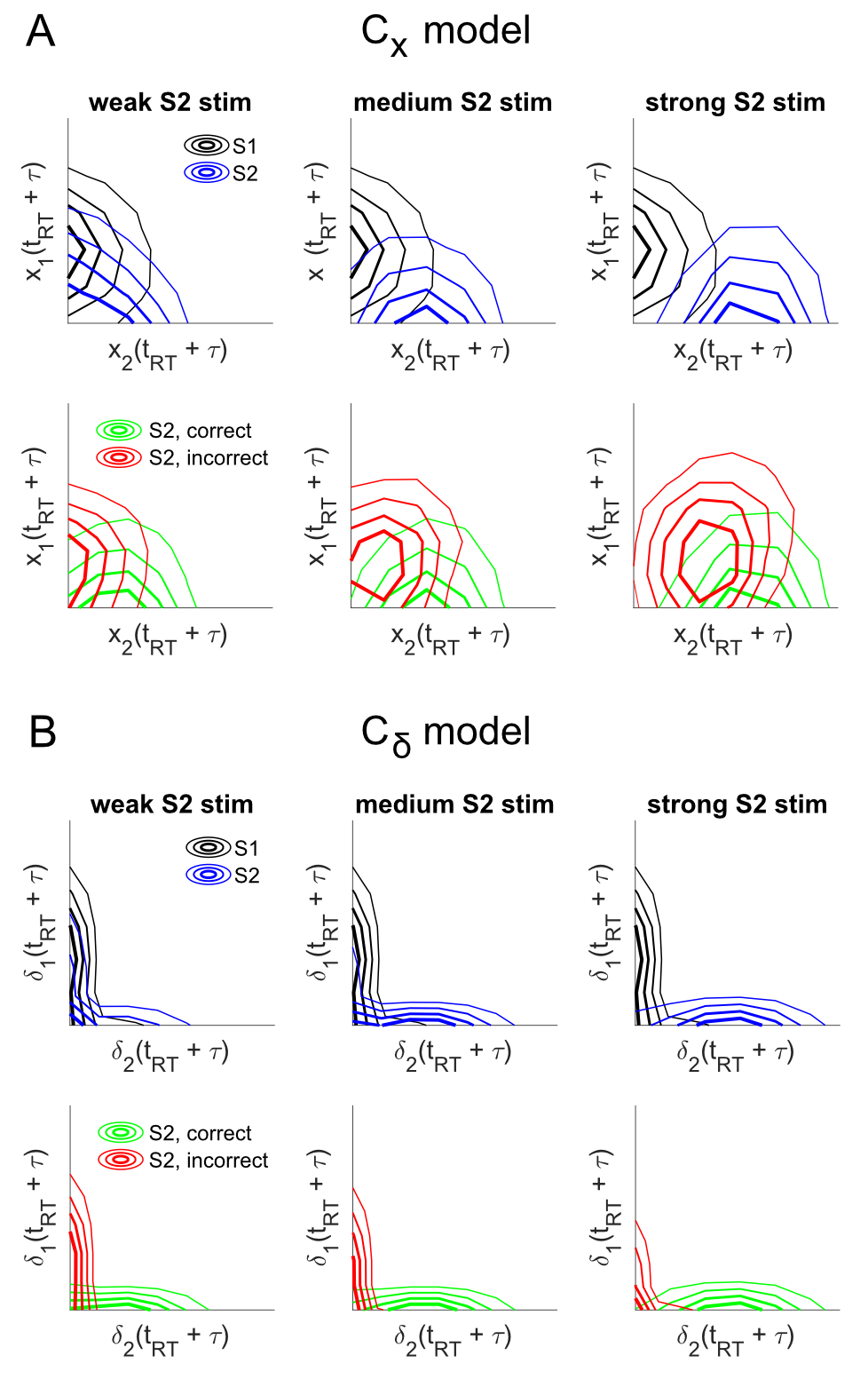
**

**S3 Fig. Simulated distributions of evidence in confidence units at the time of confidence rating as a function of stimulus and accuracy.** We used simulated data for the Maniscalco et al. 2016 [1] experiment to construct distributions of activation in confidence units (x_1_ and x_2_ for the C_x_ model, and δ_1_ and δ_2_ in the C_δ_ model) at the time of confidence rating (i.e. at t_RT_ + τ), by way of analogy to the 2D-SDT model providing the theoretical framework for the experiment of Maniscalco et al. Contours show frequency distributions in the 2D space, analogous to a topographic plot. Top rows show distributions of unit activations for presentation of S1 and S2 stimuli (black and blue contours) as S2 stimulus strength increases. Bottom rows show distributions of unit activations for presentations of S2 stimuli leading to correct (response = “S2”; green contours) and incorrect (response = “S1”; red contours) perceptual decisions. Similar to the 2D-SDT model prediction of Maniscalco et al. (their Figure 3), the C_x_ model predicts that mean activation of the confidence unit x_1_ for incorrect “S1” responses (bottom row of panel A, red contours) increases as S2 stimulus strength increases, which ultimately leads to the meta-d’ dissociation (Fig 2A). By contrast, for the C_δ_ model, mean activation of the confidence unit δ_1_ for incorrect “S1” responses (bottom row of panel B, red contours) decreases as S2 stimulus strength increases, which entails that this model cannot capture the meta-d’ dissociation (Fig 2B).
