# Supplementary material for: Tuned inhibition in perceptual decision-making circuits can explain seemingly suboptimal confidence behavior
Source: PLoS Comput Biol. 2021 Mar 29;17(3):e1008779. doi: 10.1371/journal.pcbi.1008779 (PMC8032199; doi:10.1371/journal.pcbi.1008779)
Supplement: S3 Text — (DOCX) [file pcbi.1008779.s003.docx]

Tuned inhibition in perceptual decision-making circuits can explain seemingly suboptimal confidence behavior

Authors: Brian Maniscalco, Brian Odegaard, Piercesare Grimaldi, Seong Hah Cho, Michele A. Basso, Hakwan Lau, & Megan A. K. Peters

**S3 Text: Testing a Leaky Competing Accumulator implementation of the model on the data of Maniscalco, Peters, & Lau 2016**

In addition to the drift diffusion style model explored in the main manuscript, we also investigated how a leaky competing accumulator (LCA) style model [1] could account for positive evidence biases in confidence rating. Here we focused on modeling the data set of Maniscalco, Peters, & Lau [2] (explored in the main manuscript in the analyses corresponding to Fig 2), as the cross-over pattern of response-conditional meta-d’ in which meta-d’ for “S1” responses decreases with increasing d’ provides a particularly difficult phenomenon for models to capture.

**S3.1. Methods**

S3.1.1. Model specification

The structure of the LCA model is illustrated in S4A Fig. The model includes leaky evidence accumulation units *x_i_* and δ*_i_*, where *i* indexes stimulus tuning. All accumulation units with tuning preference *i* activate an inhibitory interneuron unit *H_i_*, and in turn *H_i_* inhibits the δ*_j_* units with the opposite tuning preference *j*. Thus, the *x_i_* and δ*_i_* units of this LCA model correspond roughly to absolute and relative stimulus evidence, analogous to the *x_i_* and δ*_i_* units in the diffusion style model explored in the main manuscript.

More formally, in each time step the changes in activity of the accumulator units are characterized as

| ${dx}_{i}=\left( S_{i}-\lambda x_{i} \right)\frac{dt}{\tau_{c}}+\varepsilon_{i}\sqrt{\frac{dt}{\tau_{c}}}$ | (S3.1) |
| --- | --- |

| ${d\delta}_{i}=\left( S_{i}-\lambda\delta_{i}-H_{j\neq i} \right)\frac{dt}{\tau_{c}}+\varepsilon_{i}\sqrt{\frac{dt}{\tau_{c}}}$ | (S3.2) |
| --- | --- |

where S_i_ is the momentary magnitude of the stimulus drive to units with tuning preference *i*, $\lambda$ is leak, $H_{j\neq i}$ is the momentary activity of the inhibitory interneuron unit with opposite tuning preference, $\frac{dt}{\tau_{c}}$ is a time scale factor (where $\tau_{c}$ has subscript c to denote it is a time constant, thus differentiating it from the parameter τ which controls the number of time steps in the post-decision confidence rating stage), and $\varepsilon_{i}$ is Gaussian noise with mean zero and standard deviation σ. *H_i_* is computed as the momentary average of the accumulation units with matching tuning preference *i*:

|  |  |
| --- | --- |
| $H_{i}(t)=\frac{x_{i}(t)+\delta_{i}(t)}{2}$ | (S3.3) |

At each time step, accumulator units are updated according to the above equations and rectified, i.e. for the *x_i_* units

| $x_{i}(t) = \max( {x_{i}\left( t-1 \right)+ dx}_{i}(t) , 0 )$ | (S3.4) |
| --- | --- |

and similarly for the δ*_i_* units.

The δ*_i_* units are used to make the perceptual decision; a choice of stimulus alternative *i* is made when δ*_i_* reaches a threshold value of T, and we refer to the time of perceptual decision as t_RT_.

Following the perceptual decision, evidence accumulation continues for an additional τ time steps, at which time a confidence judgment is made. As in the main manuscript, we compare versions of the LCA model in which confidence is based upon units that either do (δ*_i_*) or do not (*x_i_*) receive tuned inhibition, and we similarly name these the C_δ_ and C_x_ variants of the model, respectively. For the C_x_ model, confidence is rated by comparing the activity in the *x_D_* unit corresponding to the perceptual decision *D* at time t_RT_ + τ to a set of confidence thresholds *U_r_*, and the C_δ_ model performs a similar readout from the δ*_D_* unit.

S3.1.2. Parameter selection

We characterize the net leak parameter λ as reflecting the net balance of leak (λ’) and self-excitation (ρ), i.e. λ = λ’ – ρ. On the basis of previous findings [1], we set λ’ = 0.33 and ρ = 0.03, such that net leak λ = 0.3.

In LCA models, the activation of accumulator units over time (assuming zero noise) approaches an asymptote equal to S / λ [1], and the number of time steps required to arrive at a given fraction of that asymptotic value depends only on λ and the time scale factor $\frac{dt}{\tau_{c}}$ . We thus chose to set $\frac{dt}{\tau_{c}}$ = 0.01, which allowed for a generous ~1000 time steps to occur before units achieved approximately 95% of their asymptotic value.

We set decision threshold T = 1 and accumulation noise standard deviation σ = 0.9, since preliminary simulations showed that these values, in conjunction with the stimulus drive parameters discussed below, accomplished the objectives of (1) yielding simulated values for d’ in a range approximately equal to the empirical d’ values in Maniscalco, Peters, & Lau [2], (2) providing a value of T that allowed most decisions to occur prior to units saturating at their asymptotic values, (3) yielding RT values that allowed ample time steps to occur prior to reaching the decision threshold, thus allowing meaningful dynamics to occur (mean simulated RTs in each simulated experimental condition ranged from 74.2 – 87.1 time steps). We set the parameter for number of post-decision time steps prior to confidence rating, τ, equal to 30 as this yielded simulated values for meta-d’ in a range approximately equal to the empirical meta-d’ values in Maniscalco, Peters, & Lau [2]. We set *U_r_* equal to the median of the confidence values generated in each simulation, effectively enforcing a binary confidence rating scale with an equal number of high and low confidence ratings.

Overall, the main focus of these simulations was not to exactly match empirical d’ and meta-d’ values, but rather to investigate to what extent the LCA model could qualitatively capture the response-conditional meta-d’ cross-over effect in Maniscalco, Peters, & Lau [2].

S3.1.3. Simulations

We performed 10 repetitions of simulations consisting of 1,000,000 trials each and averaged simulation results across repetitions. Within each simulation, for half of the trials S_1_ = S_A_ and S_2_ = 0, and for the other half, S_1_ = 0 and S_2_ = S_B,i_ for 1 ≤ i ≤ 5. We set S_A_ = 1 and S_B_ = [0, 0.5, 1, 1.5, 2], reflecting the design in Maniscalco, Peters, & Lau 2016 in which the S_A_ stimulus was constant and the S_B_ stimulus could take on one of five different values.

**S3.2. Results**

S3.2.1. Main results

Simulation results for the C_x_ and C_δ_ models are shown in S5 Fig. Compared to the simulation results of the model explored in the manuscript (Fig 2), the difference between the meta-d’ patterns in the C_x_ and C_δ_ models in the LCA implementation (S5 Fig) is much less pronounced, due to the fact that (1) the LCA C_δ_ model exhibits a smaller (but positive) slope for the meta-d’ vs d’ curve for “S1” responses than for “S2” responses, whereas these two curves are nearly equivalent for the C_δ_ model in the main manuscript (Fig 2B); and (2) the LCA C_x_ model does not exhibit the crucial effect whereby the slope of the meta-d’ vs d’ curve for “S1” responses is negative.

Thus, although the LCA implementation of the model can somewhat capture the meta-d’ cross-over effect, it seems to be more constrained in its ability to capture this effect than the diffusion-style implementation. The LCA implementation also seems to be more limited than the diffusion-style implementation in its ability to capture cases where the meta-d’ vs d’ curves for “S1” and “S2” responses in this experimental design exhibit similar behavior. This latter point is relevant since Maniscalco et al. also found that the meta-d’ cross-over effect can largely disappear, such that meta-d’ vs d’ curves for “S1” and “S2” responses largely overlap (although still with a somewhat smaller slope for “S1” responses), when subjects are provided with performance feedback (their Figure 7).

The limitations in the LCA implementation of the model in capturing these response-conditional meta-d’ effects can be understood in light of the discussion in S2 Supporting Information. There, we summarize Maniscalco et al.’s discussion of how the meta-d’ cross-over effect can be predicted from a two-dimensional signal detection theory (2D-SDT) model where the axes represent absolute evidence for alternatives S_1_ and S_2_, and we further show how the success of the diffusion model explored in the current manuscript can be explained by reference to its ability to create patterns of stimulus evidence distributions in this two-dimensional space similar to those produced by 2D-SDT (S3 Fig). In turn, the similarity in the evidence distributions for the 2D-SDT model and tuned inhibition diffusion model can be traced back to their similar underlying structure, whereby (1) absolute evidence values for S_1_ and S_2_ are collected separately, (2) the decision variable is a simple difference of the two absolute evidence values, and (3) confidence depends on the absolute evidence value for the chosen stimulus alternative.

By contrast, in the LCA implementation, point (2) above breaks down, because the decision variable δ*_i_* is no longer a simple difference of the two absolute evidence variables *x_i_*. Instead, δ*_i_* is now, like *x_i_*, an accumulator unit, and on each time step it accumulates a *momentary* evidence sample for stimulus alternative *i* (S*_i_*) while being inhibited by the *accumulated* evidence for stimulus alternative *j* (H*_j≠i_*), as is evident from eq. S3.2 and eq. S3.3. Additionally, δ*_i_* experiences leak at each time step. Thus, although δ*_i_* is in some sense still computing *relative* evidence for stimulus alternative *i* by virtue of being activated by evidence for *i* and inhibited by evidence for *j*, this relative evidence can no longer be characterized as a simple difference of the absolute evidence for *i* and *j*. This means that the structure of the LCA implementation of the model does not map so cleanly onto the 2D-SDT model, which entails that it generates different distributions of evidence in the absolute evidence space as a function of correct and incorrect perceptual decisions, which entails that it makes different predictions about the meta-d’ cross-over effect.

Distributions of absolute evidence for the LCA model are shown in S6 Fig. Evidently, the non-linear mapping of absolute evidence values *x_i_* onto the decision variables δ*_i_* entails that the two-dimensional distributions of absolute evidence for correct and incorrect responses do not differ much for the C_x_ and C_δ_ models, which in turn accounts for why the response-conditional meta-d’ vs d’ curves for the C_x_ and C_δ_ models do not differ all that drastically (S5 Fig).

S3.2.2. Alternative LCA model

We also explored an alternative LCA model structure, as illustrated in S4B Fig. In this variant of the model, there are no inhibitory interneuron units, but rather the two δ*_i_* units directly inhibit each other. This is functionally equivalent to changing eq. S3.3 above so that H*_i_*(t) = δ*_i_*(t), and thus ensures that the *x_i_* units do not participate in the inhibition of the δ*_i_* units. We found that this model structure produced nearly identical results to those presented in S5 Fig, as shown in S7 Fig.


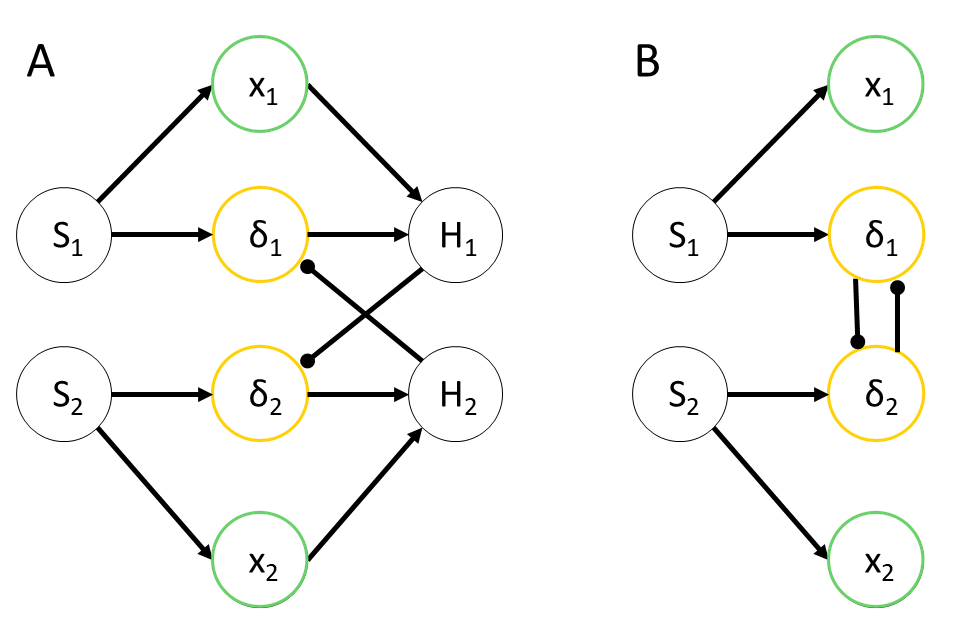


**S4 Fig. LCA model structure. (A)** Both *x_i_* and δ*_i_* units are leaky accumulators of evidence for stimulus S*_i_*, and both activate inhibitory interneuron units H_i_. In turn, δ*_i_* units receive inhibition from the inhibitory interneuron units with opposite tuning preference, H_j≠i_. Thus, the *x_i_* and δ*_i_* units of this LCA model correspond roughly to absolute and relative stimulus evidence, analogous to the *x_i_* and δ*_i_* units in the diffusion style model explored in the main manuscript. **(B)** An alternative model structure which produced simulation results nearly identical to those of the model structure shown in (A).


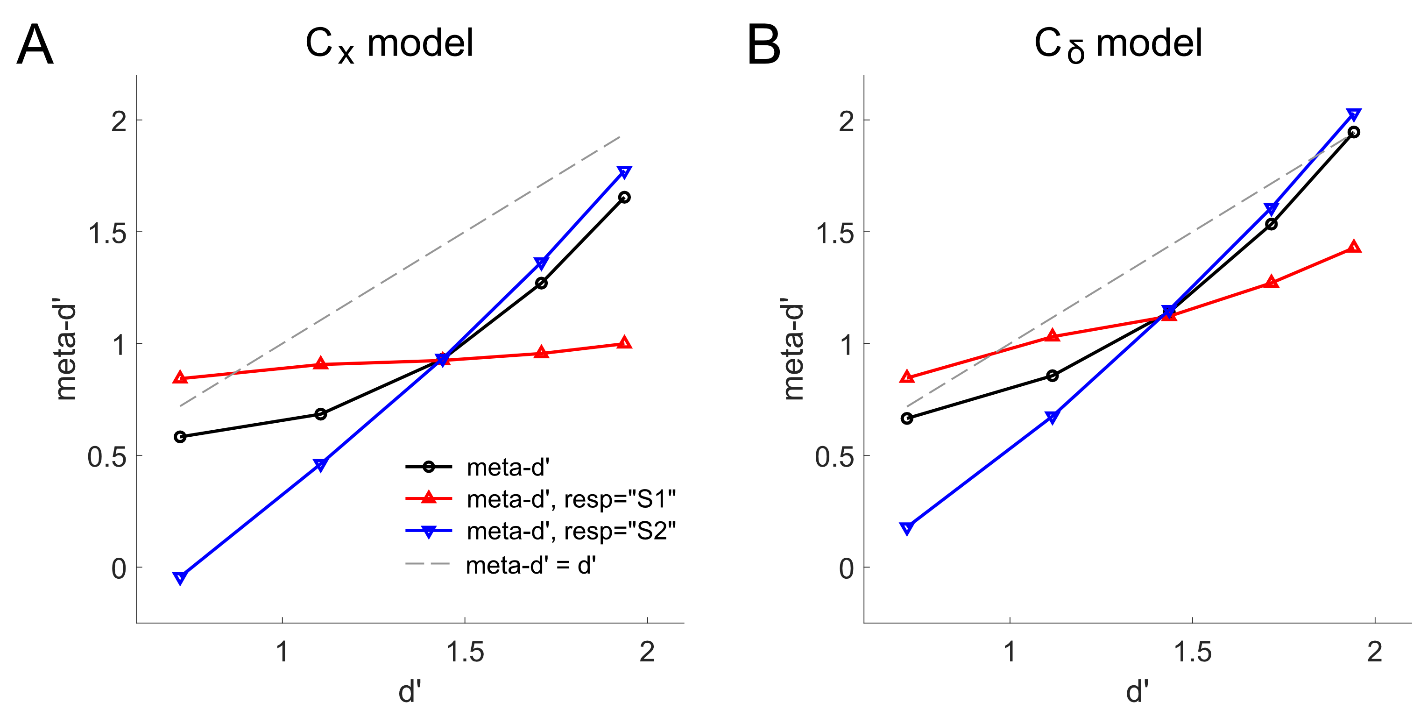


**S5 Fig. Simulation results for the model shown in S4A Fig.**

**
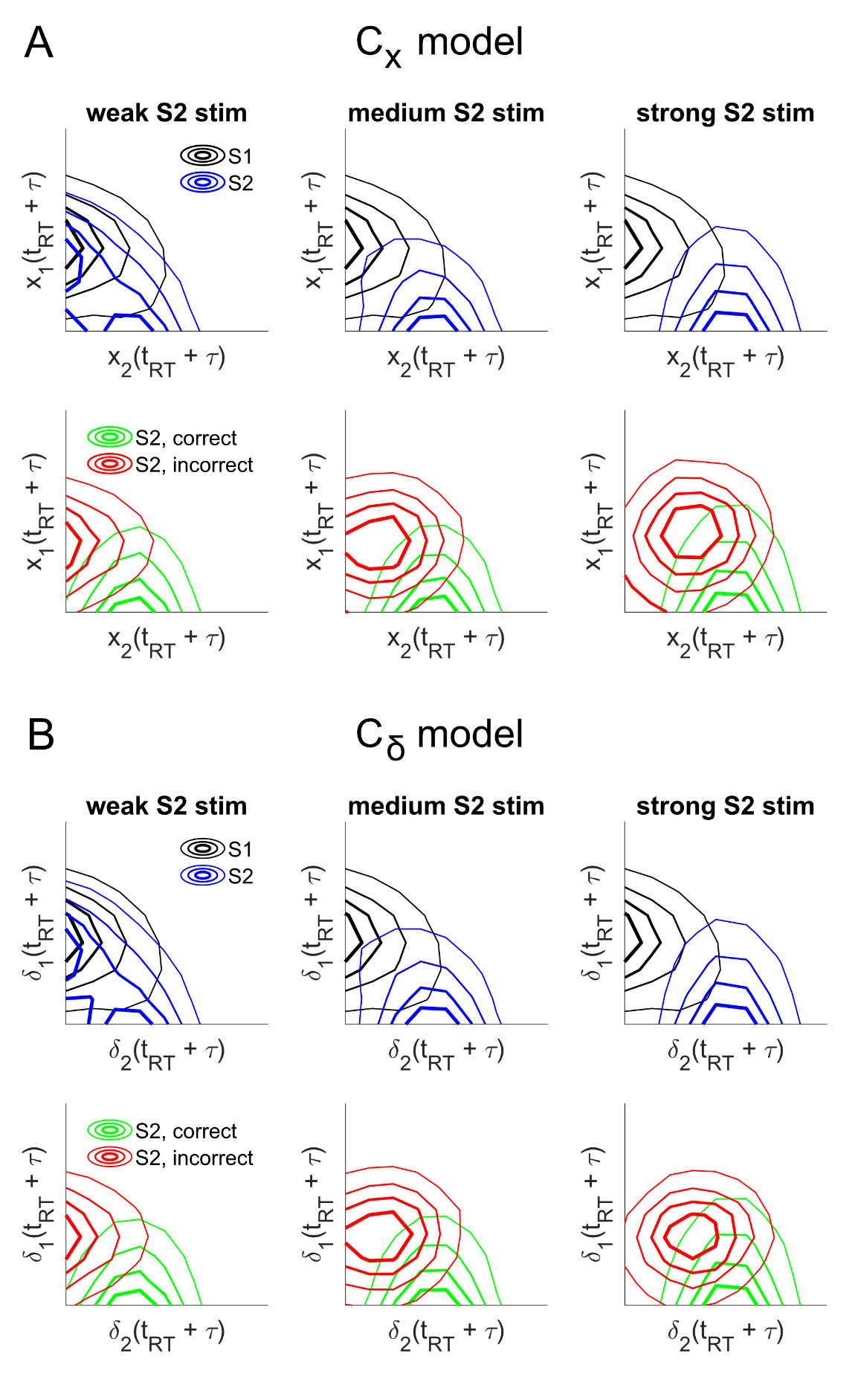
**

**S6 Fig. Simulated distributions of evidence in confidence units at the time of confidence rating as a function of stimulus and accuracy for the LCA model shown in S4A Fig.**

**
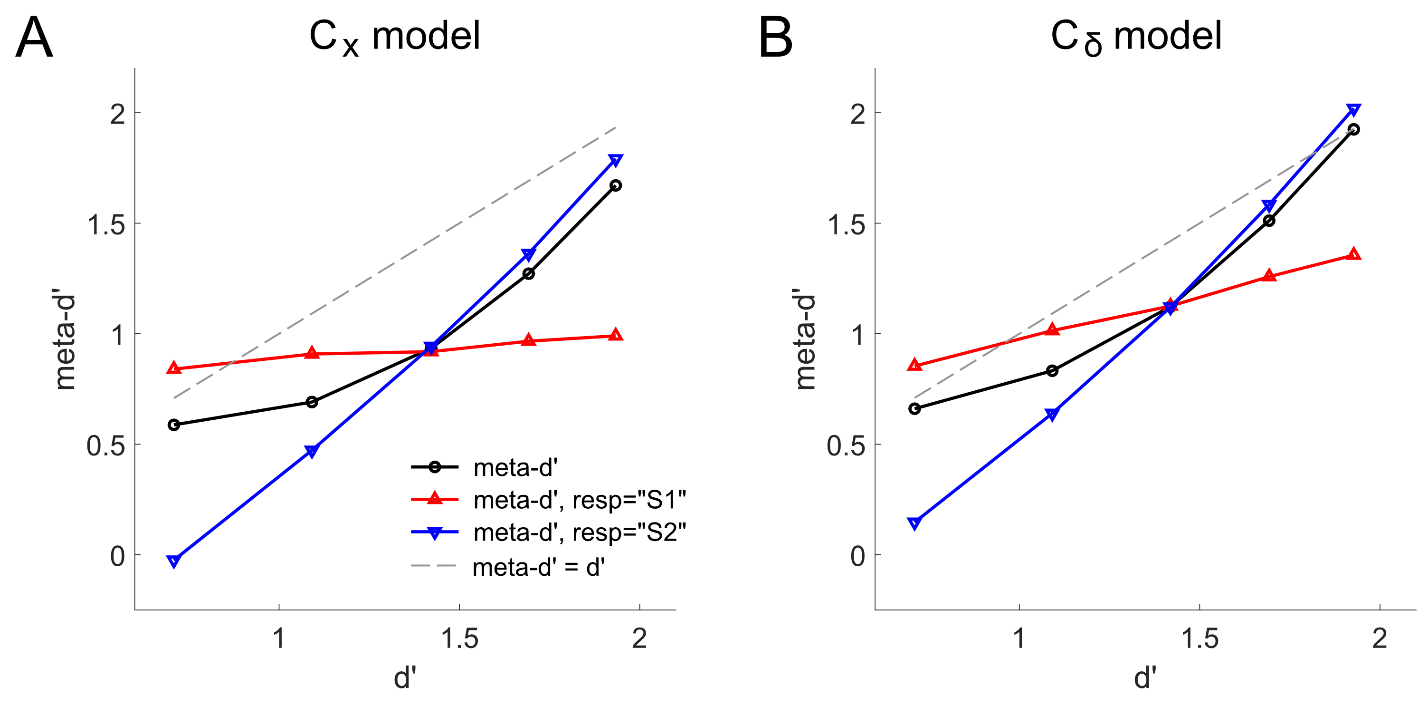
**

**S7 Fig. Simulation results for the model shown in S4B Fig.**

**References**

1. Usher M, McClelland JL. The time course of perceptual choice: the leaky, competing accumulator model. Psychol Rev. 2001;108: 550–592.

2. Maniscalco B, Peters MAK, Lau H. Heuristic use of perceptual evidence leads to dissociation between performance and metacognitive sensitivity. Atten Percept Psychophys. 2016. doi:10.3758/s13414-016-1059-x
