# Supplementary material for: Tuned inhibition in perceptual decision-making circuits can explain seemingly suboptimal confidence behavior
Source: PLoS Comput Biol. 2021 Mar 29;17(3):e1008779. doi: 10.1371/journal.pcbi.1008779 (PMC8032199; doi:10.1371/journal.pcbi.1008779)
Supplement: S4 Text — (DOCX) [file pcbi.1008779.s004.docx]

Tuned inhibition in perceptual decision-making circuits can explain seemingly suboptimal confidence behavior

Authors: Brian Maniscalco, Brian Odegaard, Piercesare Grimaldi, Seong Hah Cho, Michele A. Basso, Hakwan Lau, & Megan A. K. Peters

**S4 Text: Evidence for tuned inhibition in macaque superior colliculus**

**S4.1. Introduction**

Based on the results of our simulations presented in the main text, one may expect that neurons in decision-making areas show this pattern of tuned inhibition. Although tuned inhibition has been observed in primary sensory cortical areas, including V1, V4, and MT/MST [[1–3]](https://paperpile.com/c/FoqrwZ/bZ60b+khpzJ+Hh5Ow), until now it has not been observed in cortical areas known to contain neurons that accumulate evidence (e.g., LIP, FEF) or any subcortical structures, including those with similar function (e.g., superior colliculus; [[4–9]](https://paperpile.com/c/FoqrwZ/MROM+uk2L+mE7d+bSd3+B1xY+KPHz)).

We capitalized on an existing dataset to look for preliminary evidence of tuned inhibition in Rhesus macaque superior colliculus (SC). In a task designed for a different study, one Rhesus macaque engaged in a dot motion direction discrimination task (S8A Fig) while data were recorded from 156 SC neurons using a multi-channel V-probe. The response fields of the SC neurons had previously been mapped, and saccade targets that the monkey could use to indicate its dot motion direction choice were placed either in each neuron’s response field (‘Target’) or in a location on the opposite side of the screen (‘Distractor’). On each trial, a Target and Distractor appeared on the screen followed by centrally-presented dot motion, and the monkey made saccades to the Target or Distractor to indicate its choice (S8A Fig).

‘Catch trials’ of 100% coherent dot motion in the ‘Preferred’ direction (i.e., the direction of motion that should lead to a saccade towards the Target) of each SC neuron, and other catch trials consisting of transparent motion that was 50% ‘Preferred’ and 50% ‘Non-Preferred’ (i.e., the direction of motion that should lead to a saccade towards the Distractor), were interspersed among the trials for that separate study (S8B Fig). We measured the firing rate responses of SC neurons in response to the ‘Preferred’ and 50-50% ‘Preferred + Non-Preferred’ dot motion stimuli in these catch trials, as well as their baseline firing rates, and focused on neurons that exhibited evidence accumulation. We then used these neurons to evaluate whether preliminary evidence for tuned inhibition might be observed in macaque SC. Details of the task, analyses, and results are provided below.

**S4.2. Materials and Methods**

S4.2.1. Behavioral task

The task was a classic dot-motion discrimination task. One monkey observed random dot motion stimuli and made saccades to targets on the screen to indicate its choice about the dot motion direction. On each trial, after an initial fixation period two choice targets appeared for a random time around 500 ms (drawn from an exponential distribution to avoid prediction, S8A Fig). A random dot motion stimulus subsequently appeared at the center of the screen with some percentage of dots moving in the same direction; this is the ‘coherence’ of the dot motion. After the dot motion stimulus offset, the monkey made a saccade either towards the Target (in the recorded neurons’ response field) or towards a Distractor (opposing the response field).

Head restrained monkeys (see below) sat in a custom-sized chair facing a CRT monitor (1024 x 768 pixel resolution, 85 Hz refresh rate) at a distance of 37 cm. A photocell secured to the monitor sent a transistor-transistor logic pulse to the PC used to display stimuli to provide an accurate measure of stimulus event timing. Each trial began by the monkey looking at a white dot that appeared at the center of the monitor. After a brief delay of ~500 ms, randomized from an exponential distribution to prevent prediction, a red target appeared in one hemifield, and a green target appeared in the other. Targets were isoluminant (13 cd/m^2^), and the hemifield in which the red or the green choice target appeared varied randomly from trial-to-trial [[10,11]](https://paperpile.com/c/FoqrwZ/pv1X9+lG9PQ). After a second randomized delay (600-1050 ms), a random dot motion kinematogram stimulus (pattern diameter = 60, 26 cd/m^2^; dot size=0.10; dot separation=0.1820; total density=5 dots/deg^2^) appeared at the center of the monitor together with the white fixation point and remained on the screen for 200 ms. The stimulus’s disappearance was followed by a 500-600 ms delay (the exact value was drawn from an exponential distribution to avoid prediction), at which point the fixation point also disappeared, instructing the monkey to report its choice with an eye movement.

Although there were other trial types presented for the purposes of the experiment for which the data were originally collected, for our analysis we focused on the ‘catch trials’ in which 100% of the dots were moving in the preferred direction (i.e., toward the Target, ‘100% Preferred’ trials) or 50% toward the Target and 50% toward the Distractor (‘50-50% Preferred + Non-Preferred’ trials). These 100% preferred and 50-50% motion ‘catch trials’ were sparsely interspersed among other trial types in the main task, so the monkey completed 154 100% ‘Preferred’ trials and 149 50-50% ‘Preferred + Non-Preferred’ trials in total across eight days. However, because the electrode was placed in a different location on each recording day, on average only 19.25 100% ‘Preferred’ and 18.63 50-50% ‘Preferred + Non-Preferred’ trials were collected for each unit (S1 Table). Unfortunately, electrode placement techniques also precluded precise neuroanatomical localization of the electrode -- and thus the recorded neurons -- on each recording day.

S4.2.2. Electrophysiological recordings

*S4.2.2.a. Surgical procedures*

One male rhesus monkey weighing approximately 10 kg was prepared for electrophysiological recordings and measurement of eye movements. A headpost was implanted to secure the head, and an MRI-compatible recording chamber (Crist instruments, MD) was placed at AP +3, ML 0 and angled 38° posteriorly to access the superior colliculus (SC). Precise positioning of the headpost and the recording chamber was obtained using MRI-guided surgical software (BrainSight, Rogue Research, Montreal, CA). To track eye movements, eye position was monitored with an iView camera (Sensomotoric instruments, Boston, MA). All surgical procedures were performed under general anesthesia using aseptic procedures. Anesthesia was induced with ketamine and midazolam (5.0 mg/kg and 0.2 mg/kg, i.m.). Atropine (0.04 mg/kg, i.m.) was provided to reduce salivation. The monkey was intubated and maintained under general anesthesia with isoflurane. One hour before the procedure, the animal received buprenorphine (0.01 mg/kg, i.m) and the antibiotic Excede (20 mg/kg, i.m; 7 days slow release) and then meloxicam (0.3 mg/kg, i.m) at the conclusion of the procedure. Meloxicam (0.2 mg/kg, i.m) and buprenorphine (0.01mg/kg, i.m) were administered for 3 days post-surgically for multimodal analgesia. All experimental protocols were approved by the UCLA Chancellor’s Animal Research Committee (IACUC, protocol number 2012-043) and complied with and generally exceeded standards set by the Public Health Service policy on the humane care and use of laboratory animals.

*S4.2.2.b. Eye movement recordings*

Experiments used a QNX-based real-time experimental data acquisition and visual stimulus generation system, Rex and Vex, developed and distributed by the Laboratory of Sensorimotor Research National Eye Institute in Bethesda MD [[12]](https://paperpile.com/c/FoqrwZ/2JZS3) to create the behavioral paradigm and acquire eye position data. The camera acquired eye position signals were filtered digitally using a built-in bilateral filter. We used an automated procedure to define the onset of saccadic eye movements using eye velocity (20°/s) and acceleration criteria (5000°/s2), respectively. The adequacy of the algorithm was verified and adjusted as necessary on a trial-by-trial basis by the experimenter.

*S4.2.2.c. Neuronal recordings*

We recorded single neurons and multineuron activity in the SC with a 16 channel platinum/iridium V Probe coated with polyimide (Plexon, Dallas, TX), with an impedance of 275 (±50) kΩ. The V Probe was inserted through a guide tube, perpendicular to the surface of the SC, positioned with a grid system [[13]](https://paperpile.com/c/FoqrwZ/bcVok) and advanced using an electronic microdrive system controlled by a graphical user interface (Nan Instruments, Israel). Action potential waveforms were bandpass filtered (250 Hz - 5 kHz; 4 pole Butterworth), and amplified using the BlackRock NSP hardware system controlled by the Cerebus software suite (BlackRock Microsystems, Utah). Neurons were isolated online using time and amplitude windowing criteria. The times of occurrence of action potentials were digitized at 16 bit resolution and sampled at 1 kHz and saved to disk. Neuronal waveform data were digitized at 16 bit resolution and sampled at 30 kHz and saved to disk for offline analysis using Plexon offline sorting algorithms (Plexon, Dallas, TX).

Response fields (RF) of SC neurons were mapped online. Mapping was done by moving a spot around the monitor and having monkeys make delayed saccades to the different spots. During this mapping procedure, on each trial a fixation spot appeared initially at the center of the screen, and monkeys fixated for 500-1000 ms. A second spot then appeared peripherally while monkeys remained fixated for another 200-400 ms until the fixation spot disappeared; the location of this dot was controlled by mouse movements, and could be anywhere on the screen. Delay times were randomized, drawn from an exponential distribution to prevent prediction. The fixation spot’s disappearance cued the monkey to make a saccade to the peripheral target. If he made a correct saccade (within a window of 2º diameter), he received a fluid reward of 0.1mls.

We listened for maximal discharge for each saccade on-line. The center of the RF was considered to be the location at which a saccade was associated with maximal audible discharge of the neuron. The center of the RF was confirmed by plotting the discharge rate as a heat map in Cartesian coordinates. Only neurons with RF eccentricities between 7 and 20⁰ were selected for further study in order to ensure no overlap of the RF with the centrally-located motion cue stimulus. Although electrode penetration was aimed at the SC perpendicular to its surface, we noticed slight differences in the RF of each recording site of the V-Probe, during the same penetration. Therefore, the RF was optimized for at least one recording site on each experimental day.

| **Day** | **# units** | **# ‘Preferred’ trials** | **# ‘Preferred + Non-Preferred’ trials** |
| --- | --- | --- | --- |
| **1** | 31 | 15 | 13 |
| **2** | 24 | 19 | 16 |
| **3** | 16 | 20 | 20 |
| **4** | 15 | 20 | 20 |
| **5** | 18 | 20 | 20 |
| **6** | 17 | 20 | 20 |
| **7** | 19 | 20 | 20 |
| **8** | 16 | 20 | 20 |

**S1 Table. Daily specifics for recordings from macaque superior colliculus (SC).** Shown are the number of SC neurons recorded after spike-sorting (see Section S4.2, above), and number of trials of ‘Preferred’ and ‘Preferred + Non-Preferred’ stimuli, respectively.

S4.2.3. Calculating inhibition tuning via the Modulation Index

Drawing from previous work [[1–3]](https://paperpile.com/c/FoqrwZ/bZ60b+Hh5Ow+khpzJ), we calculated the inhibition tuning Modulation Index (MI) as:

|  | $MI=\frac{\left( {FR}_{preferred}-{FR}_{baseline} \right)-\left( {FR}_{preferred+non-preferred}-{FR}_{baseline} \right)}{\left( {FR}_{preferred}-{FR}_{baseline} \right)+\left( {FR}_{preferred+non-preferred}-{FR}_{baseline} \right)}$ | (S4.1) |
| --- | --- | --- |

For the baseline firing rate, we took advantage of the fact that the conditions of interest were interspersed in a larger dataset, such that there were many other trials we could use to calculate baseline. Therefore, we calculated each neuron’s baseline firing rate as the average firing rate across all trials (including those in the unrelated task) in the 400 ms prior to the motion cue onset. We calculated the firing rate during the presentation of ‘Preferred’ or ‘Preferred + Non-Preferred’ motion as as the average firing rate across trials during the first 400 ms after motion onset in the relevant trial types. We acknowledge that this definition of Modulation Index in some ways departs from that used in some previous reports to identify inhibition tunedness [[1–3]](https://paperpile.com/c/FoqrwZ/Hh5Ow+bZ60b+khpzJ), and that the stimuli available mean that ${FR}_{preferred + non-preferred}$ will by definition be smaller than ${FR}_{preferred}$ due simply to the fact that reduction in preferred stimulus strength in the former; however, the small number of relevant trials, and the fact that this is an existing dataset, unfortunately precluded adoption of more appropriate stimuli or previously-used metrics.

Action potential waveforms were sorted offline using the Plexon Offline Sorter (Offline Sorter, Plexon Inc). In total, usable signal was recorded from 156 neurons. Because we were interested in the degree to which a neuron modulates its firing rate in the presence of ‘Preferred’ versus ‘Preferred + Non-Preferred’ motion stimuli, we excluded neurons that demonstrated no significant response to dot motion stimulus onset, defined as a firing rate during stimulus presentation equal to or less than the baseline firing rate. This left 125 neurons for the calculation of MI.

We calculated MI for all trials, and also split trials randomly by whether the trial was an even- or odd-numbered trial and calculated MI separately for these subsets of trials. To check for the presence of inhibition tuning, we correlated the MI calculated for even versus odd numbered trials across all neurons.

# S4.3. Results

As expected, neurons showed stronger and earlier spiking for ‘Preferred’ stimuli than for ‘Preferred + Non-Preferred’ (S8C Fig). The average of both of these firing rates in the first 400 ms after stimulus onset were above baseline firing rates (S8E Fig).

Importantly, these trial types can be used to examine inhibition tuning via calculation of a ‘Modulation Index’ (MI) for each recorded neuron (Eq S4.1). Following previous convention [[1–3]](https://paperpile.com/c/FoqrwZ/bZ60b+Hh5Ow+khpzJ), the MI quantifies the degree to which an accumulation neuron with a given tuning preference (i.e., response field) exhibits modulation in its firing rate due to lateral inhibition when presented with simultaneous ‘Preferred + Non-Preferred’ versus ‘Preferred’ stimuli alone. Smaller MI means a cell is less inhibited, because the neuron’s response to Preferred stimuli is relatively independent of the presence of Non-Preferred stimuli; in contrast, larger MI indicates a cell is more inhibited, because the neuron’s firing rate is strongly reduced in the presence of simultaneous Preferred and Non-Preferred motion over its firing rate to Preferred motion alone.

125 neurons demonstrated meaningful responses to dot motion stimulus presentation and evidence accumulation properties (S8C Fig). The distribution of MI for these neurons was centered above zero (μ = 0.1063, σ = 0.1747) (S8D Fig). Because a Lilliefors test revealed that MI significantly deviated from a normal distribution, we used the Wilcoxon signed-rank test [[14]](https://paperpile.com/c/FoqrwZ/KLeFG) to demonstrate that this distribution is centered significantly higher than zero (z = 6.9224, p < .001). This indicates that SC neurons exhibit normalization or inhibition as expected, congruent with previous reports [[15–22]](https://paperpile.com/c/FoqrwZ/WDv83+afRDj+RKmdQ+HqOZD+HFXrW+KJkgV+zvgAF+YZC8x)*.*

To determine the extent to which fluctuations in MI reflected tuned inhibition (i.e., different and *consistent* degrees of inhibition tuning for each individual neuron) and not just random noise, we split trials into even versus odd trial numbers and calculated MI for each disjoint subset of trials. We then examined the correlation between even and odd trials as a robust way to identify tuned inhibition as distinct from noise. Importantly, this revealed a highly significant correlation between even and odd trials (R = 0.4880, p < .001), indicating that the tuned inhibition exhibited by each neuron is highly consistent across trials (S8F Fig).

These results provide preliminary evidence that tuned inhibition exists in evidence-accumulation neurons in decision-making circuits, suggesting that the Tuned Inhibition model framework proposed here may indeed be capable of implementing the calculation of subjective confidence. We do acknowledge that these preliminary results depend on relatively few trials (in an existing dataset) and in some ways depart from other methods by which tuned inhibition has been identified in previous reports [[1–3]](https://paperpile.com/c/FoqrwZ/Hh5Ow+bZ60b+khpzJ); further, we also note that the observation of tuned inhibition in accumulation neurons does not necessitate that such neurons *must* encode decisions versus confidence in the manner hypothesized here. However, these results provide an initial proof of concept, paving the way for future electrophysiological studies examining the predictions of our model in areas previously related to perceptual confidence, such as LIP [[23]](https://paperpile.com/c/FoqrwZ/oHbo3). These findings also provide the first demonstration of inhibition tuning (as opposed to just the presence of inhibition) in a subcortical structure, as previously it has been observed only in cortical areas [[1–3]](https://paperpile.com/c/FoqrwZ/Hh5Ow+bZ60b+khpzJ).

Raw data are available in [24].


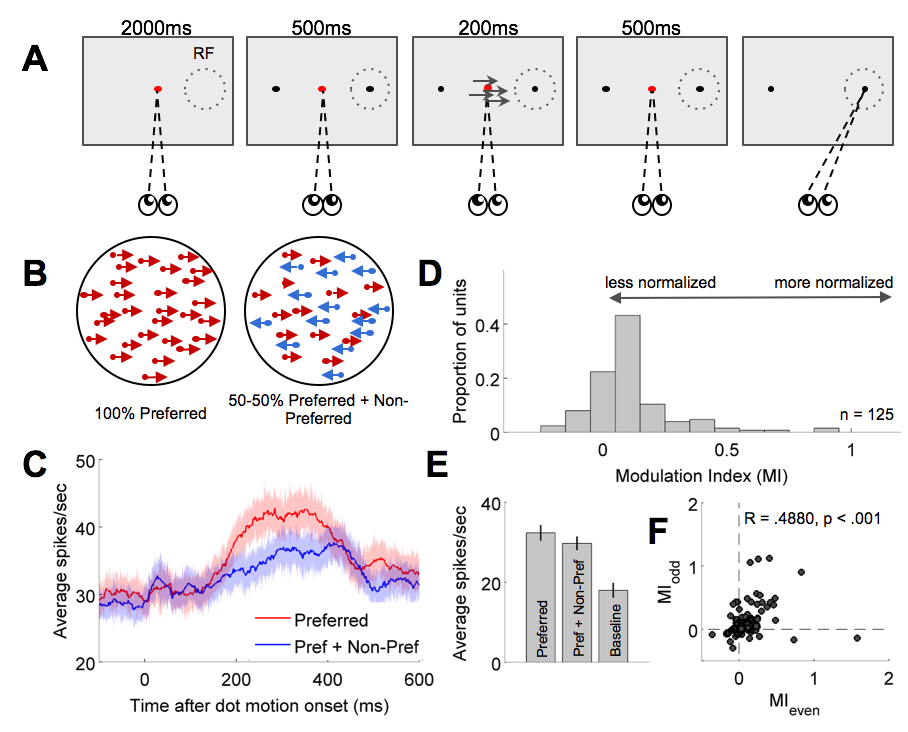


**S8 Fig. Evidence for tuned inhibition in macaque superior colliculus (SC).** (a) We took advantage of an existing dataset in which one monkey performed a dot motion discrimination task. The monkey’s SC neurons’ response fields (RF) were mapped prior to the beginning of the task on each day. On each trial, following a fixation period a brief dot motion stimulus appeared. The monkey observed the stimulus, and then after a delay period made a saccade to the target indicating its dot motion direction discrimination decision. (b) To look for tuned inhibition, we focused on ‘catch trials’ in which 100% ‘Preferred’ motion (indicating a saccade towards the neuron’s RF) or 50-50% ‘Preferred + Non-Preferred’ motion was presented to the monkey. These trials allowed us to calculate each neuron’s Modulation Index (MI; see Section S4.2.3) to measure each neuron’s degree of tuned inhibition. (c,e) As expected, neurons showed higher firing rates to ‘Preferred’ stimuli than to mixed ‘Preferred + Non-Preferred’ stimuli in the post-stimulus window. These were also higher than the baseline (pre-stimulus) firing rate. Mean firing rates in (e) were computed from the 400 ms before (baseline) and after (Preferred and Preferred + Non-Preferred) stimulus onset. (d) We observed a wide distribution of MI values for the SC neurons we recorded, with a median significantly greater than 0 (Wilcoxon sign-rank test: z = 6.9224, p < .001). (f) To confirm that the observed distribution of MI reflected tuned inhibition and not random variation, we split the trials randomly into those with even versus odd trial numbers, and re-calculated MI for each neuron. These disjoint datasets produced MI values that were strongly correlated across even versus odd trials (R = .4880, p < .001), indicating that the degree to which a neuron is inhibited is both *different* across neurons and *consistent* within individual neurons. These results provide evidence of tuned inhibition outside the cortex, and that it exists in evidence-accumulation neurons as opposed to just in primary sensory neurons as previously observed [[1–3]](https://paperpile.com/c/FoqrwZ/Hh5Ow+bZ60b+khpzJ).

**References**

1. [Ruff DA, Alberts JJ, Cohen MR. Relating normalization to neuronal populations across cortical areas. J Neurophysiol. 2016;4: jn.00017.2016–jn.00017.2016.](http://paperpile.com/b/FoqrwZ/bZ60b)

2. [Verhoef B-E, Maunsell JHR. Attention-related changes in correlated neuronal activity arise from normalization mechanisms. Nat Neurosci. 2017; doi:](http://paperpile.com/b/FoqrwZ/khpzJ)[10.1038/nn.4572](http://dx.doi.org/10.1038/nn.4572)

3. [Ni AM, Ray S, Maunsell JHR. Tuned normalization explains the size of attention modulations. Neuron. 2012;73: 803–813.](http://paperpile.com/b/FoqrwZ/Hh5Ow)

4. [Kim B, Basso M. Saccade target selection in the superior colliculus: a signal detection theory approach. J Neurosci. 2008;28: 2991–3007.](http://paperpile.com/b/FoqrwZ/MROM)

5. [Smith PL, Ratcliff R. Psychology and neurobiology of simple decisions. Trends Neurosci. 2004;27: 161–168.](http://paperpile.com/b/FoqrwZ/uk2L)

6. [Gold JI, Shadlen MN. Representation of a perceptual decision in developing oculomotor commands. Nature. 2000;404: 390–394.](http://paperpile.com/b/FoqrwZ/mE7d)

7. [Horwitz GD, Batista AP, Newsome WT. Representation of an abstract perceptual decision in macaque superior colliculus. J Neurophysiol. 2004;91: 2281–2296.](http://paperpile.com/b/FoqrwZ/bSd3)

8. [Ratcliff R, Cherian A, Segraves M. A comparison of macaque behavior and superior colliculus neuronal activity to predictions from models of two-choice decisions. J Neurophysiol. 2003;90: 1392–1407.](http://paperpile.com/b/FoqrwZ/B1xY)

9. [Ratcliff R, Hasegawa YT, Hasegawa RP, Smith PL, Segraves MA. Dual diffusion model for single-cell recording data from the superior colliculus in a brightness-discrimination task. J Neurophysiol. 2007;97: 1756–1774.](http://paperpile.com/b/FoqrwZ/KPHz)

10. [Ferrera VP, Yanike M, Cassanello C. Frontal eye field neurons signal changes in decision criteria. Nat Neurosci. 2009;12: 1458–1462.](http://paperpile.com/b/FoqrwZ/pv1X9)

11. [Bennur S, Gold JI. Distinct Representations of a Perceptual Decision and the Associated Oculomotor Plan in the Monkey Lateral Intraparietal Area. J Neurosci. 2011;31: 913–921.](http://paperpile.com/b/FoqrwZ/lG9PQ)

12. [Hays AV Jr, Richmond BJ, Optican LM. Unix-based multiple-process system, for real-time data acquisition and control. www.osti.gov/scitech/biblio/5213621. 1982; Available:](http://paperpile.com/b/FoqrwZ/2JZS3) <http://www.osti.gov/scitech/biblio/5213621>

13. [Crist CF, Yamasaki DS, Komatsu H, Wurtz RH. A grid system and a microsyringe for single cell recording. J Neurosci Methods. 1988;26: 117–122.](http://paperpile.com/b/FoqrwZ/bcVok)

14. [Wilcoxon F. Individual Comparisons by Ranking Methods. Biometrics Bulletin. 1945;1: 80–83.](http://paperpile.com/b/FoqrwZ/KLeFG)

15. [Basso MA, Wurtz RH. Modulation of neuronal activity in superior colliculus by changes in target probability. J Neurosci. 1998;18: 7519–7534.](http://paperpile.com/b/FoqrwZ/WDv83)

16. [Vokoun CR, Huang X, Jackson MB, Basso M. Response Normalization in the Superficial Layers of the Superior Colliculus as a Possible Mechanism for Saccadic Averaging. Journal of Neuroscience. 2014;34: 7976–7987.](http://paperpile.com/b/FoqrwZ/afRDj)

17. [Phongphanphanee P, Marino RA, Kaneda K, Yanagawa Y, Munoz DP, Isa T. Distinct local circuit properties of the superficial and intermediate layers of the rodent superior colliculus. Eur J Neurosci. 2014;40: 2329–2343.](http://paperpile.com/b/FoqrwZ/RKmdQ)

18. [Schiller PH, Koerner F. Discharge characteristics of single units in superior colliculus of the alert rhesus monkey. J Neurophysiol. 1971;34: 920–936.](http://paperpile.com/b/FoqrwZ/HqOZD)

19. [Sterling P. Receptive fields and synaptic organization of the superficial gray layer of the cat superior colliculus. Vision Res. 1971;11, Supplement 3: 309–IN47.](http://paperpile.com/b/FoqrwZ/HFXrW)

20. [Goldberg ME, Wurtz RH. Activity of superior colliculus in behaving monkey. I. Visual receptive fields of single neurons. J Neurophysiol. 1972;35: 542–559.](http://paperpile.com/b/FoqrwZ/KJkgV)

21. [Moors J, Vendrik AJ. Responses of single units in the monkey superior colliculus to stationary flashing stimuli. Exp Brain Res. 1979;35: 333–347.](http://paperpile.com/b/FoqrwZ/zvgAF)

22. [Li X, Basso MA. Competitive stimulus interactions within single response fields of superior colliculus neurons. J Neurosci. 2005;25: 11357–11373.](http://paperpile.com/b/FoqrwZ/YZC8x)

23. [Kiani R, Shadlen MN. Representation of Confidence Associated with a Decision by Neurons in the Parietal Cortex. Science. 2009;324: 759–764.](http://paperpile.com/b/FoqrwZ/oHbo3)

24. Peters MAK, Maniscalco B, Odegaard B, Grimaldi P, Hah Cho S, Basso M, et al. Tuned inhibition in perceptual decision-making circuits can explain seemingly suboptimal confidence behavior: S4 Data, Evidence for tuned inhibition in macaque superior colliculus. 2020. doi:10.7280/D1T38T
