# Supplementary figures and images for: Tuned inhibition in perceptual decision-making circuits can explain seemingly suboptimal confidence behavior
Source: PLoS Comput Biol. 2021 Mar 29;17(3):e1008779. doi: 10.1371/journal.pcbi.1008779 (PMC8032199; doi:10.1371/journal.pcbi.1008779)

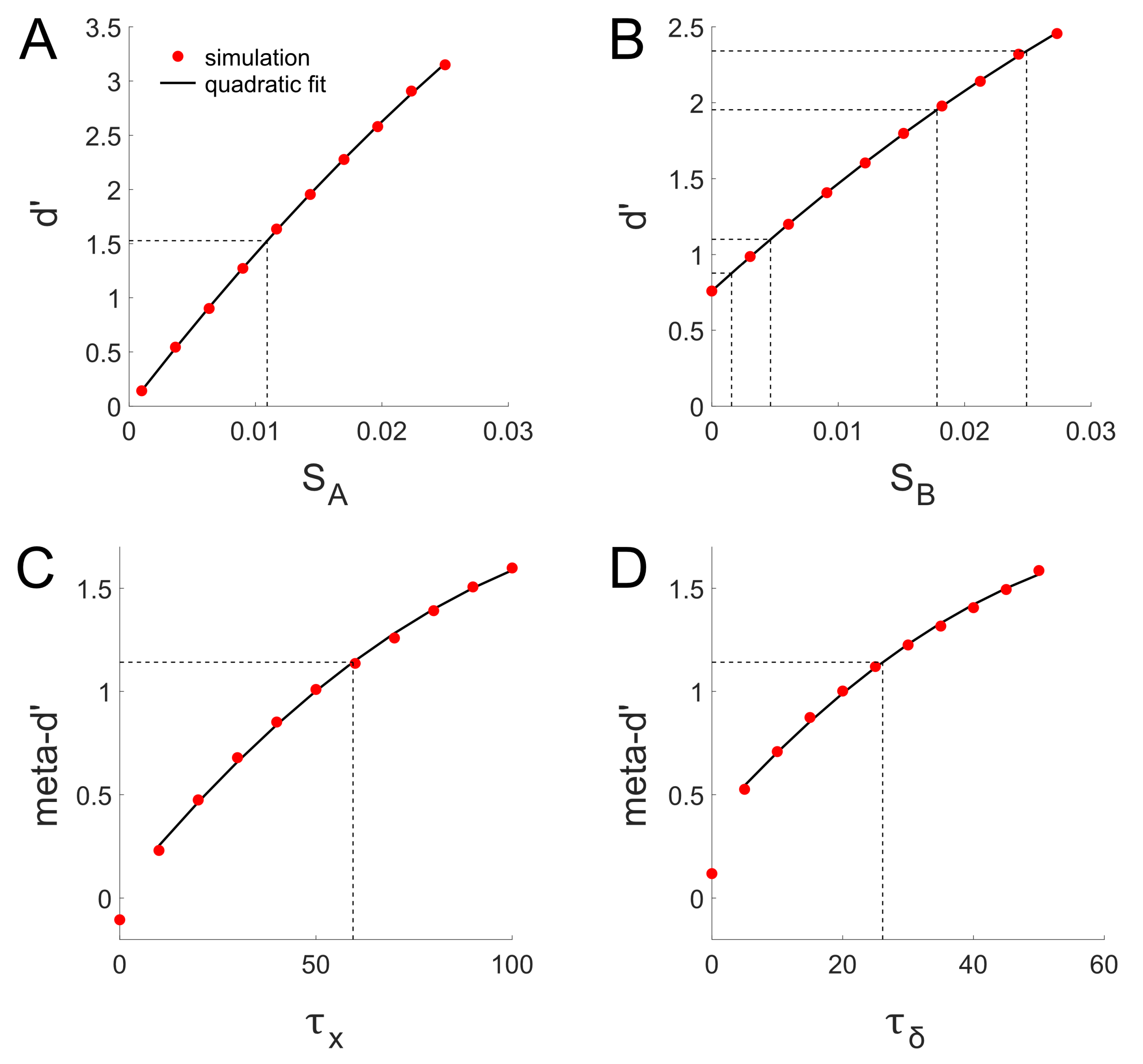

Supplement: S1 Fig — (TIF) [file pcbi.1008779.s005.tif]

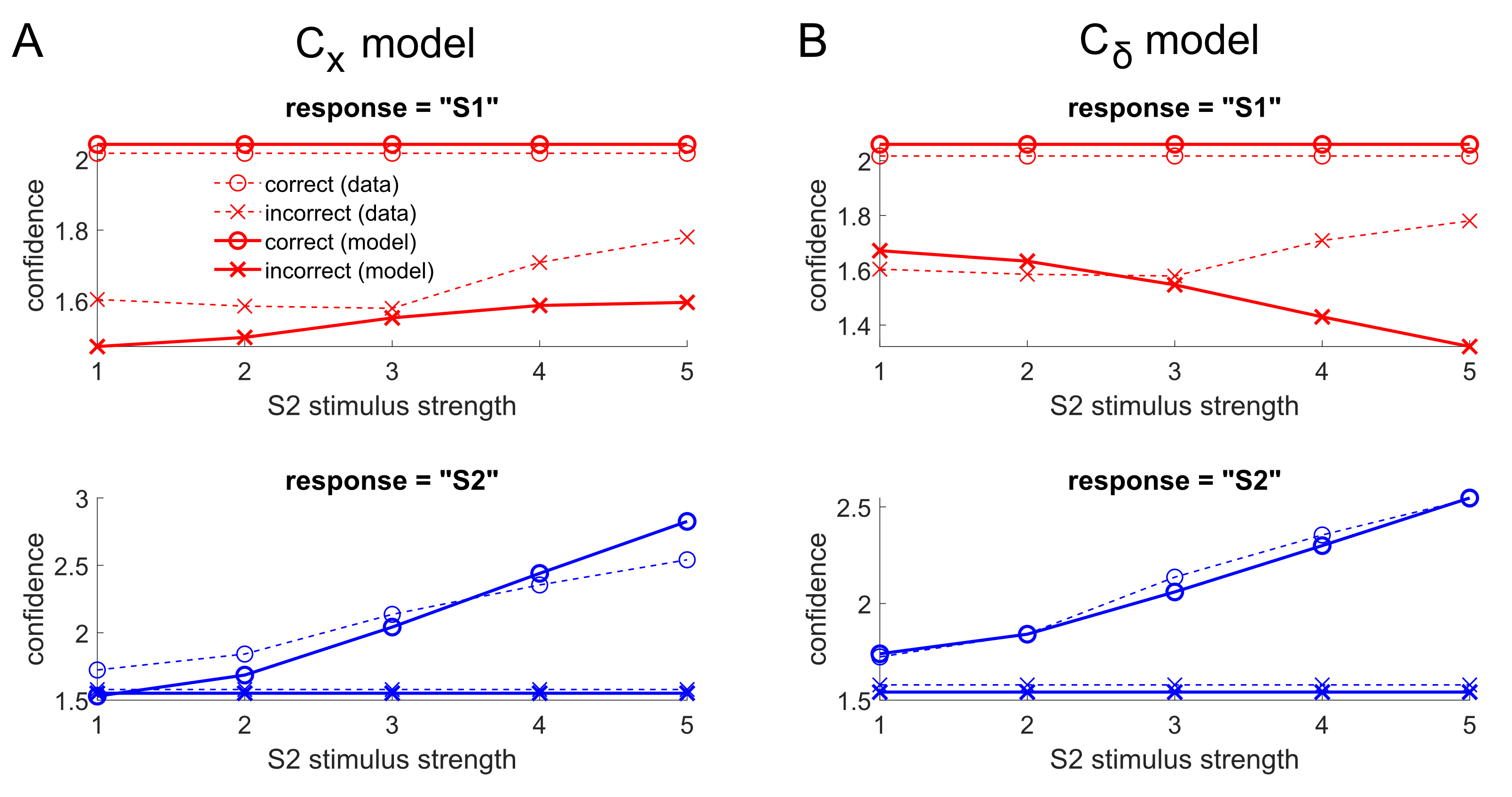

Supplement: S2 Fig — (TIF) [file pcbi.1008779.s006.tif]

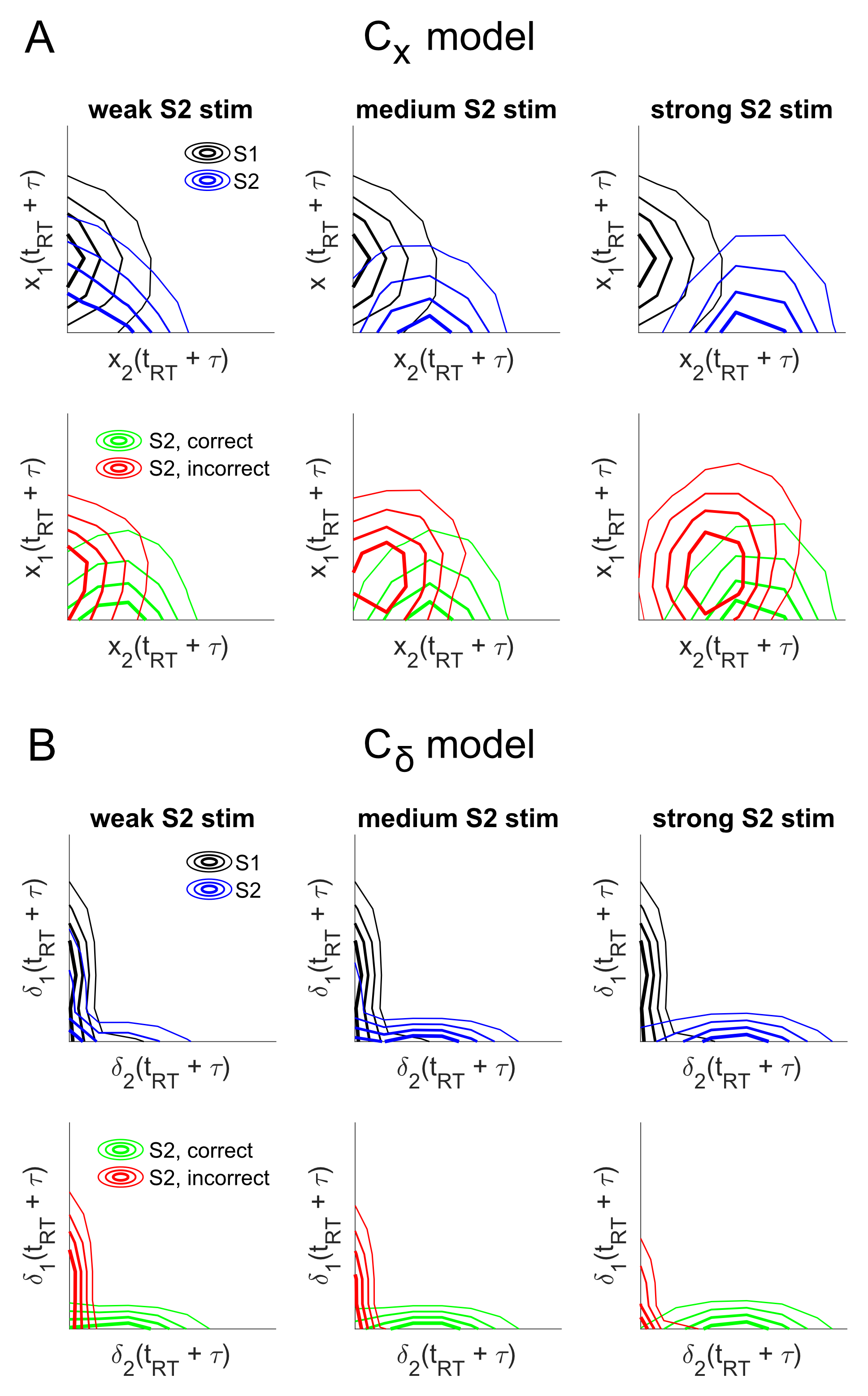

Supplement: S3 Fig — (TIF) [file pcbi.1008779.s007.tif]

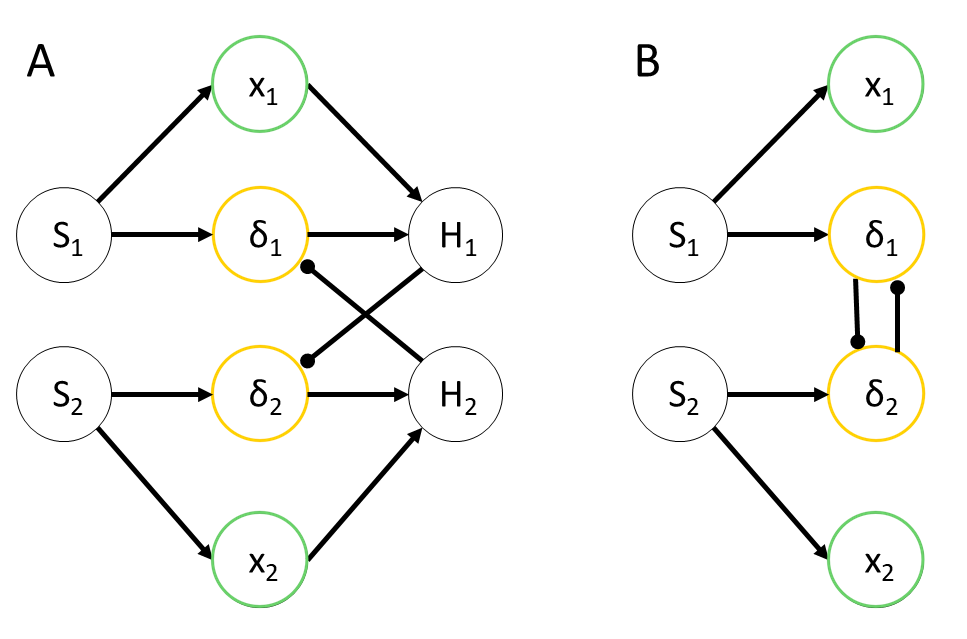

Supplement: S4 Fig — (TIF) [file pcbi.1008779.s008.tif]

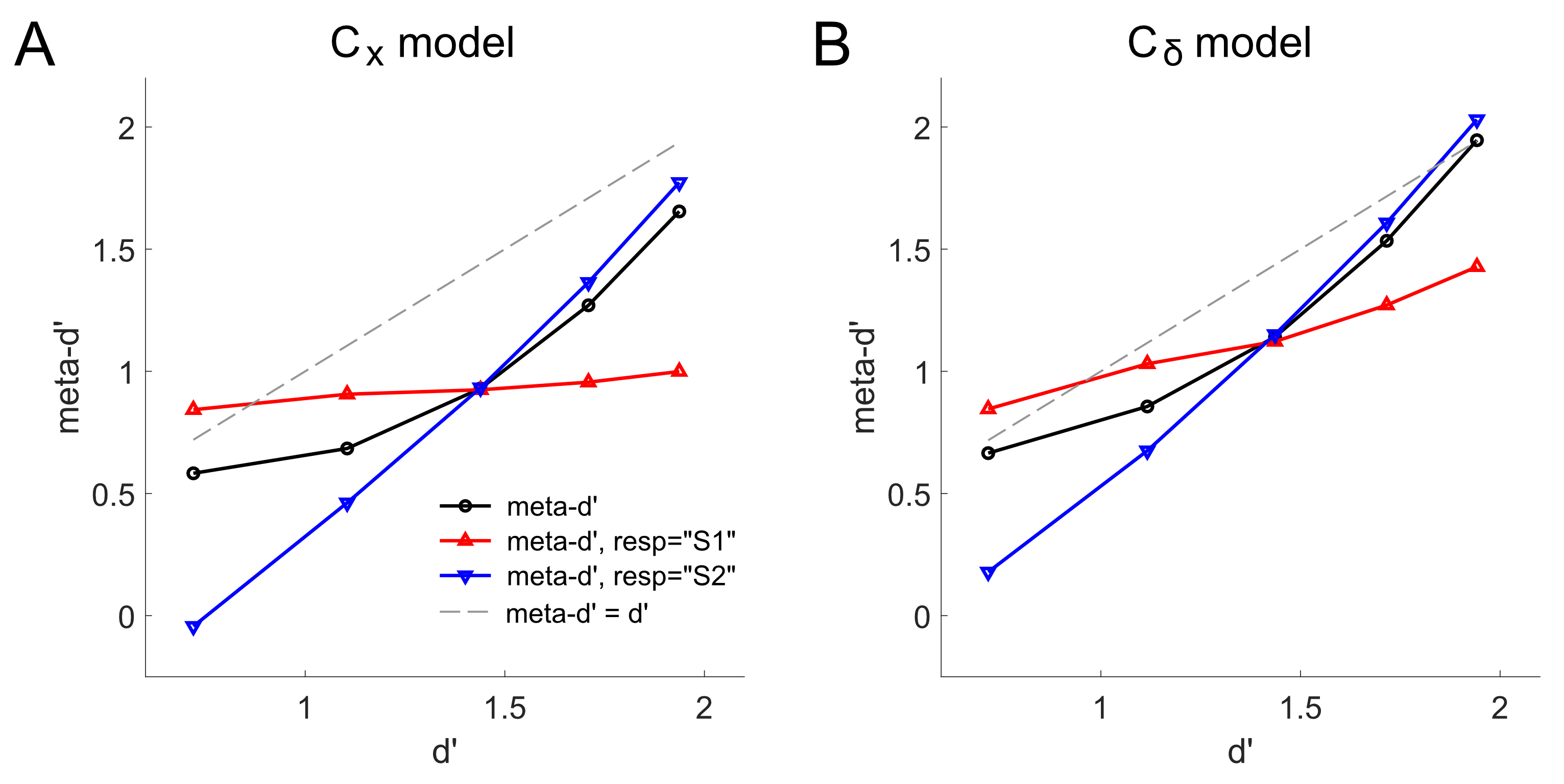

Supplement: S5 Fig — (TIF) [file pcbi.1008779.s009.tif]

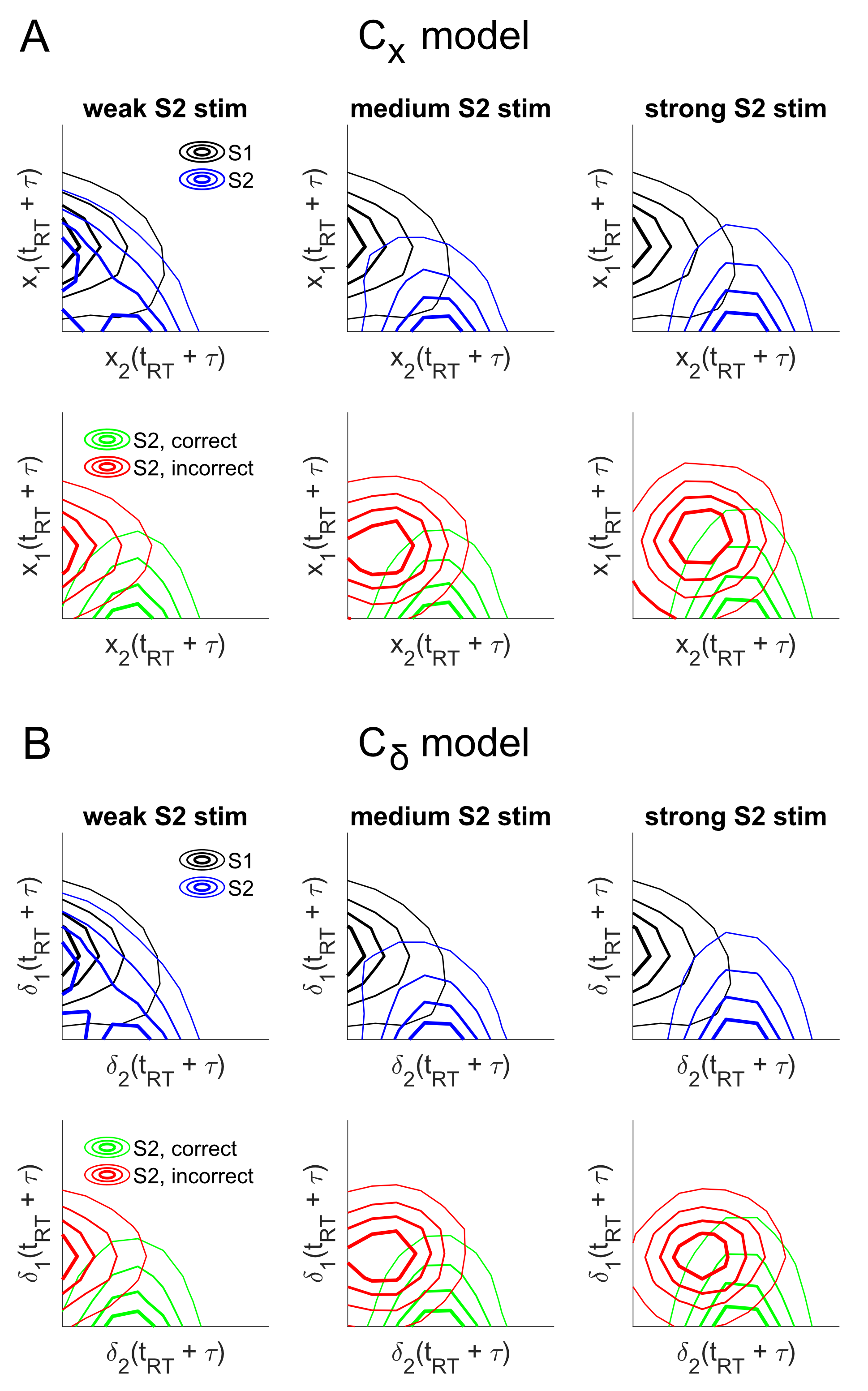

Supplement: S6 Fig — (TIF) [file pcbi.1008779.s010.tif]

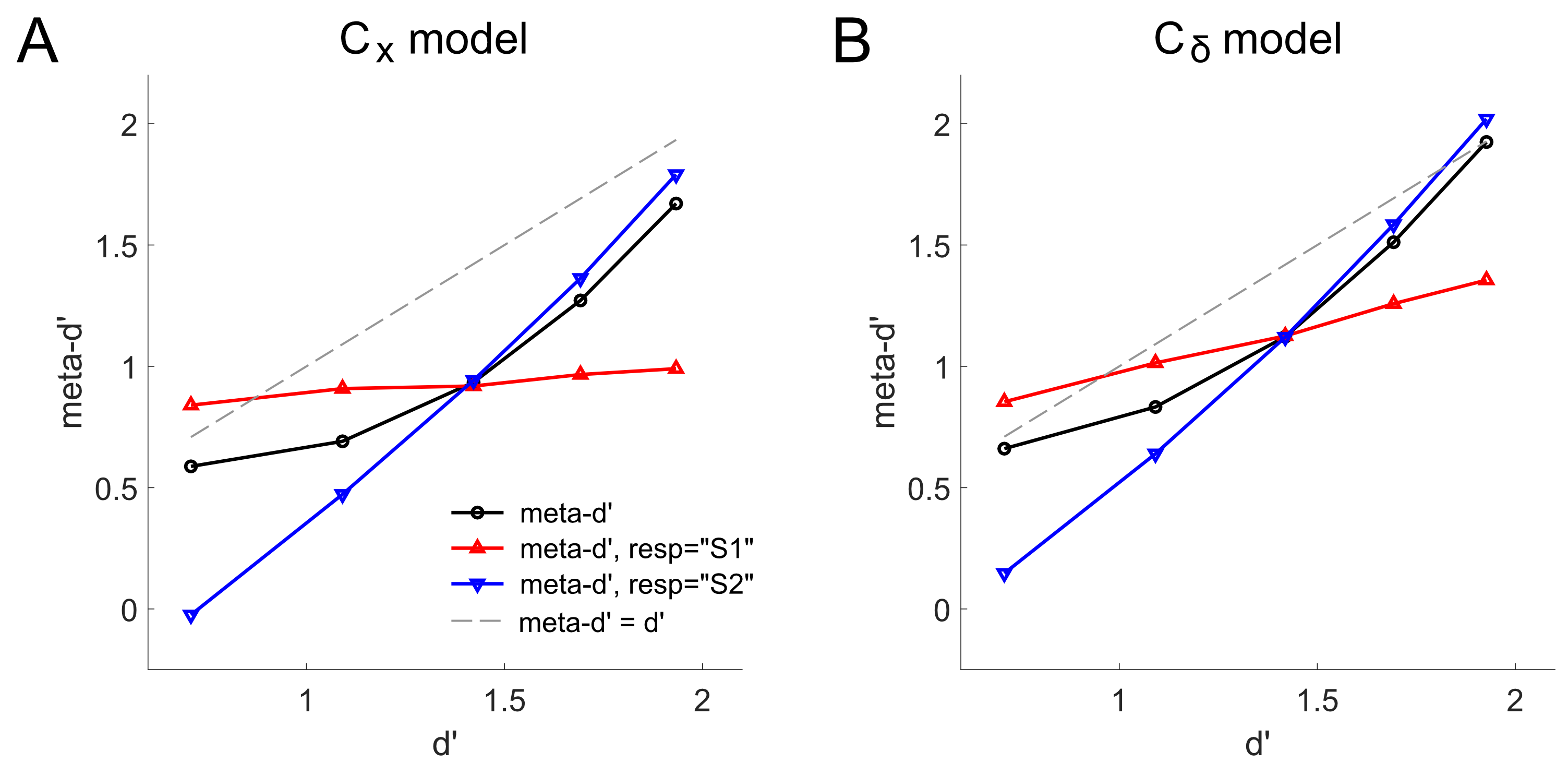

Supplement: S7 Fig — (TIF) [file pcbi.1008779.s011.tif]

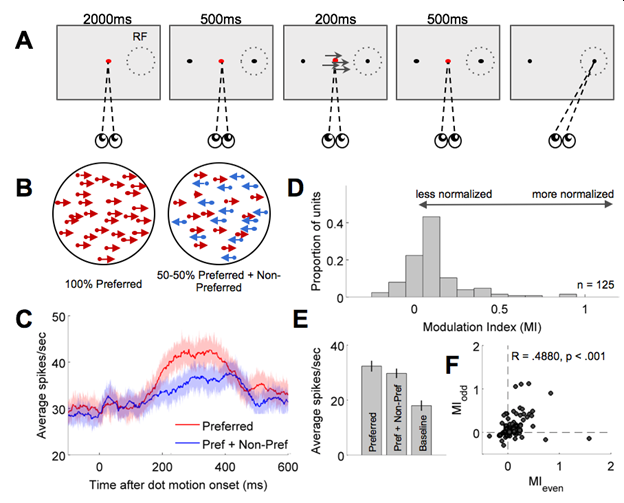

Supplement: S8 Fig — (TIF) [file pcbi.1008779.s012.tif]
